# The HU Regulon Is Composed of Genes Responding to Anaerobiosis, Acid Stress, High Osmolarity and SOS Induction

**DOI:** 10.1371/journal.pone.0004367

**Published:** 2009-02-04

**Authors:** Jacques Oberto, Sabrina Nabti, Valérie Jooste, Hervé Mignot, Josette Rouviere-Yaniv

**Affiliations:** 1 Laboratoire de Physiologie Bactérienne, CNRS, UPR 9073, Institut de Biologie Physico-chimique, Paris, France; 2 INSERM, UMR 866, Epidemiology and Biostatistics group, University of Dijon, Dijon, France; 3 SPSS, Courbevoie, France; University of Munich and Center of Integrated Protein Science, Germany

## Abstract

**Background:**

The *Escherichia coli* heterodimeric HU protein is a small DNA-bending protein associated with the bacterial nucleoid. It can introduce negative supercoils into closed circular DNA in the presence of topoisomerase I. Cells lacking HU grow very poorly and display many phenotypes.

**Methodology/Principal Findings:**

We analyzed the transcription profile of every *Escherichia coli* gene in the absence of one or both HU subunits. This genome-wide *in silico* transcriptomic approach, performed in parallel with *in vivo* genetic experimentation, defined the HU regulon. This large regulon, which comprises 8% of the genome, is composed of four biologically relevant gene classes whose regulation responds to anaerobiosis, acid stress, high osmolarity, and SOS induction.

**Conclusions/Significance:**

The regulation a large number of genes encoding enzymes involved in energy metabolism and catabolism pathways by HU explains the highly pleiotropic phenotype of HU-deficient cells. The uniform chromosomal distribution of the many operons regulated by HU strongly suggests that the transcriptional and nucleoid architectural functions of HU constitute two aspects of a unique protein-DNA interaction mechanism.

## Introduction

HU is a small, basic, and thermostable dimeric DNA-binding protein initially isolated as a factor stimulating the expression of phage lambda genes [1], [2]. It is a major structural component of the nucleoid, and it is conserved among the majority of eubacteria. HU is also present in archaea, in plant chloroplasts, and in a eukaryotic virus [3], [4]. HU of *E. coli* was shown to be a “histone-like protein” which can introduce negative supercoiling into a closed circular DNA in presence of topoisomerase I [5]. We named this protein “HU” where “H” stands for histone and “U” for the U93 strain used at that time to isolate the *E. coli* nucleoid [1], [6]. In most bacteria, HU is encoded by a single gene except in *Enterobaceriaceae* and *Vibrionaceae*, which possess two unlinked HU-encoding genes, *hupA* and *hupB* [4], [7]. In *E. coli*, single *hupA* or *hupB* mutations do not significantly impair growth; however *hupA* inactivation affects survival in prolonged stationary phase [8], [9]. In contrast, the *hupAB* double mutant grows very slowly and is highly pleiotropic: a number of cell processes, such as cell division, initiation of DNA replication, transposition, and other biochemical functions, are altered and cause a slow-growth phenotype [9], [10]. When the absence of HU in *E. coli* cells is not balanced by compensatory mutations in *gyrB*, as frequently observed, the *hupAB* mutant forms very tiny colonies on agar plates [11], [12]. It is interesting to note that the HU mutation is lethal in *Bacillus subtilis*, which has no other histone-like protein [13].

In *E. coli*, the ratio of the three different HU forms, the HUαβ heterodimer and the HUα2 and HUβ2 homodimers, varies as a function of the growth phase [14]. The three dimers exhibit different DNA binding properties towards particular DNA structures [15] and present different thermodynamic properties [16]. We have shown that HU plays a positive role in translation of the stationary phase sigma factor RpoS [17]. This finding was further substantiated by showing direct HU-RNA interaction [18]. *In vitro* studies show that HU displays preferential affinity for damaged DNA having nicks or gaps [15], [19]. Several reports confirm the involvement of HU in DNA repair: (i) cells lacking HU are extremely sensitive to γ and UV irradiation [20], [21]; (ii) HU is capable of displacing LexA, the repressor of the SOS response genes, from its binding sites [22] and (iii) HU binds specifically to a wide array of repair or recombination intermediates [23].

A transcriptional role of HU was also demonstrated for the up-regulation of the *proVWX* operon in hyperosmolar environments [24], and we showed that HU stimulates transcription by T7 RNA polymerase [25]. More recently, Adhya's group revealed a role of HU and negative supercoiling in the formation of the Gal repressosome, a nucleoprotein complex necessary to repress transcription of the *gal* operon [26]. Whereas the respective regulons of other bacterial histone-like proteins, such as Lrp [27], H-NS [28], [29], Fis [30], Crp [31], IHF [32] have been identified, the role of *E. coli* HU on gene regulation has never been addressed systematically at the genomic scale. In the present study, we used microarray hybridization to investigate the pleiotropic role of HU in the cell by studying genome-wide gene expression as a function of the genetic *hupA*, *hupB*, *hupAB* and wild-type backgrounds at three different growth phases. The microarray data, combined with *in vivo* experiments presented here, confirmed the involvement of HU in the SOS and the osmolarity/supercoiling responses [20], [21], [24], [33]. In addition, the results of these experiments revealed a novel function for this global regulator in the environmental programming of the cellular response during aerobic and acid stress. The interconnection between these various responses and the supercoiled state of the DNA is discussed.

## Results

### Microarray experiments

*E. coli* strain C600, originating from the Pasteur Institute, was used for the microarray and *in vivo* experiments described here (JO2057, Table 1). It was preferred over the commonly used ‘wild type’ strain MG1655 for several reasons: first, most of the genetic and biochemical evidence gathered in our laboratory is based on C600 and second, it has been reported that MG1655 suffers a number of growth defects [34] or chromosomal deletions [35]. Due to the instability of *hupAB* mutants [9], [12], special care was taken to reconstruct new mutants starting from JO2057. Strains JO2081 (*hupA*), JO2087 (*hupB*) and JO3020 (*hupAB*) were constructed, and their phenotypes and genotypes were verified, as described in Materials and Methods.

**Table 1:** Strains and plasmids used in this work.

Strain, phage, or plasmid	Relevant characteristic(s) or genotype	Source or Reference
pRS415	*lacZ^+^ lacY^+^ bla^+^*	[87]
λRS45	λ *imm21 ind^+^ bla′-lacZ^+^ lacY^+^*	[87]
λRS88	λ *imm434 ind^−^ bla′-lacZ^+^ lacY^+^*	[87]
OHP109	*hupA::Cm*	[9]
OHP96	*hupB::Km*	[9]
EF88	*Δfnr::*Tn*10 (Tc)*	Jeff Cole
JR1713	*ΔrecA::*Tn*10 (Tc)*	[20]
ENS305	*lacZ::*Tn*10* (*Tc*)	[25]
JO2057 (C600)	*thr-1 leuB6 thi-1 lacY1 glnV44*	Institut Pasteur, laboratory collection
JO2081	*hupA::Cm* (JO2057+P1 transduction from OHP109)	This work
JO2083	*hupB::Km* (JO2057+P1 transduction from OHP96)	This work
JO3020	*hupA::Cm*, *hupB::Km* (JO2081+P1 transduction from OHP96)	This work
JO2039	*lacZ::*Tn*10* (*Tc*) (JO2057+P1 transduction from ENS305)	This work
JO3027	*lacZ* (JO2039 cured from Tn*10* with fusaric acid)	This work
JO3029	*Δfnr::*Tn*10 (Tc)* (JO2057+P1 transduction from EF88)	This work
JO3019	*ΔrecA::*Tn*10 (Tc)* (JO2057+P1 transduction from JR1713)	This work

To identify genes regulated by HU, which is present in *E. coli* as three dimeric forms (HUαβ, HUα2 and HUβ2) at a ratio that varies according to growth phase [14], four strains (the three mutants and the wild type) were grown in LB medium at 37°C. Culture samples for microarray experiments were collected at exponential, transition, and stationary phases. In order to achieve optimal representation of short-lived RNA species, total RNA was extracted from these samples as described in [36]. The genome-wide mRNA levels were measured using high-density *E. coli* Affymetrix® GeneChips microarrays. A total of 16 microarrays were used: 8 assays were performed to duplicate the data for the wild-type and *hupAB* double-mutant strains at exponential and stationary phases; the remaining 8 assays consisted of wild-type and *hupAB* experiments at the transition phase and single *hupA* and *hupB* mutants at the three growth phases. The quality of the microarray data was assessed by statistical analysis of the internal duplicated data, which were found, in each case, to be highly reproducible. After Affymetrix MAS 5.0 processing and normalization, a discriminant criterion derived from fold filters used for gene selection [37] was used to identify genes whose expression varied across the experimental conditions. At that stage, 728 out of the 4368 genes composing the microarray (16% of the genome) were retained. This large amount of genes was certainly due to the combined effects of *hup* genetic background and growth phase. To overcome this difficulty and to identify the genes solely regulated by HU, unsupervised data clustering was performed.

### Biological and statistical validation of the *E. coli* regulon by unsupervised data clustering

Data clustering methods are commonly used to investigate microarray data. However, the relevance of the results is often limited: the number of clusters is not known *a priori* and has to be specified by the user. To identify meaningful classes of genes regulated exclusively by HU, we developed an unsupervised data-clustering method able to avoid numerous single-gene hypotheses by partitioning the transcriptome profiling data into an optimal and biologically relevant number of clusters and by removing the interference of the unwanted growth phase variable.

**Figure 1:**
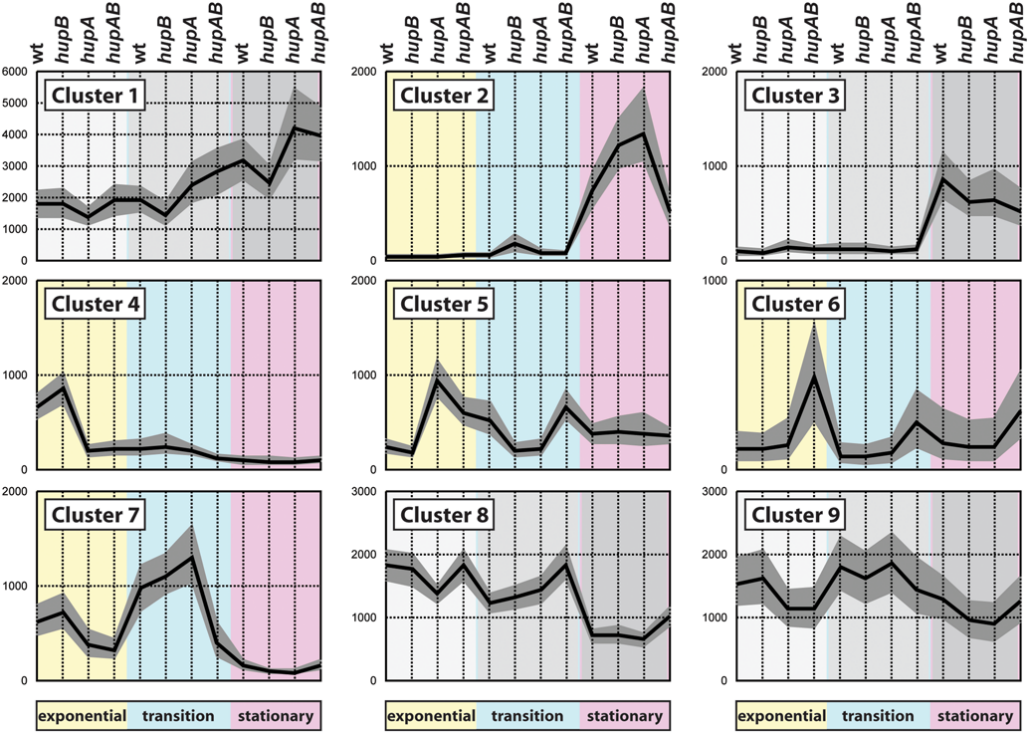
Gene dataset divided in nine clusters. The five clusters shown in color (2, 4, 5, 6 and 7) constitute the HU regulon. The twelve experimental conditions are represented on the x-axis and correspond to the four genotypes (the wild type JO2057; the *hupB* JO2083; the *hupA* JO2081 and the *hupAB* JO3020) at the three growth phases (exponential, transition and stationary). The y-axis indicates the absolute gene expression values for each experimental condition shown in Supplemental Table 2. The black line corresponds to the average values and the grey interval depicts the standard deviation of the bootstrap analysis.

We used the K-means algorithm with a distance measure based on the Pearson correlation to cluster the expression profile of each *E. coli* gene. In our experiments, these profiles were characterized by 12 conditions (4 genotypes at 3 growth phases). The clustering analysis was repeated 24 times for a total number of clusters ranging between 2 and 25. The criterion of Hartigan [38] showed that using nine gene clusters produced the best fit for our data (Fig. 1). We then eliminated unwanted clusters containing genes whose expression varied independently of *hup* genotypes. For this purpose, the Kruskall-Wallis non parametric tests were used. They permitted us to exclude the following clusters: cluster 1, 3 and 8 (growth phase regulated) and cluster 9 (regulated by an undetermined factor) (Supplemental Table S1). It was interesting to note that cluster 8 was populated by a number of genes belonging to the stationary phase sigma factor (RpoS) regulon [39] (Supplemental Table S2). Since we have shown previously that RpoS translation is regulated by HU [17], we decided to exclude from our analysis genes characterized as being under RpoS control.

The five selected clusters (Clusters 2, 4, 5, 6 and 7) amounted to 353 genes (8% of the genome) whose transcription varied in the absence of one or both *hup* genes. These 353 genes constituted the HU regulon. The complete gene list is available in the Supplemental Table S3. With the aid of the **RegulonDB** Web service [40], these 353 genes were found to correspond to 229 operons (Supplemental Table S4). Each of the clusters contained a number of complete transcription units; this certainly constituted a good indication that the clustering analysis was consistent with coordinated expression of the individual genes composing operons. The *proUVW* operon constituted the only exception and will be discussed later. Using the same web resource, each operon of the HU regulon was inspected manually for its respective regulatory characteristic and its potential assignment to other regulons. By comparing theses characteristics with our transcriptomic expression patterns, we were able to assign a specific biological significance to each of the five clusters.

The five HU-regulated clusters were characterized as follows. Cluster 2 was the only one to contain genes induced in stationary phase in strains expressing exclusively HUα2 or HUβ2 homodimers; the transcription of these genes in the double mutant and wild-type strain was similar. Most of the genes of Cluster 2 belonged to well characterized regulatory classes: i) genes induced by acid stress, ii) genes responding to high osmolarity and to supercoiling and iii) genes repressed by FNR. Cluster 4 was found to contain genes activated by HUα2 or HUαβ only in exponential phase; most were FNR activated. Cluster 5 followed an expression pattern opposite to that of cluster 4: its genes were repressed in exponential phase by HUα2 or HUαβ and corresponded mostly to FNR-repressed genes. The genes of cluster 6 were repressed by the HUαβ heterodimer in the three growth phases analyzed. Several of the genes in this small cluster belonged to the LexA-repressed SOS regulon. In cluster 7, the genes appeared to be activated by HUαβ, mainly at the transition phase: they corresponded to genes stimulated by FNR. These clusters are depicted in Figure 1. It should be noted that a number of HU regulated genes encode chaperones or correspond to oxidative stress genes; they are present in the five clusters (Supplemental Table S5). To validate these results, we undertook a more specific analysis and conducted *in vivo* experiments to assess the biological relevance of the five HU regulated clusters.

### HU represses SOS response genes (Cluster 6)

The microarray results showed that transcription of cluster 6 genes was strongly repressed by HUαβ at all three growth phases analyzed (Fig. 1). To facilitate comparisons, absolute expression values from Supplemental Table S3 were normalized to the wild type for each growth phase (Table 2). A number of these genes: *sulA*, *umuD*, *recA*, *recX*, *dinI* and *yebG* encode functions that repair DNA damage and prevent cell division until damage has been repaired [41]; they share the property of being highly induced after UV irradiation and repressed by LexA, the repressor of the SOS regulon. The involvement of HU in the SOS response has been reported by us and others: the extreme sensitivity of cells lacking HU to γ and UV irradiation implies that HU participates in DNA repair, probably via a RecA-dependent pathway [20], [21], [33]. In addition, we reported that the unbalanced over-expression of either HU subunit causes transient SOS induction [42].

**Table 2:** Cluster 6 genes.

Gene	Blattner	Operon	Exponential				Transition				Stationary				Regulation	Function
			WT	*hupA*	*hupB*	*hupAB*	WT	*hupA*	*hupB*	*hupAB*	WT	*hupA*	*hupB*	*hupAB*		
*sulA*	b0958	*sulA*	1	1.98	1.09	19.33	1	1.69	1.25	7.98	1	1.46	1.71	11.39	LexA repressed	suppressor of *lon*, inhibits cell division and *ftsZ* ring formation
*dinI*	b1061	*dinI*	1	1.09	0.72	10.61	1	1.52	1.07	7.34	1	1.25	1.09	14.51	LexA repressed	damage-inducible protein I
*xisE*	b1141	*ymfH-xisE-intE*	1	2.45	0.74	34.01	1	2.34	1.41	7.31	1	0.98	1.06	11.99	LexA repressed	hypothetical protein
*ymfJ*	b1144	*ymfJ*	1	2.67	1.48	41.04	1	2.47	2.41	7.94	1	1.94	1.33	11.7	unknown	hypothetical protein
*ymfL*	b1147	*ymfTLMNROPQ-ycfK-ymfS*	1	4.67	2.23	48.05	1	0.89	1.9	6.32	1	2.11	1.41	10.09	unknown	hypothetical protein
*umuD*	b1183	*umuDC*	1	1.47	1.39	12.64	1	1.63	1.77	5.55	1	1.82	1.51	5.73	LexA repressed	SOS mutagenesis; error-prone repair; processed to UmuD'; forms complex with UmuC
*yebG*	b1848	*yebG*	1	0.56	0.87	2.9	1	1.34	1.29	4.03	1	0.84	0.98	4.75	LexA repressed	hypothetical protein
*recX*	b2698	*recAX*	1	1.69	1.65	6.93	1	2.2	2.07	2.64	1	2.02	2.27	5.01	LexA repressed	regulator, OraA protein
*recA*	b2699	*recAX*	1	0.88	0.95	5.07	1	1.36	0.95	2.41	1	0.95	0.79	4.55	LexA repressed	DNA strand exchange and renaturation; DNA-dependent ATPase; DNA- and ATP-dependent coprotease

SulA is the best known SOS gene; its product binds FtsZ to prevent septum formation in order to inhibit cell division [43]. The constitutive expression of *sulA* in *hupAB* mutants has been reported [44]. Derepression of *sulA* in the *hupAB* genetic background provides an explanation for cell filamentation previously observed [9]. The UmuD protein belongs to an error-prone repair DNA polymerase [45]. DinI and RecX are involved in the positive and negative modulation of RecA filament formation, respectively [46]. RecA, activated by DNA damage, acts as a coprotease assisting LexA repressor autocleavage [30].

Cluster 6 contained, in addition to SOS induced genes, several genes from the cryptic e14 lambdoid prophage: *xisE* (excisionase), *ymfJ* and *ymfL*. These results are compatible with RecA-dependent repressor cleavage and subsequent lytic induction of temperate phages of this family; the SOS-mediated induction of *xisE* and *ymfJ* has been reported [47]. Finally, it was noted that Cluster 6 lacked SOS genes responding more weakly to LexA inactivation, but it included some genes with unrelated or complex regulation, such as *sodA*, which encodes a superoxide dismutase (Supplemental Table S3).

**Figure 2:**
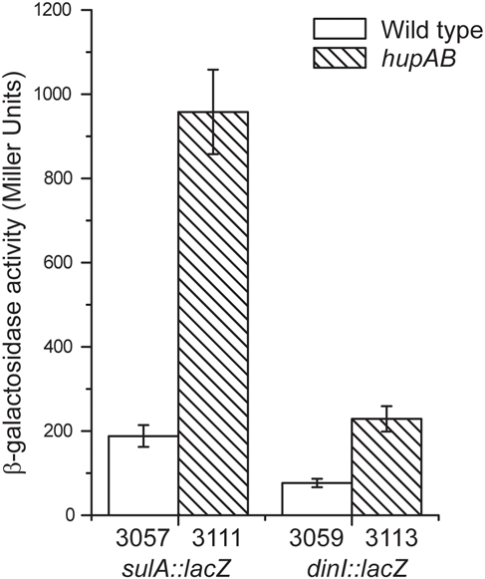
Beta-galactosidase activity of SOS gene fusions. Beta-galatosidase activity of *sulA::lacZ* and *dinI::lacZ* fusions measured in HU^+^ (JO3057, JO3059) and HU^−^ strains (JO3111, JO3113).

**Figure 3:**
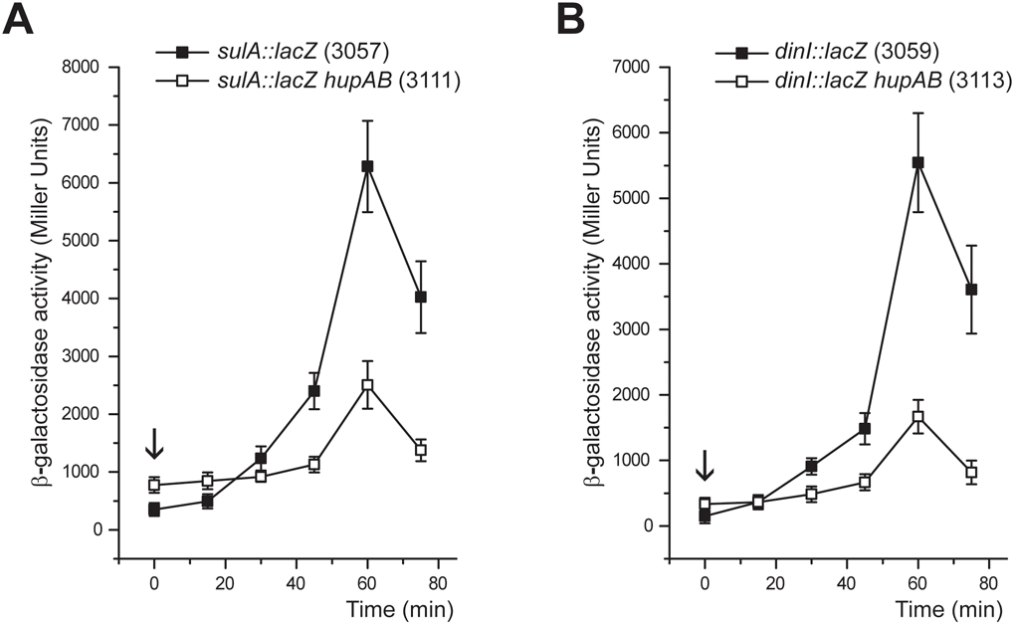
SOS induction kinetics. Respective beta-galatosidase activity of *sulA::lacZ* (A) and *dinI::lacZ* (B) fusions measured in HU^+^ (JO3057, JO3059) and HU- strains (JO3111, JO3113) as a function of time after induction with 50 µg/ml nalidixic acid. The down arrows refer to basal levels (non-induced states), analogous to those observed in the experiment described in Figure 2.

In order to confirm the transcriptome data and to analyze in detail the involvement of HU in the SOS response, we constructed single-copy chromosomal *sulA::lacZ* and *dinI::lacZ* fusions (see Materials and Methods) and analyzed their *in vivo* regulation. Strains JO3057 and JO3059, carrying respectively *sulA::lacZ* and *dinI::lacZ* fusions, were tested for SOS response by an antibiogram plate assay in the presence of nalidixic acid. When XGal was present in the plate, a characteristic blue halo was produced at the edge of the growth inhibition zone. The blue halo was not formed by the respective *recA* mutant derivatives JO3081 and JO3083 (data not shown). In accordance with transcriptome data, the production of β-galactosidase by *hupAB* fusions strains, JO3111 (*sulA::lacZ*) and JO3113 (*dinI::lacZ*), was reproducibly induced three- to five-fold relative to the HU^+^ parental strains, JO3057 and JO3059 respectively (Fig. 2). Similar results have been described for *recA::lacZ* and *umuC::lacZ* fusions [33]. In order to investigate the time course of SOS induction, we measured this response as a function of time, up to 75 min after nalidixic acid induction (Fig. 3). In this experiment, we observed that the SOS response still occurred in a double mutant, as already reported [20], but with a noticeable three-fold lower amplitude. The basal level, before nalidixic acid induction (indicated by an arrow), was higher in the double mutant, as observed in the experiment shown in Figure 2. These results demonstrated that HU is required for a full SOS response.

### HU regulates osmolarity/supercoiling genes (Cluster 2)

Cluster 2 contained a high proportion of genes induced by an increase in osmolarity. Many of these genes have been described previously as belonging to other regulons whose expression is modulated in stationary phase. This last point agrees with our definition of this HU Cluster as seen in Figure 1, the only one to contain genes regulated in stationary phase. The list of cluster 2 genes, with expression normalized to wild-type, is presented in Table 3. The *osmE* gene is regulated by DNA supercoiling and osmolarity [48], and *osmY* is known to be osmotically induced [49]. Under conditions of high osmolarity, the *E. coli otsA* and *otsB* genes are responsible for the synthesis of high concentrations of internal trehalose, an osmoprotectant [50]. The *sra* gene is cotranscribed with the *bdm* gene from a promoter upstream of *bdm* which is activated by osmotic shock [51]. Expression of TalA is induced by osmotic stress only under aerobic conditions [52]. A very strong correlation was observed between these HU regulated genes and genes induced by supercoiling through osmotic shock described in a transcriptomic approach [53]. These genes include genes with known functions: *katE* (catalase hydroperoxidase III), *grxB* (Glutaredoxin), *dps* (required for long-term stationary phase viability), *poxB* (pyruvate oxidase), *wrbA* (NAD(P)H∶quinone oxidoreductase), *aceAB* (isocitrate lyase monomer; malate synthase A) and genes whose function is still under investigation: *elaB*, *ygaM*, *ygaU*, *ybaY*, *ybgS*, *yebV*, *yodC*, *fbaB*. Another recent article reported the proteomic analysis of the osmotic response in *E. coli* [52]; their data corroborate our transcriptome analysis (Table 3).

**Table 3 pone-0004367-t003:** Cluster 2 genes.

Gene	Blattner	Operon	Exponential				Transition				Stationary				Regulation^1^	Function
			WT	*hupA*	*hupB*	*hupAB*	WT	*hupA*	*hupB*	*hupAB*	WT	*hupA*	*hupB*	*hupAB*		
*nhaA*	b0019	*nhaAR*	1	0.82	1.37	1.55	1	1.47	1.63	1.84	1	2.18	1.52	0.82	h	Na+/H antiporter, pH dependent
*ybaY*	b0453	*ybaY*	1	1.5	0.91	1.43	1	1.25	1.98	1.33	1	1.32	1.98	0.46	a	glycoprotein/polysaccharide metabolism
*ybaS*	b0485	*ybaST*	1	0.91	1	1.67	1	1.65	5.04	1.86	1	2.44	1.73	0.5	g	putative glutaminase
*ybaT*	b0486	*ybaST*	1	1.05	1.02	1.32	1	1.53	3.33	1.81	1	1.78	1.54	0.45	c	putative amino acid/amine transport protein
*ybgS*	b0753	*ybgS*	1	0.6	0.96	0.98	1	0.71	0.84	1.31	1	1.73	1.21	0.19	a	putative homeobox protein
*dps*	b0812	*dps*	1	0.7	1.14	0.56	1	1.39	2.57	0.33	1	1.8	1.08	0.58	a, b	global regulator; starvation conditions
*poxB*	b0871	*poxB-ltaE-ybjT*	1	0.64	0.43	0.99	1	1.37	1.03	1.19	1	1.66	1.55	0.38	a, b	pyruvate oxidase
*ycaC*	b0897	*ycaC*	1	0.68	1.67	0.73	1	1.98	1.64	0.75	1	1.4	1.59	0.59	h	hypothetical protein
*cbpA*	b1000	*cbpAM*	1	0.75	0.83	0.66	1	0.89	1.65	0.69	1	1.64	1.52	0.72	h	curved DNA-binding protein, functions closely related to DnaJ
*wrbA*	b1004	*wrbA-yccJ*	1	0.26	1.3	0.33	1	1.37	2.02	0.35	1	0.88	1.17	0.51	a	*trp* repressor binding protein
*grxB*	b1064	*grxB*	1	0.92	1.43	0.86	1	1.29	2.06	0.68	1	1.09	1.2	0.72	a	glutaredoxin 2
*sra*	b1480	*bdm-sra*	1	0.65	0.92	0.86	1	1.77	1.77	1.12	1	1.66	1.2	0.83	a	30S ribosomal subunit protein S22
*bdm*	b1481	*bdm-sra*	1	0.58	0.25	1.18	1	4.13	2.88	13	1	4.11	2.36	0.59	a	biofilm-dependent modulation protein
*gadC*	b1492	*gadBC*	1	0.95	1.33	1.52	1	1.19	5.17	0.91	1	2.35	1.98	0.66	d, e	GadC GABA APC transporter
*gadB*	b1493	*gadBC*	1	0.67	1.3	3.08	1	1.95	30.94	0.41	1	2.71	2.08	0.67	d, e, g	glutamate decarboxylase isozyme
*katE*	b1732	*katE*	1	2.03	1.95	2.09	1	1.2	1.3	1.63	1	1.55	1.7	0.89	a, b	catalase hydroperoxidase HPII(III)
*osmE*	b1739	*osmE*	1	0.91	0.97	1.06	1	1.25	3.82	0.89	1	1.37	1.44	0.52	a	activator of *ntrL* gene
*yebV*	b1836	*yebV*	1	0.81	1.17	1.7	1	1.1	1.35	1.73	1	2	1.93	0.64	a, h	hypothetical protein
*otsA*	b1896	*otsBA*	1	2.53	3.08	0.75	1	1.34	1.81	0.25	1	1.71	1.71	0.73	a	trehalose-6-phosphate synthase
*otsB*	b1897	*otsBA*	1	0.67	1.13	1.12	1	2.26	2.48	2.87	1	2.85	2.16	0.53	a, b	trehalose-6-phosphate phophatase
*yodC*	b1957	*yodC*	1	0.76	0.98	0.98	1	1.52	2.07	1.71	1	2.07	2.22	0.81	a	hypothetical protein
*fbaB*	b2097	*fbaB*	1	1.23	1.04	1.25	1	1.67	2.18	1.18	1	1.27	1.02	1.18	a	fructose bisphosphate aldolase monomer
*elaB*	b2266	*elaB*	1	0.74	0.82	1.04	1	1.35	2.1	1.09	1	1.46	1.34	0.76	a	hypothetical protein
*talA*	b2464	*talA*	1	1.33	1.24	1.55	1	1.67	2.31	1.49	1	2.03	1.62	0.56	a, b	transaldolase A
*tktB*	b2465	*tktB*	1	1.81	0.79	1.65	1	1.26	2.02	1.72	1	1.56	1.51	0.52	a, b	transketolase 2 isozyme
*ygaU*	b2665	*ygaU*	1	1	1.15	1.37	1	1.72	2.45	1.18	1	1.47	1.5	0.49	a, b	hypothetical protein
*ygaM*	b2672	*ygaM*	1	0.48	1.04	1.02	1	2.21	2.24	1.26	1	1.37	1.25	0.49	a	hypothetical protein
*yqjC*	b3097	*yqjCDEK*	1	0.71	1.08	1	1	1.37	1.72	1.22	1	1.55	1.49	0.85	a	hypothetical protein
*yqjD*	b3098	*yqjCDEK*	1	0.69	1.05	1.02	1	1.63	2.13	1.37	1	1.49	1.4	0.87	a	hypothetical protein
*yqjE*	b3099	*yqjCDEK*	1	0.77	1.16	0.97	1	1.27	1.63	0.79	1	1.15	1.47	0.75	a	Hypothetical protein
*yrbL*	b3207	*yrbL*	1	1.98	1.01	2.99	1	2.12	2.62	1.99	1	1.47	1.46	0.86	a	hypothetical protein
*yhiM*	b3491	*yhiM*	1	3.03	1.24	4.03	1	0.69	1.71	0.11	1	1.63	2.38	0.36	g	conserved inner membrane protein
*slp*	b3506	*slp-dctR*	1	0.24	1.08	0.88	1	1.42	8.6	0.64	1	2.22	2.44	0.54	d, e, g	outer membrane protein induced after carbon starvation; starvation lipoprotein
*dctR*	b3507	*slp-dctR*	1	0.24	1.16	1.16	1	0.98	2.34	0.44	1	2.28	2.39	0.87	d, e, g	protein involved in metabolism of C4-dicarboxylates
*yhiD*	b3508	*yhiD*	1	1.12	2.02	3.04	1	1.21	5.16	1.01	1	1.68	2.72	0.61	d, e, g	putative transport ATPase
*hdeB*	b3509	*hdeAB*	1	0.31	0.9	0.81	1	2.32	14.01	0.18	1	2.65	2.28	0.94	d, e, g	hypothetical protein
*hdeA*	b3510	*hdeAB*	1	0.33	0.83	0.66	1	1.76	9.13	0.15	1	2.81	2.07	0.96	d, e, g	hypothetical protein
*hdeD*	b3511	*hdeD*	1	0.36	0.82	1.39	1	1.47	10.3	0.52	1	2.27	2.6	0.6	d, e, f, g	protein involved in acid resistance
*gadE*	b3512	*gadE-mdtEF*	1	0.05	0.52	0.48	1	1.49	12.56	0.26	1	4.07	5.78	1.28	d, e, g	GadE transcriptional activator
*gadW*	b3515	*gadW*	1	0.71	0.58	1.36	1	0.96	1.76	0.91	1	1.79	2.45	1.34	g, FR	putative ARAC-type regulatory protein
*gadX*	b3516	*gadAX*	1	0.33	0.6	0.98	1	1.71	1.92	1.68	1	3.15	4.25	1.66	a, f, FR	GadX transcriptional activator
*gadA*	b3517	*gadAX*	1	0.37	0.2	2.88	1	1.03	15.86	2	1	3.58	4.82	1.25	FR, c	glutamate decarboxylase isozyme
*aceB*	b4014	*aceBAK*	1	29.79	0.58	8.51	1	0.54	0.69	4.48	1	1.8	1.02	1.11	a, d, e, g	malate synthase A
*aceA*	b4015	*aceBAK*	1	6.62	1.31	2.69	1	0.34	0.33	1.26	1	1.44	0.94	0.99	a	isocitrate lyase
*aceK*	b4016	*aceBAK*	1	1.55	1.04	1.19	1	1.89	1.3	1.31	1	1.64	0.92	0.97	c	isocitrate dehydrogenase kinase/phosphatase
*yjbJ*	b4045	*yjbJ*	1	0.74	1.1	1.67	1	1.94	1.32	1.73	1	1.52	1.06	0.47	a, b	hypothetical protein
*ytfK*	b4217	*ytfK*	1	0.36	0.81	0.93	1	0.62	0.66	0.36	1	1.45	1.84	0.46	a	hypothetical protein
*osmY*	b4376	*osmY*	1	1.1	1.41	2.55	1	0.6	0.67	1.78	1	1.93	1.54	0.68	a, b	hyperosmotically inducible periplasmic protein
*ycaC*	b0897	*ycaC*	1	0.68	1.67	0.73	1	1.98	1.64	0.75	1	1.4	1.59	0.59	h, FR	hypothetical protein
*cfa*	b1661	*cfa*	1	0.48	0.95	0.3	1	1.17	2.37	0.2	1	2.19	2.61	0.66	h, FR	cyclopropane fatty acyl phospholipid synthase
*yjiD*	b4326	*yjiD*	1	0.57	1.54	2.41	1	2.03	1.35	8	1	7.42	6.38	1.23	FA	hypothetical protein

1 Regulation of genes known to be induced by an increase in osmolarity: (a) genes described in [53], (b) genes described in [52], (c) gene belongs to an operon known to be regulated by osmotic stress; genes known to respond to acid stress: (d) EvgA overexpression, (e) YdeO overexpression, (f) GadX overexpression, (g) *gadX* mutant [56], (h) genes responding to acid stress independently of GadX and genes controlled by FNR: (FR) FNR-repressed and (FA) FNR-activated [63].

### HU regulates acid-stress genes (Cluster 2)

**Figure 4:**
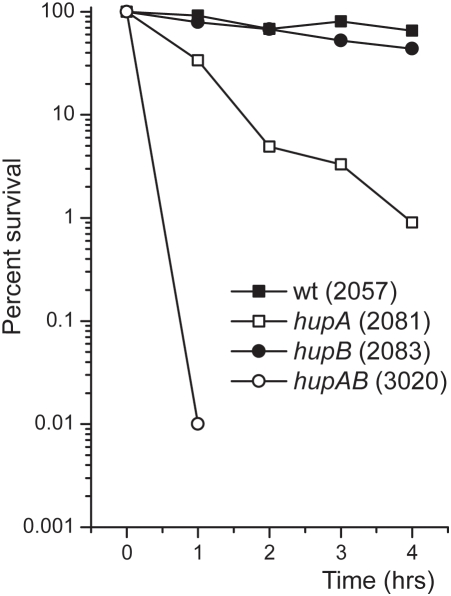
Acid stress Test. The comparative survival of wild type (JO2057), *hupA* (JO2081), *hupB* (JO2083) and *hupAB* (JO3020) strains submitted to acid stress was measured as follows. Samples were taken at different times form cells resuspended in LB medium at pH 2.5 or in saline buffer at pH 7.2, serially diluted and plated on LB agar plates for colony counting. The time points correspond to percent survival of acid-treated cells versus control cells, averaged over two independent experiments.

Cluster 2 also included a number of genes identified as acid inducible in the gene databases. *E. coli* can withstand a pH of 2.5 for several hours. The acid stress response in *E. coli* and related organisms is quite complex and involves a number of regulatory mechanisms [54]. Three or potentially four acid-resistance systems (AR) have been reported [55]. The mechanism involved in the genetic regulation of AR2, which has been extensively investigated, requires only three genes and eleven regulatory proteins. The regulon of two of these (GadX-GadW) has been identified and comprises 15 genes: *gadAXW*, *gadBC*, *ybaST*, *slp-yhi*F, *hdeAB-yhiD*, *yhiM*, *hdeD* and *yhiF* [54]. The normalized expression values shown in Table 3 indicate that 13 of these 15 genes belong to cluster 2 of the HU regulon. In addition, Table 3 shows a compilation of acid-inducible genes in four genetic backgrounds (*gadX* mutant and overexpression of transcriptional regulators EvgA, YdeO and GadX) as reviewed by Foster [56]. A very strong correlation was found between GadX-repressed genes and genes induced in the single *hupA* or *hupB* mutants in stationary phase. Cluster 2 also contained genes that respond to acid stress but are not regulated by GadX. These included *wrbA* (NAD(P)H∶quinone oxidoreductase), *nhaA* (sodium/proton NhaA transporter), *cbpA* (a potential chaperone), *cfa* (cyclopropane fatty acyl phospholipid synthase), *ycaC* and *yebV* (unknown). In the view of these results, we conducted an acid resistance assay on wild-type, *hupA*, *hupB* and *hupAB* strains as described by Masuda and Church [57]. The wild-type and *hupB* strains survived up to 3 hr at low pH; the *hupAB* mutant showed hypersensitivity to acid, and the *hupA* mutant displayed an intermediate phenotype (Fig. 4).

### HU is a novel aerobic regulator of energy metabolism (Clusters 2, 4, 5 & 7)

The prominent part of the genes characterizing the HU regulon is known to be involved in bacterial energy metabolism. They amount to 45% of the total number of the regulated operons and are found in four different clusters: 2, 4, 5 and 7 indicating that they obey different expression patterns.

In the facultative anaerobe *E. coli*, the presence of oxygen and other electron acceptors influences the choice of catabolic and anabolic pathways. *E. coli* prefers to grow using aerobic respiration, but it can achieve anaerobic respiration with nitrate or other electron acceptors when oxygen is absent; fermentation is used as a last resort when no electron acceptor is available. The expression of enzymes involved in energy metabolism is regulated mainly at the transcriptional level. Two separate oxygen sensing/transcriptional regulatory mechanisms are essential for the aerobic/anaerobic switch (for a review see [58]). First, a two-component system, responsible for micro-aerobic metabolism regulation, is composed of a membrane-bound histidine sensor kinase (ArcB) able to phosphorylate a transcriptional regulator (ArcA) [59]. The *E. coli* ArcAB regulon comprises 175 genes involved in a large number of cell processes [60]. FNR is the second transcriptional sensor-regulator protein involved in the control of anaerobic metabolism; it acts either as a transcription repressor or as an activator. The Fe–S-containing FNR protein is capable of oxygen-regulated dimerization and DNA binding [61]. The *E. coli* FNR regulon was initially investigated by several laboratories but with somewhat divergent results probably due to different genetic backgrounds and growth conditions [60], [62]. More recently, a report based on a more extensive study has re-evaluated the extent of the FNR regulon [63].

The absence of one or both HU subunits deregulated transcription of the vast majority of the genes encoding electron donors/acceptors or involved in fermentation and in aerobic/anaerobic respiration. A direct comparison of data presented in Clusters 2, 4, 5 and 7 to energy metabolism regulons showed a striking resemblance between the HU and FNR regulons. More surprisingly, the directionality of the HU-FNR regulation was well conserved with a very few exceptions: all the genes induced (or repressed) anaerobically by FNR were also induced (or repressed) by HU in the presence of oxygen. Clusters 2 and 5 contained a majority of FNR down-regulated genes whereas clusters 4 and 7 were populated with a majority of FNR up-regulated genes (See Tables 3, 4, 5 and 6 for the relative, normalized gene expression values). Effectively, HU binding to the FNR regulated, *ndh* promoter has been reported [64].

**Table 4 pone-0004367-t004:** Cluster 4 genes.

Gene	Blattner	Operon	Exponential				Transition				Stationary				Regulation^1^	Function
			WT	*hupA*	*hupB*	*hupAB*	WT	*hupA*	*hupB*	*hupAB*	WT	*hupA*	*hupB*	*hupAB*		
*dcuC*	b0621	*dcuC*	1	0.03	1.22	0.08	1	0.8	0.87	0.15	1	1.03	1.04	0.96	FA, FAec	transport of dicarboxylates
*dmsA*	b0894	*dmsABC*	1	0.06	1.82	0.14	1	1.79	1.78	0.14	1	1.04	0.61	1.03	FA, FAec	anaerobic dimethyl sulfoxide reductase subunit A
*dmsB*	b0895	*dmsABC*	1	0.05	1.59	0.13	1	2.16	2.19	0.3	1	1.5	1.48	1.65	FA, FAec	anaerobic dimethyl sulfoxide reductase subunit B
*narK*	b1223	*narK*	1	0.02	1.08	0.16	1	3.44	4.07	7.28	1	1.68	1.02	1.26	FA, FAec	nitrite extrusion protein
*narG*	b1224	*narGHJI*	1	0.02	1.65	0.12	1	4.86	4.82	3.78	1	1.32	1.99	2.3	FA, FAec	nitrate reductase 1, alpha subunit
*narH*	b1225	*narGHJI*	1	0.06	1.64	0.13	1	2.85	3.71	2.09	1	1.13	0.77	1.75	FA, FAec	nitrate reductase 1, beta subunit
*narJ*	b1226	*narGHJI*	1	0.06	1.31	0.13	1	1.89	1.68	1.57	1	1.14	1.06	1.36	FA, FAec	nitrate reductase 1, delta subunit, assembly function
*narI*	b1227	*narGHJI*	1	0.09	1.53	0.17	1	1.84	1.72	1.63	1	1.66	1.09	1.27	FA, FAec	nitrate reductase 1, cytochrome b(NR), gamma subunit
*adhE*	b1241	*adhE*	1	0.12	1.17	0.15	1	0.88	0.95	0.22	1	0.78	0.97	0.4	FAec	CoA-linked acetaldehyde dehydrogenase and iron-dependent alcohol dehydrogenase pyruvate-formate-lyase deactivase
*napB*	b2203	*napFDAGHBC-ccmABCDEFGH*	1	0.23	2.15	0.24	1	10.94	8.68	25.5	1	2.95	1.93	1.67	FA, FAec	cytochrome c-type protein
*napD*	b2207	*napFDAGHBC-ccmABCDEFGH*	1	0.09	0.72	0.22	1	1.86	1.76	6.71	1	0.84	0.72	0.88	FA, FAec	hypothetical protein
*napF*	b2208	*napFDAGHBC-ccmABCDEFGH*	1	0.04	0.66	0.22	1	1.43	1.56	7.67	1	1	2.55	2.21	FA, FAec	ferredoxin-type protein: electron transfer
*nirD*	b3366	*nirBDC-cysG*	1	0	1.54	0.05	1	1.35	0.39	0.72	1	2.34	3.43	1.59	FA	nitrite reductase (NAD(P)H) subunit
*nirC*	b3367	*nirBDC-cysG*	1	0.02	1.91	0.09	1	1.17	1.05	1.79	1	1.31	1.15	0.8	FA	nitrite reductase activity
*feoB*	b3409	*feoAB*	1	0.12	0.67	0.77	1	0.16	0.41	0.69	1	0.76	1.13	0.93	FA, FAec	ferrous iron transport protein B
*cspA*	b3556	*cspA*	1	0.22	1.03	0.73	1	0.85	0.9	0.6	1	0.5	0.39	1.27	FR	cold shock protein 7.4, transcriptional activator of hns
*nrfA*	b4070	*nrfABCDEFG*	1	0.14	1.66	0.16	1	2.25	0.82	1.41	1	1.54	1.21	1.16	FA, FAec	periplasmic cytochrome c(552): plays a role in nitrite reduction
*nrfB*	b4071	*nrfABCDEFG*	1	0.09	2.16	0.07	1	4.15	1.19	1.51	1	4.71	3.62	2.6	FA, FAec	formate-dependent nitrite reductase a penta-haeme cytochrome c
*nrfC*	b4072	*nrfABCDEFG*	1	0.14	2.19	0.15	1	2.8	1.3	0.84	1	1.76	1.62	0.69	FA, FAec	formate-dependent nitrite reductase Fe-S centers
*yjdK*	b4128	*yjdKO*	1	0.03	1.63	0.32	1	1.06	1.65	0.92	1	1.82	1.15	0.81	FA	hypothetical protein
*yjjW*	b4379	*yjjW*	1	0.05	1.51	0.31	1	2.21	1.6	0.11	1	4.19	0.57	0.53	FA	putative activating enzyme
*yjjI*	b4380	*yjjI*	1	0.03	1.39	0.27	1	1.88	1.5	0.31	1	1.58	1.45	0.94	FA	hypothetical protein

1 Regulation symbols: FA: FNR-activated and FR: FNR-repressed [63]; FAec: FNR-activated (Ecocyc: http://ecocyc.org).

**Table 5 pone-0004367-t005:** Cluster 5 genes.

Gene	Blattner	Operon	Exponential				Transition				Stationary				Regulation^1^	Function
			WT	*hupA*	*hupB*	*hupAB*	WT	*hupA*	*hupB*	*hupAB*	WT	*hupA*	*hupB*	*hupAB*		
*cyoD*	b0429	*cyoABCDE*	1	2.14	0.73	1.63	1	0.03	0.04	0.73	1	0.34	0.59	0.58	FR, FRec	cytochrome o ubiquinol oxidase subunit IV
*cyoC*	b0430	*cyoABCDE*	1	2.23	0.81	1.56	1	0.07	0.06	0.75	1	0.51	0.53	0.64	FR, FRec	cytochrome o ubiquinol oxidase subunit III
*cyoB*	b0431	*cyoABCDE*	1	2.25	0.84	1.67	1	0.07	0.06	1.02	1	0.43	0.43	0.73	FR, FRec	cytochrome o ubiquinol oxidase subunit I
*cyoA*	b0432	*cyoABCDE*	1	2.71	0.84	2.37	1	0.18	0.1	1.52	1	0.47	0.53	0.75	FR, FRec	cytochrome o ubiquinol oxidase subunit II
*sdhC*	b0721	*sdhCDAB-b0725-sucABCD*	1	13.94	0.99	7.68	1	0.29	0.17	1.57	1	0.36	1.01	1.02	FRec	succinate dehydrogenase, cytochrome b556
*sdhD*	b0722	*sdhCDAB-b0725-sucABCD*	1	9.23	0.79	5.2	1	0.23	0.15	1.7	1	0.42	0.71	0.79	FRec	succinate dehydrogenase, hydrophobic subunit
*sdhA*	b0723	*sdhCDAB-b0725-sucABCD*	1	7.2	0.82	4.21	1	0.15	0.16	1.08	1	0.29	0.55	0.63	FRec	succinate dehydrogenase, flavoprotein subunit
*sdhB*	b0724	*sdhCDAB-b0725-sucABCD*	1	7.53	0.76	3.67	1	0.14	0.16	1.08	1	0.32	0.63	0.53	FRec	succinate dehydrogenase, iron sulfur protein
*b0725*	b0725	*sdhCDAB-b0725-sucABCD*	1	4.65	0.66	2.64	1	0.18	0.22	0.72	1	0.38	0.88	0.49	FRec	hypothetical protein
*sucA*	b0726	*sdhCDAB-b0725-sucABCD*	1	2.71	0.7	2.28	1	0.15	0.22	0.74	1	0.26	0.56	0.45	FRec	2-oxoglutarate dehydrogenase (decarboxylase component)
*sucB*	b0727	*sdhCDAB-b0725-sucABCD*	1	1.83	0.67	1.79	1	0.15	0.2	0.7	1	0.29	0.6	0.62	FRec	2-oxoglutarate dehydrogenase (dihydrolipoyltranssuccinase E2 component)
*sucC*	b0728	*sdhCDAB-b0725-sucABCD*	1	1.81	0.63	1.97	1	0.16	0.19	0.84	1	0.33	0.72	0.66	FRec	succinyl-CoA synthetase, beta subunit
*sucD*	b0729	*sdhCDAB-b0725-sucABCD*	1	2.26	0.69	1.95	1	0.14	0.14	0.62	1	0.42	0.74	0.49	FRec	succinyl-CoA synthetase, alpha subunit
*fumA*	b1612	*fumA*	1	5.47	0.82	3.05	1	0.34	0.23	1.13	1	0.49	0.47	1.55	FRec	fumarase A = fumarate hydratase Class I aerobic isozyme
*fliY*	b1920	*fliAZY*	1	1.85	0.69	1.85	1	1.11	0.91	3.26	1	1.42	1.01	0.73	FA	putative periplasmic binding transport protein
*ndk*	b2518	*ndk*	1	16.55	0.94	12.98	1	0.67	0.3	8.27	1	0.8	0.98	5.24	This work	nucleoside diphosphate kinase
*lldP*	b3603	*lldPRD*	1	49.62	0.9	17.04	1	0.86	0.54	2.38	1	0.58	0.53	0.65	This work	L-lactate permease
*lldR*	b3604	*lldPRD*	1	29.89	0.79	15.07	1	0.07	0.13	0.33	1	0.73	0.54	0.86	This work	transcriptional regulator
*lldD*	b3605	*lldPRD*	1	12.6	0.79	7.63	1	0.21	0.15	0.56	1	0.68	0.58	0.82	This work	L-lactate dehydrogenase
*fimA*	b4314	*fimAICDFGH*	1	0.34	0.81	2.8	1	0.22	0.63	2.56	1	0.49	0.43	3.68	FA	major type 1 subunit fimbrin (pilin)
*fimI*	b4315	*fimAICDFGH*	1	0.45	1.28	5.45	1	0.58	0.88	5.27	1	1.28	1.29	5.09	FA	fimbrial protein
*fimC*	b4316	*fimAICDFGH*	1	0.5	1.09	6.36	1	0.74	1.04	8.35	1	1.32	1.17	3.75	FA	periplasmic chaperone, required for type 1 fimbriae

1 Regulation symbols: FA: FNR-activated and FR: FNR-repressed [63]; FRec: FNR-repressed (Ecocyc: http://ecocyc.org).

**Table 6:** Cluster 7 genes.

Gene	Blattner	Operon	Exponential				Transition				Stationary				Regulation^1^	Function
			WT	*hupA*	*hupB*	*hupAB*	WT	*hupA*	*hupB*	*hupAB*	WT	*hupA*	*hupB*	*hupAB*		
*aceE*	b0114	*pdhR-aceEF-lpd*	1	0.44	1.16	0.71	1	1.37	1.51	0.35	1	0.78	0.75	1.02	FR, FRec	pyruvate dehydrogenase (decarboxylase component)
*aceF*	b0115	*pdhR-aceEF-lpd*	1	0.46	1.35	0.65	1	1.51	1.58	0.41	1	0.74	0.73	1.04	FR, FRec	pyruvate dehydrogenase (dihydrolipoyltransacetylase component)
*ybcW*	b0559	*ybcW*	1	0.27	0.97	1.46	1	0.89	1.94	0.29	1	4.07	4.27	0.72	FA	hypothetical protein
*cydA*	b0733	*cydAB*	1	0.16	1.17	0.26	1	1.46	1.62	0.15	1	0.57	0.57	0.64	FR, FRec	cytochrome d terminal oxidase, polypeptide subunit I
*cydB*	b0734	*cydAB*	1	0.17	1.1	0.28	1	1.74	2	0.17	1	0.53	0.61	0.7	FR, FRec	cytochrome d terminal oxidase polypeptide subunit II
*pflB*	b0903	*focA-pflB*	1	0.19	1.13	0.24	1	3.01	3.22	0.25	1	0.61	0.85	0.39	FA, FAec	formate acetyltransferase 1
*ycbJ*	b0919	*ycbJ*	1	0.05	1.23	0.16	1	0.72	0.96	0.37	1	0.89	0.93	0.7	FA	hypothetical protein
*ndh*	b1109	*ndh*	1	0.05	0.81	0.54	1	0.85	1.9	0.34	1	1.44	0.88	0.32	FR, FRec	respiratory NADH dehydrogenase
*ompW*	b1256	*ompW*	1	0.19	1.32	0.29	1	1.66	1.42	0.34	1	0.43	0.41	2.29	FA, FAec	putative outer membrane protein
*fdnG*	b1474	*fdnGHI*	1	0.12	1.39	0.36	1	6.69	3.57	2.55	1	1.06	0.91	0.98	FA, FAec	formate dehydrogenase-N, nitrate-inducible, alpha subunit
*fdnI*	b1476	*fdnGHI*	1	0.14	1.32	0.26	1	9.21	4.34	2.48	1	1.22	1.56	1.17	FA, FAec	formate dehydrogenase-N, nitrate-inducible, cytochrome B556(Fdn) gamma subunit
*ydfZ*	b1541	*ydfZ*	1	0.17	0.96	0.39	1	3.97	3.66	1.14	1	1.22	1.23	1.39	FA	hypothetical protein
*ynfE*	b1587	*ynfEFGH-dmsD*	1	0.18	1.73	0.26	1	2.05	2.19	0.09	1	1.66	1.19	1.37	FA, FAec	putative oxidoreductase, major subunit
*ynfF*	b1588	*ynfEFGH-dmsD*	1	0.13	2.24	0.12	1	4.96	4.83	0.14	1	0.32	0.3	2.08	FA, FAec	putative oxidoreductase, major subunit
*ynfG*	b1589	*ynfEFGH-dmsD*	1	0.26	2.05	0.14	1	7.12	4.24	0.12	1	0.21	1.67	1.99	FA, FAec	putative oxidoreductase, Fe-S subunit
*ynfH*	b1590	*ynfEFGH-dmsD*	1	0.64	1.48	0.87	1	5.19	3.77	0.67	1	1.56	1.17	1.13	FA, FAec	putative DMSO reductase anchor subunit
*ydhY*	b1674	*ydhYVW*	1	0.3	1.34	0.74	1	0.77	1.99	0.36	1	1.96	0.88	1.37	FA	putative oxidoreductase, Fe-S subunit
*yeaU*	b1800	*yeaU*	1	0.34	0.41	2.67	1	0.15	0.44	0.02	1	0.62	0.9	0.3	FR	putative tartrate dehydrogenase
*glpA*	b2241	*glpABC*	1	0.4	1.24	0.28	1	1.02	0.89	0.35	1	0.35	0.36	0.55	FA, FAec	sn-glycerol-3-phosphate dehydrogenase (anaerobic), large subunit
*glpB*	b2242	*glpABC*	1	0.44	1.12	0.23	1	1.16	0.96	0.24	1	0.28	0.29	0.54	FA, FAec	sn-glycerol-3-phosphate dehydrogenase (anaerobic), membrane anchor subunit
*glpC*	b2243	*glpABC*	1	0.54	1.24	0.31	1	1.2	1.17	0.27	1	0.26	0.33	0.51	FA, FAec	sn-glycerol-3-phosphate dehydrogenase (anaerobic), K-small subunit
*uraA*	b2497	*upp-uraA*	1	0.42	1.03	0.49	1	0.66	0.07	0.8	1	0.93	1.11	0.95	FA, FAec	uracil transport
*upp*	b2498	*upp-uraA*	1	0.37	0.83	0.53	1	0.76	0.39	0.79	1	0.85	0.91	1.18	FA, FAec	uracil phosphoribosyltransferase
*yfiD*	b2579	*yfiD*	1	0.04	0.95	0.41	1	2.29	2.13	0.54	1	0.4	0.31	0.98	FA, FAec	putative formate acetyltransferase
*gcvH*	b2904	*gcvTHP*	1	0.92	0.96	0.55	1	1.08	1	0.59	1	0.21	0.38	0.24	FAec	in glycine cleavage complex, carrier of amino-methyl moiety via covalently bound lipoyl cofactor
*ansB*	b2957	*ansB*	1	0.01	1.55	0.18	1	2.87	1.38	0.23	1	0.7	1.3	1.51	FA, FAec	periplasmic L-asparaginase II
*uxaA*	b3091	*uxaCA*	1	2.64	1.01	0.63	1	1.46	0.56	0.11	1	0.51	0.47	0.6	FA, FAec	altronate hydrolase
*uxaC*	b3092	*uxaCA*	1	1.17	0.84	0.59	1	2.31	0.64	0.11	1	0.78	0.67	1.06	FA, FAec	uronate isomerase
*tdcF*	b3113	*tdcABCDEFG*	1	0.87	1.15	0.44	1	16.22	14.14	0.51	1	1.29	1.12	1.21	FA, FAec	hypothetical protein
*tdcE*	b3114	*tdcABCDEFG*	1	0.68	1.11	0.29	1	18.25	15.59	0.35	1	1.29	1.27	1.25	FA, FAec	probable formate acetyltransferase 3
*tdcD*	b3115	*tdcABCDEFG*	1	0.51	1.94	0.33	1	4.3	3.86	0.08	1	1.38	1.14	1.2	FA, FAec	putative kinase
*tdcC*	b3116	*tdcABCDEFG*	1	0.35	1.67	0.25	1	2.3	2.12	0.04	1	0.8	0.76	0.77	FA, FAec	anaerobically inducible L-threonine, L-serine permease
*tdcB*	b3117	*tdcABCDEFG*	1	0.12	1.24	0.16	1	1.09	1.07	0.04	1	0.78	0.51	1.68	FA, FAec	threonine dehydratase, catabolic
*tdcA*	b3118	*tdcABCDEFG*	1	0.01	1.05	0.25	1	1.2	1.01	0.4	1	0.75	1.32	1.28	FA, FAec	transcriptional activator of tdc operon
*malP*	b3417	*malPQ*	1	0.53	1.55	0.94	1	1.71	1.58	0.28	1	1	0.88	1.27	FA, FAec	maltodextrin phosphorylase
*katG*	b3942	*katG*	1	1	1.87	0.96	1	3.05	1.71	0.63	1	0.51	0.64	1.06	FA, FAec	catalase hydroperoxidase HPI(I)
*fumB*	b4122	*dcuB-fumB*	1	0.22	1.56	0.28	1	7.16	11.83	0.12	1	1.71	1.52	1.07	FA, FAec	fumarase B = fumarate hydratase Class I anaerobic isozyme
*dcuB*	b4123	*dcuB-fumB*	1	0.04	1.21	0.21	1	5.24	10.38	0.49	1	2.34	1.58	0.76	FA, FAec	anaerobic dicarboxylate transport
*dcuA*	b4138	*aspA-dcuA*	1	0.32	1.5	0.41	1	1.27	0.76	0.3	1	0.84	0.67	0.87	FA, FAec	anaerobic dicarboxylate transport
*frdD*	b4151	*frdABCD*	1	0.48	1.31	0.33	1	2.39	1.99	0.23	1	0.79	0.94	1.07	FA, FAec	fumarate reductase, anaerobic, membrane anchor polypeptide
*frdC*	b4152	*frdABCD*	1	0.48	1.4	0.3	1	1.93	1.67	0.11	1	0.53	0.65	1.16	FA, FAec	fumarate reductase, anaerobic, membrane anchor polypeptide
*frdB*	b4153	*frdABCD*	1	0.44	1.4	0.31	1	1.91	1.61	0.15	1	1.03	0.85	1.19	FA, FAec	fumarate reductase, anaerobic, iron-sulfur protein subunit
*frdA*	b4154	*frdABCD*	1	0.4	1.27	0.33	1	1.7	1.46	0.18	1	0.77	0.81	1.09	FA, FAec	fumarate reductase, anaerobic, flavoprotein subunit
*tdcG*	b4471	*tdcABCDEFG*	1	0.82	1.22	0.58	1	10.16	7.41	0.94	1	1.69	1	0.97	FA, FAec	L-serine deaminase 3

1 Regulation symbols: FA: FNR-activated and FR: FNR-repressed [63]; FRec: FNR-repressed and FAec: FNR-ativated (Ecocyc: http://ecocyc.org).

**Figure 5:**
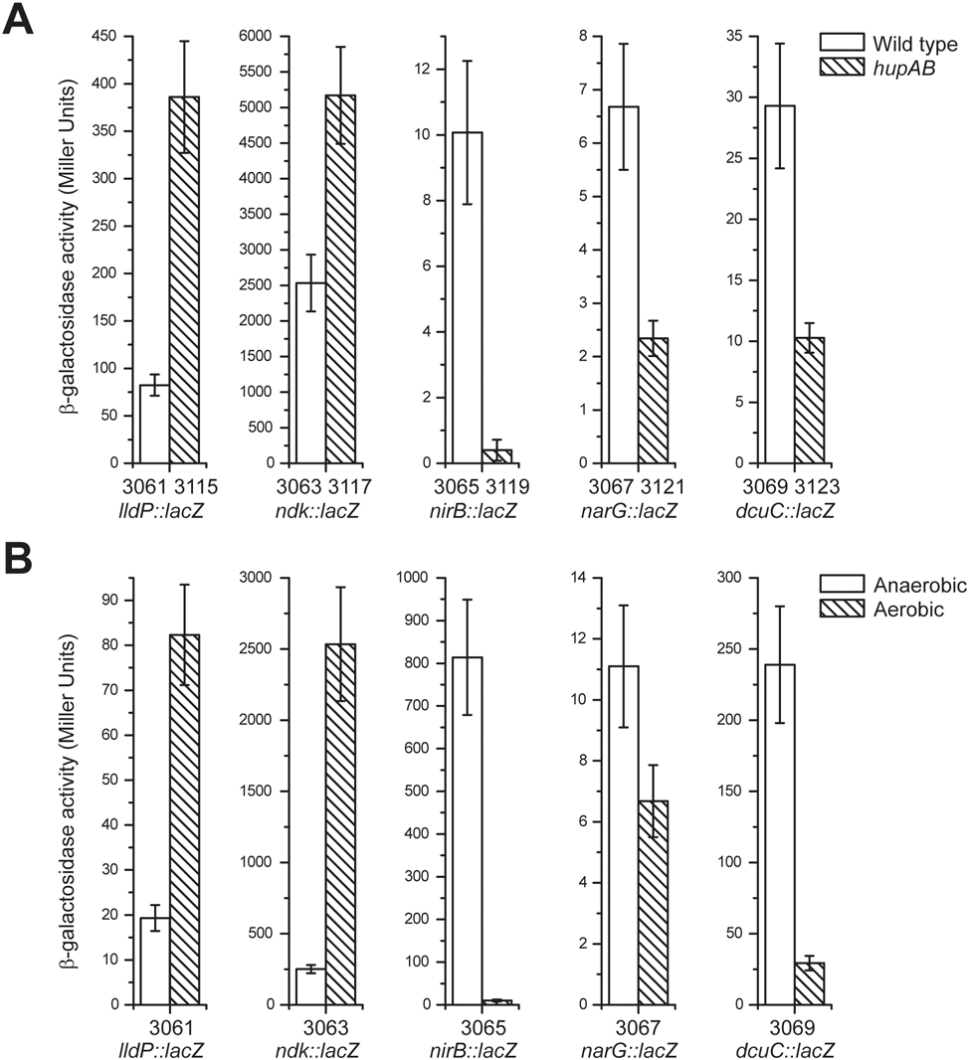
Regulation of cluster 4 and 5 genes by HU and aerobiosis. (A) Comparison of the beta-galactosidase activity of *lldp*, *ndk*, *nirB*, *narG* and *dcuC* transcriptional *lacZ* fusions in wild type and *hupAB* strains. (B) Comparison of the beta-galactosidase activity of the same gene fusions in aerobic and anaerobic conditions. The numbers under the bars correspond to strain numbers described in Table 7.

**Figure 6:**
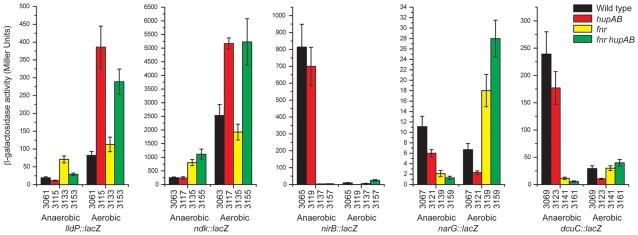
Regulation of cluster 4 and 5 genes by HU, aerobiosis and FNR. Individual and combined effects of the wild type, *Δfnr*, *hupAB* and *Δfnr hupAB* backgrounds and aerobic or anaerobic growth conditions on the beta-galactosidase activity of *lldp*, *ndk*, *nirB*, *narG* and *dcuC* transcriptional *lacZ* fusions. The numbers under the bars correspond to strain numbers described in Table 7.

In order to investigate and compare the *in vivo* regulatory relationships between HU and the aerobiosis/anaerobiosis system, we constructed single copy *lacZ* transcriptional fusions to several genes strongly activated or repressed by HU as described above. We chose three HU-activated genes from cluster 4, namely *nirB*, *narG* and *dcuC*, encoding respectively the large subunit of nitrite reductase, the α subunit of nitrate reductase and the anaerobic C4-dicarboxylate transporter. These genes are known to be positively regulated by FNR [63]. In parallel, two HU-repressed genes from cluster 5, *lldP* and *ndk*, encoding respectively the L-lactate permease and the nucleoside diphosphate kinase, were selected on the basis of their strong response in the transcriptome analysis. The transcription of the *lldPRD* operon, as seen in Figure 5, is repressed anaerobically by ArcA-P [65]. The expression of *ndk* is negatively controlled in anoxic conditions by an unknown mechanism [62]. In good agreement with microarrays experiments, we observed *in vivo* that heterodimeric HUαβ aerobically repressed *lldP* and *ndk* and stimulated *nirB*, *narG* and *dcuC* (Fig. 5A). This is what we observed in anoxic conditions: we confirmed *lldP* and *ndk* repression and the induction of *nirB*, *narG* and *dcuC* (Fig. 5B). The expression of β-galactosidase by these five gene fusions was then tested in four genetic backgrounds (wild-type, *fnr*, *hupAB* and *fnr hupAB*) and in aerobic or anaerobic conditions (Fig. 6). Several observations could be made: (i) the regulatory effect of HU was only apparent in oxic conditions and wss stronger for genes that are normally repressed in anaerobiosis, such as *lldP* and *ndk*; (ii) in aerobiosis, there was no significant difference between *fnr^+^* and *fnr^−^* strains with the exception a two-fold effect for *narG* and (iii) in anaerobiosis, we did not observe a significant difference between the HU^+^ and HU^−^ derivatives of the five gene fusion strains: the only measurable effect was due to the presence/absence of FNR, especially for the FNR-regulated genes *nirB*, *narG* and *dcuC*.

**Figure 7:**
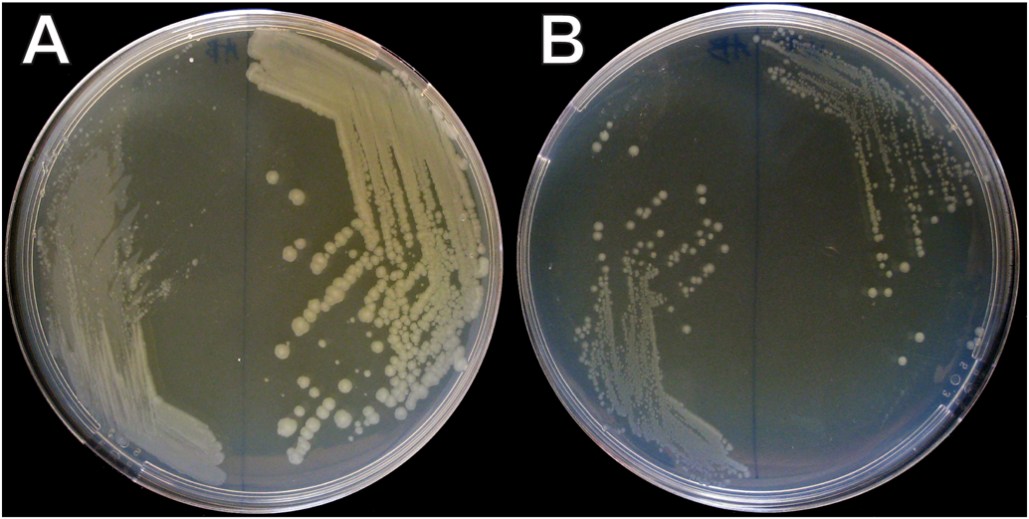
Phenotype of a HU^−^ strain in the presence and absence of oxygen. (A) Colony phenotype of the *hupAB* (JO3020, left) and wild type (JO2057, right) strains in aerobic conditions. (B) Colony phenotype of the same strains in anaerobic conditions. The strains are plated on LB agar supplemented for anaerobic growth (see Material and Methods).

These results prompted us to test the growth phenotype of a *hupAB* strain in the absence of oxygen on complete anoxic medium (see Materials and Methods). Surprisingly, we noted that the very slow growth phenotype caused by the absence of HU in aerobic conditions was not observed anaerobically. In anoxic conditions, the HU-deficient strain lost its very small colony phenotype and displayed a similar growth rate as the wild type control strain (Fig. 7). After a number of verifications, we concluded that HU was not necessary for growth in the absence of oxygen.

## Discussion

### Identification of the HU regulon

We examined the effect of HU, one of the most abundant nucleoid-associated proteins in the bacterial cell, on genome-wide transcription. Since HU exists as three dimeric forms in *E. coli* (HUαβ, HUα2 and HUβ2), depending mainly on the growth phase, the respective role of each dimer was analyzed. We compared the expression pattern of all *E. coli* genes in the wild-type host and in strains carrying a mutation in one or in both HU-encoding genes: the *hupA*, *hupB* and *hupAB* mutants. In each case, the cultures were sampled at three different growth phases for two reasons: we had shown that the expression of the HU genes is regulated by growth phase [8] and that the expression of the stationary phase sigma factor, RpoS is stimulated by HU at the translational level [17]. An unsupervised statistical clustering analysis allowed to subtract the interference from growth phase and RpoS and to identify the *E. coli* genes strictly controlled by HU at the transcriptional level. After this correction, the analysis showed that the transcription of a total of 353 genes composing 229 operons is affected by the lack of one or both HU subunits. The accuracy of the data and its processing was well supported by the number of observed polycistronic operons where all the genes are co-regulated (Supplemental Table S4).

The five HU-regulated clusters identified are populated with genes involved in aerobic/anaerobic energy metabolism and to a lesser extent in the SOS response, osmolarity stress response, and acid stress response. We were able to discriminate between three distinct HU regulons: the HUαβ regulon (cluster 6 repressed by HUαβ and cluster 7 induced by HUαβ); the HUα2 or HUαβ regulons (cluster 4 repressed by HUαβ and cluster 5 repressed by HUα2 in exponential phase) and the HUα2 or HUβ2 regulons (cluster 2, repressed in stationary phase only). Four reasons might explain why we did not observe genes regulated exclusively by HUβ2. First, HUβ2 is unable to introduce negative supercoiling *in vitro* on a relaxed DNA template in the presence of topoisomerase I [5], [8]; second, HUβ2 is normally not present in the cell at 37°C: as soon as it is synthesized it forms the heterodimer [14]; third, thermodynamic studies have shown that HUβ2 is partially denatured at 37°C [16], and fourth, the synthesis of HUβ2 is preferentially stimulated during cold shock [66]. The HUβ2 regulon might therefore be linked to low temperature environments.

The largest HU regulon clusters (2, 4, 5 and 7) share striking similarities with the FNR regulon: genes activated or repressed by FNR in anoxic conditions were respectively activated or repressed by HU in the presence of oxygen. Clearly, FNR and HU exert their regulatory control independently: i) the microarray data showed that FNR expression is not affected in *hup* mutants and ii) the microarray experiments were carried out in aerobic conditions in which FNR is expressed under its apoFNR inactive form [67]. HU could therefore be considered as an aerobic modulator of the FNR regulon.

The transcriptome profiling experiments described in this work showed that a second group of genes, namely the SOS response (or LexA regulon) was induced in the absence of both HU subunits. However, the *in vivo* experiments, presented in Figure 3, showed that SOS induction is much less efficient in a *hupAB* background, as observed previously [20]. From these observations, it was possible to conclude that HU is necessary for tight repression as well as for full derepression of the SOS regulon genes found in cluster 6. The “flattening” of the SOS response in the absence of HU could be explained by the capacity of this protein to displace the LexA repressor from its DNA-binding sites [22].

A third group of genes, namely those composing the acid stress or GadX regulon, was found to belong to the HU regulon as well. We tested whether the induction of these genes, induced in the single *hupA* and *hupB* mutants, would confer acid resistance *in vivo*. The acid resistance assay indicated that low pH strongly affected the survival of the *hupAB* mutant and of the *hupA* mutant to a lower extent. This effect could be explained by the accumulation of protons intra- or extracellularly due to the deregulation of the *cyo* and *cyd* operons encoding cytochrome proton pumps. However, the increase in transcription of acid resistance genes in the *hupA* and *hupB* single mutants observed in cluster 2 was insufficient to permit low pH adaptation (Fig. 4).

The HU regulon comprised also a fourth group of genes known to be induced by osmotic shock. The involvement of HU in the adaptation of cell growth in hyperosmolar environments is well known [24]. We observed an excellent correlation between cluster 2 of the HU regulon and genes involved in the synthesis of osmoprotectants, which respond strongly to the osmotic response via DNA supercoiling [53]. Since HU is able to constrain DNA supercoiling and the regulation of these genes requires modulation of DNA superhelicity, it seemed worthwhile to analyze the HU regulon under this perspective.

### The HU regulon and DNA supercoiling

The relationship of HU with DNA supercoiling has been analyzed in a number of reports. Nucleoid sedimentation experiments have shown that the absence of HU causes a decrease in chromosomal supercoiling [12], [68]. The relaxation activity on supercoiled plasmids of wild type and HU mutants strains increases in the order *hup+*<*hupB*<*hupA*<*hupAB* [11]. A cross-talk between HU and topoisomerase I activity has been observed: the absence of HU generates more unconstrained supercoiling, which in turn requires an increase in relaxing activity in order to maintain physiological levels [11]. Mutations in the *gyrB* gene can compensate the lack of HU [12]. *In vitro* experiments have shown that HUα2, like HUαβ but not HUβ2, constrains DNA superhelicity [5], [8]. Finally, the involvement of HU in DNA supercoiling has been demonstrated at the crystallographic level [69]–[71].

To further investigate the link between HU and DNA superhelicity, we performed a systematic comparison between the HU regulon and the lists of genes under supercoiling control established independently by two groups [72], [73]. We observed that the HU regulon shared very few genes (<8% and <4%, respectively) with the superhelicity regulons (Supplemental Fig. S1 A & Tables S6 to S9). This shared subset of the HU regulon contained genes regulated by supercoiling and osmolarity (*otsB*) or by supercoiling and acid stress (*nhaA* and *gadB*). The same comparison was repeated with the regulons of the two other major nucleoid proteins H-NS and Fis [72] with a very similar outcome (Supplemental Fig. S1 B, C & Tables S10 to S14). We deduced from these observations that the majority of genes under transcriptional superhelical control are regulated by unconstrained chromosomal supercoiling and not by the constraining activity of HU, H-NS and Fis. The regulons of these three proteins were then compared to analyze their respective contribution to global regulation.

### Global regulation by HU, H-NS and Fis

Identification of the HU regulon permitted the systematic comparison with the respective regulons described recently of the other major nucleoid-associated proteins H-NS and Fis, [72]. Taken together, these three abundant proteins are responsible for most of the compaction of the bacterial chromosome: it has been reported that half of the negative supercoiling is constrained by Fis, H-NS and HU [74]. Our data indicated that HU, H-NS and Fis regulons share 15% to 32% of their genes, while specific genes range from 59% to 69%; only 26 genes are common to the three regulons (Supplemental Fig. S1 D & Tables S15 to S18). Several genes are co-regulated by HUαβ and H-NS and encode proteins that repress the acid stress response genes and the biosynthesis of fimbriae, whereas both induce flagellar biosynthetic genes (Supplemental Table S15). A number of chaperone genes and environmental stress response genes are differentially regulated by HU and H-NS (Supplemental Table S15). By comparing the genes co-regulated by HU and Fis, we observed that both proteins induce Cluster 4 genes while they repress Cluster 5 genes (Supplemental Table S16). We observed also that HU regulates these two clusters in the exponential phase, at the stage of growth where Fis is most actively synthesized [14].

### HU-DNA binding and transcription regulation

The identification of a regulon assumes that its regulator interacts with specific genes, upstream of the protein coding sequence. How does HU recognize its targets? The overlap between the FNR and HU regulons suggested that FNR binding-site variants might be recognized by HU. We therefore investigated, by Gibbs sampling, the promoter region of the regulated operons in each cluster. This search failed to produce significant shared sequence motif (data not shown). It is interesting to note that FNR can bind to some of its targets in the absence of a canonical FNR binding sequence, suggesting cooperative binding with another factor [75].

Among the various HU-nucleic acid binding properties that have been described, different DNA binding modes can be invoked to explain mechanistically its regulatory function. Namely, HU contributes to DNA loop formation [26], is capable of constraining supercoiling DNA [69]. and shows higher affinity for distorted DNA structures [23].

The HU regulon is composed of four well defined biological classes of genes involved in stress response and adaptation to environmental shifts. These four classes can be divided into two categories on the basis of the reported DNA binding modes of HU, described above. The regulation of the genes in first category requires, in addition to HU, specific DNA binding of the regulatory proteins LexA, GadX or FNR. We hypothesize that HU induces DNA looping to help loading/unloading of these regulators onto their specific binding sites in order to allow/block RNA polymerase transcription initiation. The formation of such a complex, called “repressosome,” has been studied in detail for the *gal* operon. It involves the participation of the GalR repressor, HU, and negatively supercoiled DNA [26]. A similar phenomenon has been reported for the control of the FNR-regulated *ndh* gene [64].

The second category of the HU regulon contains genes known to be controlled primarily by DNA supercoiling during hyperosmolar upshift without the involvement of a specific regulatory protein [53]. For these genes, it appears that the driving force of regulatory control is solely constituted by the superhelical DNA constraining capability of HU; effectively, these genes are not found in the reported supercoiling regulons [72], [73].

We also observed a good correlation between genes regulated by HU and the chromosomal regions exhibiting “extreme structure” predicted by the group of Ussery: these authors analyzed five parameters affecting the DNA conformation of the *E. coli* chromosome and identified 36 sites presenting a maximal distortion [76]. We found that 15 of these sites mapped in (or very near) genes belonging to the HU regulon (Supplemental Table S19). This observation is consistent with the preferred interaction of HU with distorted DNA structures rather than sequences [15], [19].

### Global regulatory function and structural role of HU

How could we reconcile the transcriptional and chromosomal architectural roles of HU? We have shown here that HU controls the transcription of 353 genes composing 229 operons. Phenotypically, *E. coli hupAB* strains grow very poorly and display numerous enucleated cells. These deleterious effects might be caused by the inverted expression pattern of stress-induced genes and energy metabolism operons and to loss of the nucleoid architecture. Interestingly, these phenotypes are only visible in the presence of oxygen and are rescued under anoxic conditions. The absence of HU regulatory effect in anaerobiosis can be explained by the increase of negative supercoiling, in these conditions, due to an increase in DNA gyrase activity [68] or a decrease in topoisomerase I activity [77]. As shown by our phenotype observation in Figure 7, normal anoxic growth of *hupAB* strains suggests that, under these conditions of absence of oxygen, the superhelical DNA constraining activity of HU is not required for the organization of the bacterial nucleoid.

In aerobic conditions, however, the essential role of HU could be illustrated as follows. If we consider the presence of 30,000 HU dimers in the cell [6], [78], each covering a 9 bp sequence [79], it can be deduced that each of the 229 HU-regulated promoters accommodates 130 dimers, binding cooperatively, over a 1200 bp segment. In these conditions, the average spacing between HU binding sites on the chromosome would amount to ∼20 kb (Supplemental Figure S2). Interestingly, the bacterial nucleoid has been described as being shaped in domains of 50–100 kb [80] whereas more recent studies re-evaluated the organization of the chromosome in 400 supercoiled looped domains of ∼10 kb (reviewed in [81]). The formation of these high-order HU-DNA complexes has been observed by techniques as diverse as crystallography [69]–[71], atomic force microscopy [82] and fluorescence resonance energy transfer [83]. We therefore propose to localize the nucleoid-shaping and DNA-constraining roles of HU at the 229 chromosomal sites where transcription regulation occurs.

In conclusion, our data has shown that HU regulates the expression of 8% of the *E. coli* genome using two mechanisms. In the first, HU cooperates with known transcription regulators such as LexA, GadX of FNR and in the second, HU acts alone on its DNA structure targets. Our observation that HU is necessary in aerobiosis and dispensable in anoxic conditions unravels the important role played by this histone-like protein in the metabolism of the bacterial cell and opens new areas for research to be explored.

## Materials and Methods

### Plasmids, phages, bacterial strains and general growth conditions

The *E. coli* K-12 bacterial strains used in this work are listed in Table 1. New C600 (JO2057) derivatives carrying the mutated *hup* were constructed. JO2081 (*hupA*), JO2083 (*hupB*) and JO3020 (*hupAB*) were obtained by phage P1 transduction by selecting on LB agar plates containing the appropriate antibiotics. The C600 *hupAB* mutant displayed the characteristic small-colony and cell-filamentation phenotypes, as expected and observed previously [9]. The *hup* gene interruptions were verified for each construction by PCR analysis of genomic DNA extracted from each mutant (data not shown). The absence of the respective HU subunits was demonstrated by western blot immunodetection after SDS-PAGE and Acid Urea Triton-PAGE (data not shown). The *lacZ*, *fnr* and *recA* mutations, originating respectively from ENS303 [25], EF88 (Jeff Cole), and JR1713 [20], were introduced into the same genetic background by P1 transduction to generate respectively JO2039, JO3019 and JO3029. Due to the lack of phenotype in our laboratory culture conditions, the presence of the *fnr::*Tn*10* marker in JO3029 was verified by genomic PCR (data not known). To allow re-use of the tetracycline resistance marker, strain JO2039 was cured from its Tn*10* transposon by growth on fusaric acid medium to yield JO3027 as described [84]. Luria-Bertani (0.5% NaCl) broth and agar (15 g/liter) were used for routine growth. When used, ampicillin, tetracycline, kanamycin, and chloramphenicol were provided at final concentrations of 100, 12, 50, and 20 µg/ml, respectively. Anoxic growth conditions were achieved in a 2.5 liter Oxoid anaerobic jar (Model AG25) (Oxoid, Hampshire, UK) or in a Coy anaerobic chamber (Coy Enterprises, Inc.) using LB 0.5% NaCl containing 10 mM NaNO_3_ and 0.2% (wt/vol) glucose.

### Construction of strains carrying single copy promoter-*lacZ* fusions and mutant derivatives

The *lacZ* fusion strains used in this work are shown in Table 7. They were constructed as follows. *E. coli* chromosomal DNA was extracted and purified from strain JO2057, as described [85]. The cloning of the promoters of the genes of interest was assisted by the BAGET web service [86]. They were amplified by PCR from the chromosome with Pfu polymerase (Promega) using gene-specific primers flanked by EcoRI or BamHI restriction sites except in the case of *sulA* where the second primer was flanked by a BglII site due to the presence of a BamHI site in the amplified fragment. Theses oligonucleotides are described in Supplemental Table S20. The resulting PCR fragments were digested with EcoRI and BamHI (EcoRI and BglII for *sulA*) and directionally cloned into BamHI-EcoRI-digested *lacZ* operon fusion vector pRS415 [87]. All *lacZ* fusions were transferred from their respective plasmid to phage λRS45 by lytic rescue with the exception of the *dinI::lacZ* and *sulA::lacZ* fusions which were rescued on the non SOS-inducible λRS88 *ind^−^*. C600 *lacZ* lysogens were constructed with these fusion-carrying phages. Additional mutations were then introduced by P1-mediated transduction and selection for the appropriate antibiotic resistance. In order to avoid gene dosage interference caused by a variable number of fusion-carrying prophages, the strain derivatives were constructed sequentially using the original C600 *lacZ* lysogen as starting material, as indicated in Table 7.

**Table 7:** Synoptic view of the construction of the transcriptional *lacZ* fusions strains used in this work.

Fusion	2039+pRS415 Φ(fusion)	3027 [λRS88 Der.]	3027 [λRS45 Der.]	*ΔrecA::Tc*	*hupA::Cm*	*hupA::Cm*, *hupB::Km*	*Δfnr::*Tn*10*	*Δfnr::*Tn*10*, *hupA::Cm*	*Δfnr::*Tn*10*, *hupA::Cm, hupB::Km*
*sulA::lacZ*	3033	3057		3081 (3057+P1 3019)	3087 (3057+P1 2081)	3111 (3087+P1 2083)			
*dinI::lacZ*	3035	3059		3083 (3059+P1 3019)	3089 (3059+P1 2081)	3113 (3089+P1 2083)			
*lldP::lacZ*	3037		3061		3091 (3061+P1 2081)	3115 (3091+P1 2083)	3133 (3061+P1 3029)	3143 (3133+P1 2081)	3153 (3143+P1 2083)
*ndk::lacZ*	3039		3063		3093 (3063+P1 2081)	3117 (3093+P1 2083)	3135 (3063+P1 3029)	3145 (3135+P1 2081)	3155 (3145+P1 2083)
*nirB::lacZ*	3041		3065		3095 (3065+P1 2081)	3119 (3095+P1 2083)	3137 (3065+P1 3029)	3147 (3137+P1 2081)	3157 (3137+P1 2083)
*narG::lacZ*	3043		3067		3097 (3067+P1 2081)	3121 (3097+P1 2083)	3139 (3067+P1 3029)	3149 (3139+P1 2081)	3159 (3139+P1 2083)
*dcuC::lacZ*	3045		3069		3099 (3069+P1 2081)	3123 (3099+P1 2083)	3141 (3069+P1 3029)	3151 (3141+P1 2081)	3161 (3141+P1 2083)

Column 2 corresponds to strains carrying plasmids (derived from pRS415) and harboring the different transcriptional *lacZ* gene fusions used in this work. The strains listed in columns 3 and 4 correspond to single copy chromosomal derivatives of the same *lacZ* gene fusions, carried by a lambda prophage. The strains listed in columns 5 to 10 under their relevant genotype have been obtained by phage P1 transduction. The numbers between parentheses refer to the respective recipient and donor strains used for transduction. Four digit numbers refer to strains names; JO suffices have been omitted for clarity.

### Beta-galactosidase assay

Cell extracts were prepared from exponential phase cultures grown in 5 ml LB at 37°C, aerobically or anaerobically as described above. Assays of β-galalactosidase from these extracts were carried out as described [88], in triplicate.

### Acid resistance assay

The assay to measure the resistance of strains to low pH exposure was conducted in duplicate, as described [57].

### Microarray technology

Affymetrix GeneChips were chosen for the transcriptomic approach, since they provide a 15- to 40-fold probe redundancy for each individual gene to increase repeatability. In our hands, the correlation between duplicate experiments was statistically significant (see below). The four bacterial strains JO2057, JO2081, JO2083 and JO3020) described above were grown in 100 ml LB 0.5% NaCl at 200 rpm in a New Brunswick laboratory shaker in 2-liter flasks. LB medium was chosen over synthetic minimal for its better permissivity for the growth of *hupAB* mutants. The typical doubling time, observed in exponential phase, was 40 min for JO2057, JO2081, JO2083 and 75 min for JO3020. The various growth phase samples were collected at the following cell densities: exponential phase: OD_600_ 0.6–0.7; transition: 2.2–2.5 and stationary: 4.6–4.8 (3.0 for *hupAB*). Special care was taken to process the samples immediately for total RNA extraction to ensure optimal representation of short lived messenger species. The protocol for RNA extraction was adapted form [36]. Briefly, a culture volume of 7 ml was mixed with the same volume of boiling 2% SDS, 4 mM EDTA and heated at 100°C for 3 to 5 min then vortexed cooled first?. At this stage, the extract was either processed further or stored at −20°C. Seven milliliter of phenol/water were added before incubating 10 min at 67°C with occasional stirring. The samples were cooled on ice and centrifuged 10 min at 5000 rpm at 4°C. The aqueous phase was separated, extracted the same way and then once with phenol/chloroform (v/v 1∶1). One tenth volume of 4 M NaCl and 2.5 volumes of cold ethanol were then added to the aqueous phase. The tubes were left at −20°C for two hrs and then centrifuged at 8500 rpm at 4°C. The pellet was washed with 70% ethanol, dried under vacuum, and resuspended in 300 µl sterile water and transferred to an eppendorf tube. Qiagen RDD buffer (34.5 µl) and of RNase free DNase I (9.37 µl, Qiagen) were added. After 15 min at room temperature, the tubes were mixed by inversion and deproteinized as described above with 300 µl phenol/H_2_O at room temperature. The RNA was then precipitated with 37.5 µl NaCl 4 M and 823 µl cold ethanol. After 2 hrs at −20°C, the tubes were centrifuged 30 min at 10,000×g at 4°C, the pellets were then washed with 70% ethanol then dried under vacuum and resuspended in 60 µl sterile water. The RNAs were stored at −20°C. RNA purity was assessed by measuring the A_260_/A_280_ ratio and selecting them within a range of 1.8 to 2.1. Samples with a ratio lower than of 1.8 were discarded. RNA samples were reverse transcribed and biotinylated according to the Affymetrix protocol. Biotin-labeled cDNA (2.5 µg) was hybridized to *E. coli* antisense genome arrays (Affymetrix) at 45°C for 16 h as recommended in the GeneChip technical manual (Affymetrix). The probed arrays were scanned at 570 nm using a confocal laser scanner (Hewlett-Packard G2500A). Microarray Suite 5.0 software (Affymetrix) was used to determine the gene expression levels. The Affymetrix Genechips were used for this purpose as follows. The most relevant experiments were carried out in duplicate: the wild type (JO2057) and the *hupAB* (JO3020) strains were tested in the exponential and stationary phase. Wild type and *hupAB* strains were also tested in single experiments at the transition phase. The last chips were used to test, respectively, the single *hupA* (JO2081) and single *hupB* (JO2083) mutants at the three phases.

### Data driven, unsupervised statistical methodology

Affymetrix microarray hybridization signals were normalized with dChip [89]. Hybridization signals and detection calls in MIAME-compliant format have been deposited in the NCBI GEO database (accession #GSE11183). A total of 4368 annotated genes were further processed. Due to the large number of regulated genes, we used a very restrictive selection criterion as follows: the genes whose expression varied significantly in at least one of the conditions were identified by comparing their maximal (MaxVal) and minimal (MinVal) expression values in each experimental condition with the following criterion:
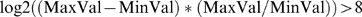
derived from fold filters used for genes selection. Genes were selected for further analysis if they presented both relative (MaxVal/MinVal) and absolute (MaxVal - MinVal) variations [37]. The value of 8 was selected empirically as a threshold based on an histogram showing the number of genes as a function of the expression value (MaxVal−MinVal)/(MaxVal/MinVal). Gene Cluster 3.0 allowed us to cluster variable genes using K-Means with the Pearson correlation [37]; they were visualized with Java Treeview [90]. The determination of the number of clusters was determined by using the iterative criterion of Hartigan:
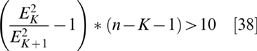



Since the statistical distribution of values in the data did not obey the normal law, bootstrap methods provided by Stata Statistical Software R. 9 [91] were used to obtain a more robust non-parametric estimate of the confidence intervals [92]. In order to determine which experimental condition effect (genotype and growth phase) was predominant in each cluster, we performed Kruskall-Wallis non-parametric tests for every condition except *hupA* vs. *hupB*. A total of 30 conditions were therefore tested (10 for each phase) to assess the significance of the difference in gene expression between clusters. When the overall test was significant, the genes belonging to the clusters presenting very high mean ranks were considered to be regulated under the given condition. Microarray reproducibility was tested using intra-class coefficients; all Spearman's rhos were between 0.89 and 0.95 indicating very high data reproducibility. The absolute gene expression values are shown in Supplemental Table 2. For clarity, the individual gene expression levels in Tables 2 to [Table pone-0004367-t003] [Table pone-0004367-t004] [Table pone-0004367-t005] 6 have been normalized by taking, for each growth phase, a value of 1 for the wild-type strain.

## Supporting Information

Figure S1 Comparison of the HU, H-NS, Fis and supercoiling regulons
(1.55 MB TIF)

Figure S2 Distribution of the HU regulated operons on the E. coli chromosome.
(1.64 MB TIF)

Table S1 Cluster assignment by the Kruskall-Wallis tests.
(0.05 MB DOC)

Table S2 Comparison of the RpoS regulon (Saint-Ruf et al, 2004) with the clusters of te HU regulon^(1)^.
(0.14 MB DOC)

Table S3 Genes composing the HU regulon
(0.92 MB DOC)

Table S4 Operons composing the HU regulon
(0.32 MB DOC)

Table S5 Chaperone and stress functions in the HU regulon.
(0.27 MB DOC)

Table S6 Comparison of the genes regulated by DNA supercoiling by Blot *et al* (2006) ^(1)^ and Peter *et al* (2004) ^(2)^
(0.08 MB DOC)

Table S7 Comparison of the genes regulated by HU ^(1)^ and by DNA supercoiling by Blot *et al* (2006) ^(2)^.
(0.03 MB DOC)

Table S8 Comparison of the genes regulated by HU ^(1)^ and by DNA supercoiling by Peter *et al* (2004) ^(2)^
(0.04 MB DOC)

Table S9 Comparison of the genes regulated by HU ^(1)^ and by DNA supercoiling by Blot *et al* (2006) ^(2)^ and Peter *et al* (2004) ^(3)^
(0.03 MB DOC)

Table S10 Comparison of the genes regulated by H-NS ^(1)^ and by DNA supercoiling by Blot *et al* (2006) ^(2)^
(0.05 MB DOC)

Table S11 Comparison of the genes regulated by H-NS ^(1)^ and by DNA supercoiling by Peter *et al* (2004) ^(2)^
(0.09 MB DOC)

Table S12 Comparison of the genes regulated by FIS ^(1)^ and by DNA supercoiling by Blot *et al* (2006) ^(2)^
(0.07 MB DOC)

Table S13 Comparison of the genes regulated by FIS ^(1)^ and by DNA supercoiling by Peter *et al* (2004) ^(2)^
(0.10 MB DOC)

Table S14 Comparison of the genes regulated by FIS ^(1)^ and by DNA supercoiling by Blot *et al* (2006) ^(2)^ and Peter *et al* (2004) ^(3)^
(0.03 MB DOC)

Table S15 Comparison of the genes regulated by HU ^(1)^ and by H-NS (Blot *et al*, 2006 ^(2)^)
(0.14 MB DOC)

Table S16 Comparison of the genes regulated by HU ^(1)^ and by FIS (Blot *et al*, 2006 ^(2)^)
(0.16 MB DOC)

Table S17 Comparison of the genes regulated by H-NS ^(1)^ and FIS^(2)^ (Blot *et al*, 2006)
(0.22 MB DOC)

Table S18 Comparison of the genes regulated by HU^(1)^, H-NS ^(2)^ and FIS^(3)^ (Blot *et al*, 2006)
(0.07 MB DOC)

Table S19 Comparison of the HU regulon with the genes located in the chromosomal areas exhibiting maximum DNA distorsion reported by Pedersen *et al*, (2000).
(0.06 MB DOC)

Table S20 Oligonucleotides used for PCR amplification.
(0.03 MB DOC)
