# Supplementary figures and images for: The HU Regulon Is Composed of Genes Responding to Anaerobiosis, Acid Stress, High Osmolarity and SOS Induction
Source: PLoS One. 2009 Feb 4;4(2):e4367. doi: 10.1371/journal.pone.0004367 (PMC2634741; doi:10.1371/journal.pone.0004367)

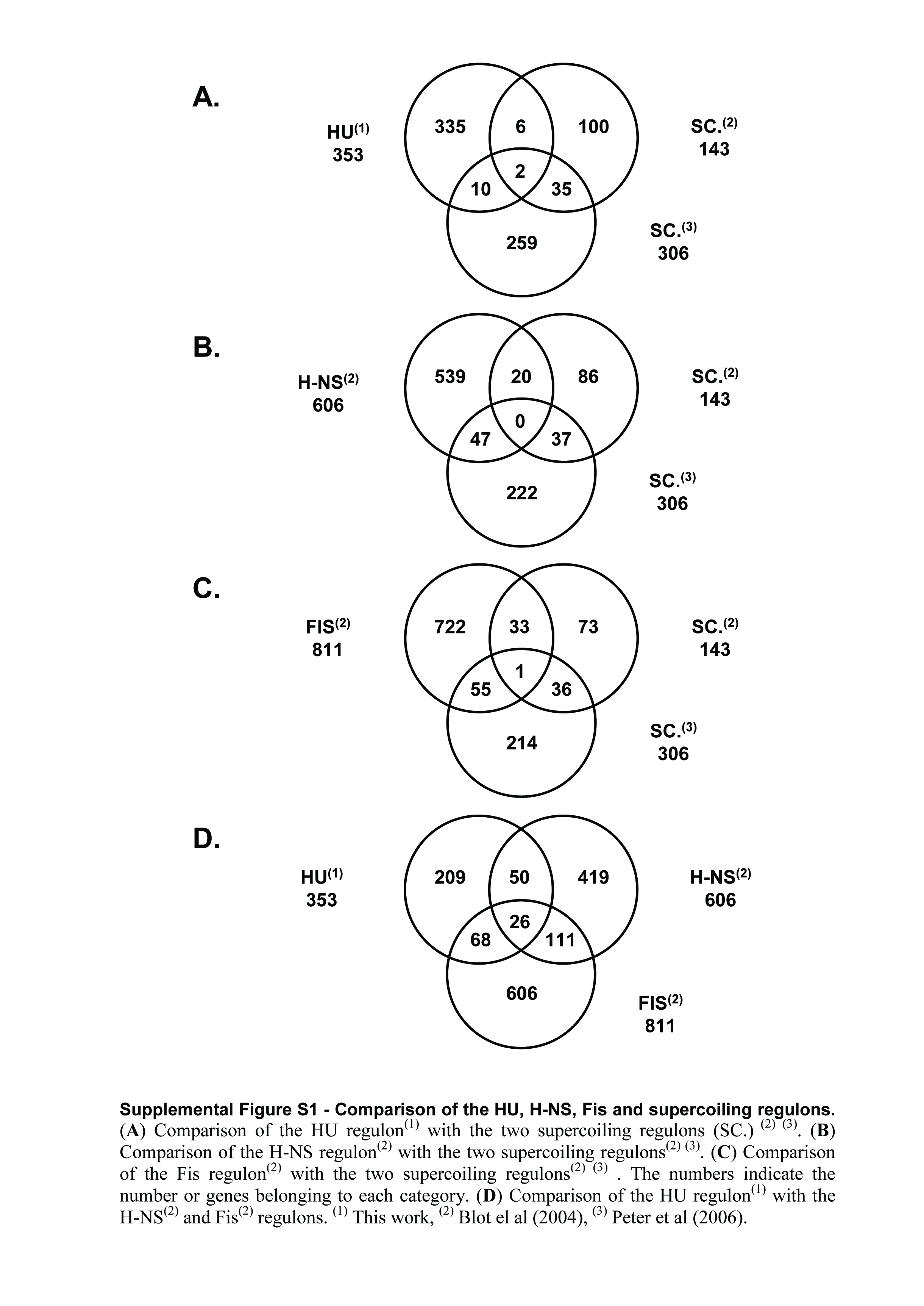

Supplement: Figure S1 — Comparison of the HU, H-NS, Fis and supercoiling regulons (1.55 MB TIF) [file pone.0004367.s001.tif]

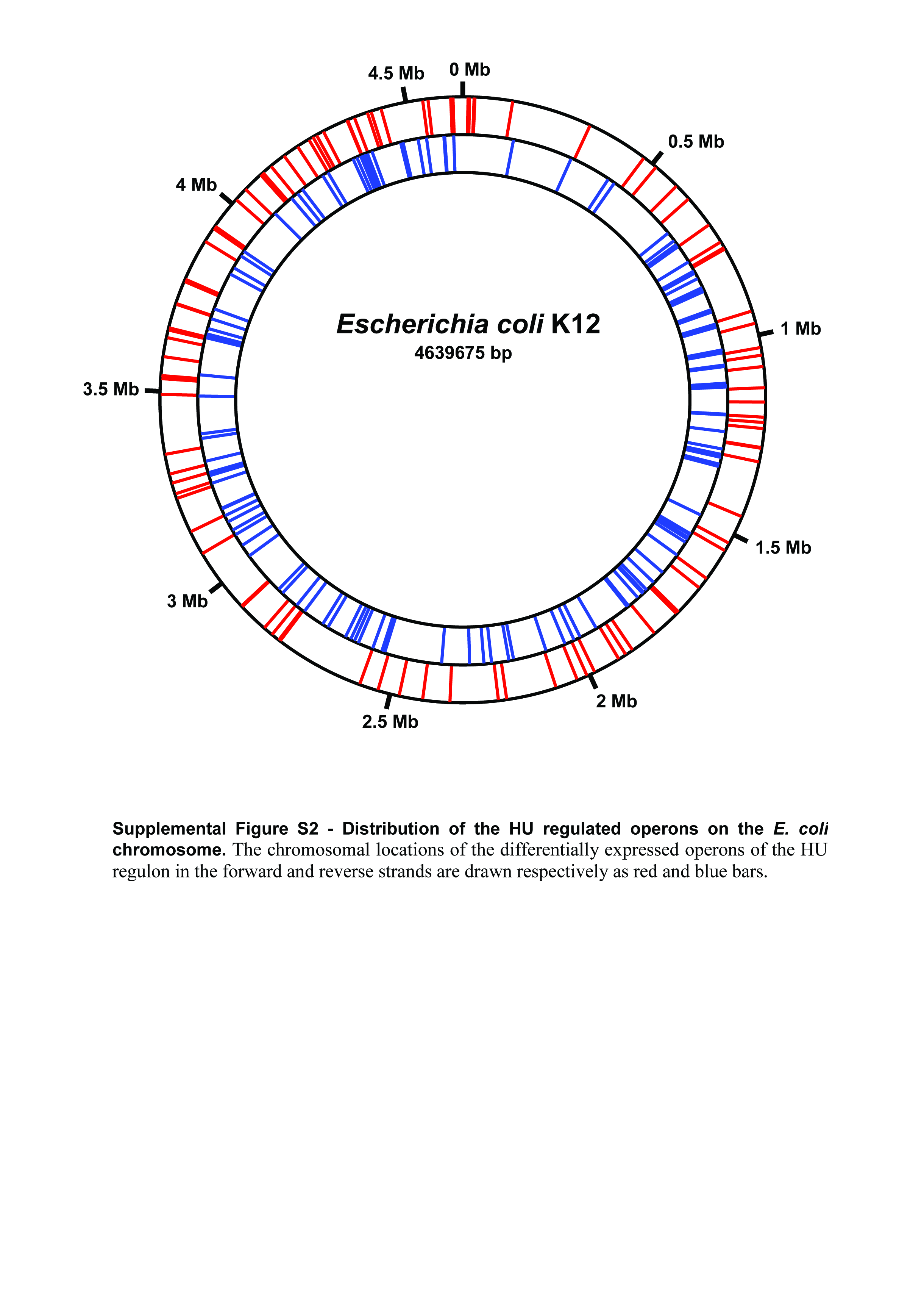

Supplement: Figure S2 — Distribution of the HU regulated operons on the E. coli chromosome. (1.64 MB TIF) [file pone.0004367.s002.tif]
