# Supplementary material for: The HU Regulon Is Composed of Genes Responding to Anaerobiosis, Acid Stress, High Osmolarity and SOS Induction
Source: PLoS One. 2009 Feb 4;4(2):e4367. doi: 10.1371/journal.pone.0004367 (PMC2634741; doi:10.1371/journal.pone.0004367)
Supplement: Table S1 — Cluster assignment by the Kruskall-Wallis tests. (0.05 MB DOC) [file pone.0004367.s003.doc]

**Supplemental Table S1. Cluster assignment by the Kruskall-Wallis tests**.

| **Phase** | **Genotype** | **WT** | ***hupA*** | ***hupB*** | ***hupAB*** |
| --- | --- | --- | --- | --- | --- |
| **Exponential** | **WT** | - | Cluster 5 | NS | Cluster 6>5 |
|  | ***hupA*** | Cluster 6 | - | NT | 6 |
|  | ***hupB*** | NS | NT | - | Cluster 6>5 |
|  | ***hupAB*** | Cluster 4 | Cluster 5 | Cluster 4 | - |
| **Transition** | **WT** | - | NS | NS | NS |
|  | ***hupA*** | NS | - | NT | NS |
|  | ***hupB*** | NS | NT | - | NS |
|  | ***hupAB*** | Cluster 7 | Cluster 7 | NS | - |
| **Stationary** | **WT** | - | NS | Cluster 2 | NS |
|  | ***hupA*** | NS | - | NT | NS |
|  | ***hupB*** | NS | NT | - | NS |
|  | ***hupAB*** | NS | Cluster 2 | Cluster 2 | - |

For each condition (phase and genotype), the Kruskall-Wallis analysis of variance by ranks permitted to assess differences in differential gene between clusters. In case of overall significance, one or two clusters were assigned to a condition. NT: not tested; NS: not significant.
