# Supplementary material for: The HU Regulon Is Composed of Genes Responding to Anaerobiosis, Acid Stress, High Osmolarity and SOS Induction
Source: PLoS One. 2009 Feb 4;4(2):e4367. doi: 10.1371/journal.pone.0004367 (PMC2634741; doi:10.1371/journal.pone.0004367)
Supplement: Table S2 — Comparison of the RpoS regulon (Saint-Ruf et al, 2004) with the clusters of te HU regulon(1). (0.14 MB DOC) [file pone.0004367.s004.doc]

**Supplemental Table S2. Comparison of the RpoS regulon (Saint-Ruf et al, 2004) with the clusters of te HU regulon^(1)^.**

| **Gene** | **Blattner** | **Function** | **Class** | **Reg.^(1)^** |
| --- | --- | --- | --- | --- |
| *htgA* | b0012 | Heat-shock protein | Adaptations: atypical conditions | - |
| *htpX* | b1829 | Heat-shock protein | Adaptations: atypical conditions | [Cluster1] |
| *ibpB* | b3686 | Heat-shock protein | Adaptations: atypical conditions | [Cluster5] |
| *ibpA* | b3687 | Heat-shock protein | Adaptations: atypical conditions | [Cluster5] |
| *groES* | b4142 | Heat-shock protein | Adaptations: atypical conditions | [Cluster5] |
| *hslV* | b3932 | Heat-shock protein | Adaptations: atypical conditions | [Cluster5] |
| *oxyR* | b3961 | H2O2-inducible genes activator | Adaptations: atypical conditions | - |
| *sodA* | b3908 | Manganese superoxide dismutase | Adaptations: atypical conditions | [Cluster6] |
| *soxR* | b4063 | Regulatory protein of SoxRS regulon | Adaptations: atypical conditions | - |
| *yfiA* | b2597 | Ribosome binding protein, H2O2 inducible | Adaptations: atypical conditions | [Cluster9] |
| *rpoH* | b3461 | RNA polymerase sigma-32 subunit | Adaptations: atypical conditions | - |
| *ydcH* | b1426 | Unknown, H2O2 inducible | Adaptations: atypical conditions | - |
| *dinJ* | b0226 | Damage-inducible protein J | DNA repair | - |
| *dinG* | b0799 | DNA-damage-inducible protein G | DNA repair | - |
| *nfo* | b2159 | Endonuclease IV | DNA repair | - |
| *sbcB* | b2011 | Exodeoxyribonuclease I | DNA repair | - |
| *xseB* | b0422 | Exodeoxyribonuclease small subunit | DNA repair | - |
| *recB* | b2820 | Exonuclease V subunit | DNA repair | - |
| *ung* | b2580 | Uracil-DNA glycosylase | DNA repair | - |
| *holC* | b4259 | DNA polymerase III, xhi-subunit | Replication-transcription | - |
| *fis* | b3261 | Factor for inversion stimulation | Replication-transcription | - |
| *rpoA* | b3295 | RNA polymerase, alpha-subunit | Replication-transcription | [Cluster8] |
| *rpoB* | b3987 | RNA polymerase, beta-subunit | Replication-transcription | - |
| *rpoC* | b3988 | RNA polymerase, beta prime-subunit | Replication-transcription | - |
| *sucA* | b0726 | 2-Oxoglutarate dehydrogenase | TCA cycle | [Cluster5] |
| *acnB* | b0118 | Aconitate hydratase 2 | TCA cycle | - |
| *acnA* | b1276 | Citrate hydro-lyase 1 | TCA cycle | - |
| *gltA* | b0720 | Citrate synthase | TCA cycle | [Cluster5] |
| *sucB* | b0727 | Dihydrolipoamide succinyltransferase | TCA cycle | [Cluster5] |
| *fumC* | b1611 | Fumarate hydratase class II | TCA cycle | [Cluster3] |
| *mdh* | b3236 | Malate dehydrogenase | TCA cycle | [Cluster9] |
| *sdhC* | b0721 | Succinate dehydrogenase | TCA cycle | [Cluster5] |
| *sdhD* | b0722 | Succinate dehydrogenase | TCA cycle | [Cluster5] |
| *sdhA* | b0723 | Succinate dehydrogenase flavoprotein | TCA cycle | [Cluster5] |
| *sdhB* | b0724 | Succinate dehydrogenase iron-sulfur protein | TCA cycle | [Cluster5] |
| *sucD* | b0729 | Succinyl-coA synthetase alpha-chain | TCA cycle | [Cluster5] |
| *sucC* | b0728 | Succinyl-coA synthetase beta-chain | TCA cycle | [Cluster5] |
| *ompF* | b0929 | Outer membrane porin F | Outer membrane constituents | [Cluster8] |
| *pspA* | b1304 | Phage shock protein A | Phage shock proteins | - |
| *pspB* | b1305 | Phage shock protein B | Phage shock proteins | - |
| *pspC* | b1306 | Phage shock protein C | Phage shock proteins | - |
| *pspD* | b1307 | Phage shock protein D | Phage shock proteins | - |
| *rpsC* | b3314 | 30S subunit protein S3 | Ribosomal proteins | [Cluster8] |
| *rpsE* | b3303 | 30S subunit protein S5 | Ribosomal proteins | [Cluster8] |
| *rpsG* | b3341 | 30S subunit protein S7 | Ribosomal proteins | [Cluster8] |
| *rpsH* | b3306 | 30S subunit protein S8 | Ribosomal proteins | [Cluster8] |
| *rpsN* | b3307 | 30S subunit protein S14 | Ribosomal proteins | [Cluster8] |
| *rpsP* | b2609 | 30S subunit protein S16 | Ribosomal proteins | [Cluster8] |
| *rpsS* | b3316 | 30S subunit protein S19 | Ribosomal proteins | [Cluster8] |
| *rplA* | b3984 | 50S subunit protein L1 | Ribosomal proteins | [Cluster8] |
| *rplB* | b3317 | 50S subunit protein L2 | Ribosomal proteins | [Cluster8] |
| *rplC* | b3320 | 50S subunit protein L3 | Ribosomal proteins | [Cluster8] |
| *rplF* | b3305 | 50S subunit protein L6 | Ribosomal proteins | [Cluster8] |
| *rplJ* | b3985 | 50S subunit protein L10 | Ribosomal proteins | [Cluster8] |
| *rplK* | b3983 | 50S subunit protein L11 | Ribosomal proteins | [Cluster8] |
| *rplL* | b3986 | 50S subunit protein L7/L12 | Ribosomal proteins | [Cluster8] |
| *rplO* | b3301 | 50S subunit protein L15 | Ribosomal proteins | [Cluster8] |
| *rplP* | b3313 | 50S subunit protein L16 | Ribosomal proteins | [Cluster8] |
| *rplS* | b2606 | 50S subunit protein L19 | Ribosomal proteins | [Cluster8] |
| *rplW* | b3318 | 50S subunit protein L23 | Ribosomal proteins | [Cluster8] |
| *rplX* | b3309 | 50S subunit protein L24 | Ribosomal proteins | [Cluster8] |
| *rplY* | b2185 | 50s subunit protein L25 | Ribosomal proteins | [Cluster8] |
| *rpmC* | b3312 | 50S subunit protein L29 | Ribosomal proteins | [Cluster8] |
| *prmA* | b3259 | Ribosomal protein L11 | Ribosomal proteins | - |
