# Supplementary material for: The HU Regulon Is Composed of Genes Responding to Anaerobiosis, Acid Stress, High Osmolarity and SOS Induction
Source: PLoS One. 2009 Feb 4;4(2):e4367. doi: 10.1371/journal.pone.0004367 (PMC2634741; doi:10.1371/journal.pone.0004367)
Supplement: Table S3 — Genes composing the HU regulon (0.92 MB DOC) [file pone.0004367.s005.doc]

| **Supplemental Table S3.** Genes composing the HU regulon | | | | | | | | | | | | | | | | |
| --- | --- | --- | --- | --- | --- | --- | --- | --- | --- | --- | --- | --- | --- | --- | --- | --- |
|  | | | | **Exponential** | | | | **Transition** | | | | **Stationary** | | | |  |
| **Gene** | **Blattner** | **Operon** | **Cluster** | **WT** | ***hupB*** | ***hupA*** | ***hupAB*** | **WT** | ***hupB*** | ***hupA*** | ***hupAB*** | **WT** | ***hupB*** | ***hupA*** | ***hupAB*** | **Definition** |
| *nhaA* | b0019 | *nhaAR* | 2 | 94 | 103 | 83 | 148 | 86 | 140 | 113 | 123 | 398 | 889 | 938 | 94 | Na+/H antiporter, pH dependent |
| *ybaY* | b0453 | *ybaY* | 2 | 52 | 54 | 76 | 67 | 57 | 80 | 54 | 60 | 678 | 1679 | 1313 | 52 | glycoprotein/polysaccharide metabolism |
| *ybaS* | b0485 | *ybaST* | 2 | 57 | 59 | 45 | 71 | 66 | 309 | 107 | 88 | 1419 | 2645 | 2929 | 57 | putative glutaminase |
| *ybaT* | b0486 | *ybaST* | 2 | 47 | 73 | 30 | 54 | 29 | 110 | 38 | 64 | 595 | 824 | 821 | 47 | putative amino acid/amine transport protein |
| *entC* | b0593 | *entCEBA-ybdB* | 2 | 181 | 189 | 196 | 214 | 197 | 196 | 197 | 211 | 403 | 429 | 433 | 181 | isochorismate hydroxymutase 2, enterochelin biosynthesis |
| *entE* | b0594 | *entCEBA-ybdB* | 2 | 84 | 78 | 66 | 171 | 90 | 98 | 103 | 118 | 443 | 440 | 505 | 84 | 2,3-dihydroxybenzoate-AMP ligase |
| *entB* | b0595 | *entCEBA-ybdB* | 2 | 99 | 99 | 122 | 229 | 107 | 109 | 108 | 95 | 739 | 667 | 653 | 99 | 2,3-dihydro-2,3-dihydroxybenzoate synthetase, isochroismatase |
| *entA* | b0596 | *entCEBA-ybdB* | 2 | 6 | 6 | 6 | 48 | 10 | 13 | 8 | 6 | 260 | 267 | 253 | 6 | 2,3-dihydro-2,3-dihydroxybenzoate dehydrogenase, enterochelin biosynthesis |
| *ybdB* | b0597 | *entCEBA-ybdB* | 2 | 71 | 90 | 35 | 155 | 51 | 63 | 80 | 75 | 508 | 515 | 573 | 71 | hypothetical protein |
| *ybgS* | b0753 | *ybgS* | 2 | 46 | 39 | 41 | 52 | 40 | 41 | 46 | 62 | 1037 | 1896 | 2581 | 46 | putative homeobox protein |
| *ybhB* | b0773 | *ybhB* | 2 | 207 | 189 | 248 | 198 | 361 | 445 | 401 | 80 | 211 | 319 | 269 | 207 | hypothetical protein |
| *ybiI* | b0803 | *ybiI* | 2 | 57 | 68 | 66 | 68 | 64 | 81 | 55 | 52 | 155 | 247 | 203 | 57 | hypothetical protein |
| *ybiM* | b0806 | *ybiM* | 2 | 366 | 417 | 318 | 430 | 308 | 359 | 338 | 441 | 423 | 575 | 639 | 366 | hypothetical protein |
| *dps* | b0812 | *dps* | 2 | 549 | 608 | 428 | 308 | 1035 | 2631 | 1617 | 308 | 9936 | 11869 | 9847 | 549 | global regulator, starvation conditions |
| *ybjP* | b0865 | *ybjP* | 2 | 89 | 80 | 120 | 67 | 145 | 212 | 149 | 86 | 607 | 1046 | 623 | 89 | putative enzyme |
| *poxB* | b0871 | *poxB-ltaE-ybjT* | 2 | 121 | 114 | 112 | 130 | 127 | 173 | 175 | 143 | 855 | 1659 | 1865 | 121 | pyruvate oxidase |
| *ycaC* | b0897 | *ycaC* | 2 | 66 | 117 | 67 | 75 | 70 | 87 | 73 | 69 | 1319 | 2438 | 2279 | 66 | hypothetical protein |
| *hspQ* | b0966 | *hspQ* | 2 | 340 | 263 | 564 | 442 | 405 | 563 | 363 | 535 | 1674 | 3662 | 3832 | 340 | diguanylate cyclase |
| *hyaA* | b0972 | *hyaABCDEF* | 2 | 71 | 82 | 66 | 74 | 94 | 196 | 80 | 86 | 122 | 162 | 124 | 71 | hydrogenase-1 small subunit |
| *cbpA* | b1000 | *cbpAM* | 2 | 274 | 272 | 249 | 193 | 362 | 666 | 357 | 208 | 1065 | 1549 | 1872 | 274 | curved DNA-binding protein functions closely related to DnaJ |
| *wrbA* | b1004 | *wrbA-yccJ* | 2 | 396 | 537 | 93 | 108 | 521 | 1096 | 660 | 110 | 1800 | 2799 | 2059 | 396 | trp repressor binding protein affects association of trp repressor and operator |
| *msyB* | b1051 | *msyB* | 2 | 260 | 230 | 208 | 277 | 301 | 445 | 414 | 344 | 2723 | 5733 | 5521 | 260 | acidic protein suppresses mutants lacking function of protein export |
| *grxB* | b1064 | *grxB* | 2 | 323 | 419 | 314 | 234 | 432 | 1108 | 556 | 281 | 1645 | 2306 | 2064 | 323 | glutaredoxin 2 |
| *ycgZ* | b1164 | *ycgZ* | 2 | 7 | 17 | 50 | 31 | 9 | 11 | 8 | 33 | 51 | 1430 | 620 | 7 | hypothetical protein |
| *ymgA* | b1165 | *ymgAB* | 2 | 220 | 196 | 203 | 270 | 293 | 235 | 273 | 347 | 542 | 1995 | 1565 | 220 | hypothetical protein |
| *ymgB* | b1166 | *ymgAB* | 2 | 9 | 11 | 26 | 19 | 7 | 5 | 6 | 24 | 111 | 909 | 318 | 9 | hypothetical protein |
| *ymgC* | b1167 | *ymgC* | 2 | 4 | 3 | 6 | 5 | 2 | 6 | 3 | 5 | 12 | 193 | 88 | 4 | hypothetical protein |
| *yciE* | b1257 | *yciGFE* | 2 | 23 | 18 | 13 | 23 | 32 | 47 | 24 | 30 | 227 | 856 | 463 | 23 | hypothetical protein |
| *yciF* | b1258 | *yciGFE* | 2 | 19 | 19 | 19 | 20 | 22 | 35 | 32 | 36 | 366 | 1558 | 1184 | 19 | putative structural proteins |
| *yciG* | b1259 | *yciGFE* | 2 | 19 | 23 | 19 | 24 | 29 | 22 | 23 | 35 | 783 | 2453 | 2580 | 19 | hypothetical protein |
| *trpL* | b1265 | *trpLEDCBA* | 2 | 37 | 41 | 28 | 39 | 21 | 17 | 28 | 32 | 70 | 254 | 158 | 37 | trp operon leader peptide |
| *osmB* | b1283 | *osmB* | 2 | 78 | 85 | 74 | 225 | 92 | 108 | 75 | 415 | 727 | 2350 | 2492 | 78 | osmotically inducible lipoprotein |
| *yciW* | b1287 | *yciW* | 2 | 128 | 88 | 314 | 235 | 98 | 127 | 134 | 438 | 224 | 791 | 1083 | 128 | putative oxidoreductase |
| *yncG* | b1454 | *yncG* | 2 | 16 | 21 | 3 | 4 | 9 | 4 | 4 | 7 | 60 | 124 | 71 | 16 | putative transferase |
| *adhP* | b1478 | *adhP* | 2 | 72 | 92 | 71 | 76 | 79 | 96 | 83 | 56 | 1529 | 3267 | 3154 | 72 | alcohol dehydrogenase |
| *sra* | b1480 | *bdm-sra* | 2 | 310 | 276 | 240 | 289 | 579 | 1298 | 1026 | 571 | 7070 | 9940 | 9087 | 310 | 30S ribosomal subunit protein S22 |
| *bdm* | b1481 | *bdm-sra* | 2 | 6 | 3 | 7 | 6 | 6 | 8 | 7 | 14 | 147 | 602 | 836 | 6 | biofilm-dependent modulation protein |
| *yddV* | b1490 | *yddV-dos* | 2 | 29 | 24 | 42 | 41 | 18 | 20 | 14 | 21 | 106 | 188 | 166 | 29 | hypothetical protein |
| *gadC* | b1492 | *gadBC* | 2 | 20 | 22 | 22 | 26 | 29 | 371 | 36 | 31 | 3381 | 6207 | 5671 | 20 | GadC GABA APC transporter |
| *gadB* | b1493 | *gadBC* | 2 | 12 | 7 | 9 | 25 | 26 | 601 | 36 | 16 | 3665 | 7747 | 8227 | 12 | glutamate decarboxylase isozyme |
| *ydeI* | b1536 | *ydeI* | 2 | 47 | 43 | 36 | 42 | 56 | 42 | 61 | 71 | 229 | 435 | 453 | 47 | hypothetical protein |
| *sodC* | b1646 | *sodC* | 2 | 198 | 230 | 161 | 123 | 253 | 240 | 219 | 223 | 1023 | 1841 | 1876 | 198 | superoxide dismutase precursor (Cu-Zn) |
| *ydhL* | b1648 | *ydhL* | 2 | 33 | 27 | 20 | 28 | 31 | 37 | 27 | 35 | 93 | 141 | 104 | 33 | hypothetical protein |
| *cfa* | b1661 | *cfa* | 2 | 217 | 209 | 135 | 75 | 392 | 1148 | 435 | 84 | 664 | 1808 | 1423 | 217 | cyclopropane fatty acyl phospholipid synthase |
| *sufE* | b1679 | *sufABCDSE* | 2 | 54 | 49 | 77 | 76 | 30 | 31 | 14 | 39 | 280 | 312 | 356 | 54 | hypothetical protein |
| *ydiH* | b1685 | *ydiJIH* | 2 | 174 | 117 | 76 | 65 | 193 | 640 | 335 | 134 | 970 | 1859 | 1773 | 174 | hypothetical protein |
| *katE* | b1732 | *katE* | 2 | 52 | 47 | 43 | 53 | 45 | 62 | 64 | 69 | 1167 | 1944 | 1606 | 52 | catalase hydroperoxidase HPII(III) |
| *osmE* | b1739 | *osmE* | 2 | 145 | 123 | 138 | 138 | 127 | 563 | 203 | 169 | 1553 | 2543 | 2767 | 145 | activator of ntrL gene |
| *spy* | b1743 | *spy* | 2 | 374 | 408 | 342 | 405 | 484 | 468 | 434 | 499 | 982 | 1313 | 1586 | 374 | periplasmic protein related to spheroblast formation |
| *yebV* | b1836 | *yebV* | 2 | 57 | 49 | 44 | 57 | 54 | 87 | 63 | 81 | 1536 | 3602 | 3037 | 57 | hypothetical protein |
| *otsA* | b1896 | *otsBA* | 2 | 192 | 249 | 164 | 318 | 396 | 225 | 308 | 222 | 489 | 610 | 606 | 192 | trehalose-6-phosphate synthase |
| *otsB* | b1897 | *otsBA* | 2 | 53 | 55 | 45 | 58 | 40 | 88 | 84 | 96 | 843 | 1772 | 2347 | 53 | trehalose-6-phosphate phophatase, biosynthetic |
| *yodC* | b1957 | *yodC* | 2 | 87 | 71 | 85 | 77 | 55 | 106 | 76 | 91 | 671 | 1751 | 1814 | 87 | hypothetical protein |
| *hchA* | b1967 | *hchA* | 2 | 118 | 147 | 152 | 195 | 139 | 177 | 143 | 385 | 2007 | 4406 | 3554 | 118 | hypothetical protein |
| *fbaB* | b2097 | *fbaB* | 2 | 101 | 90 | 148 | 100 | 170 | 453 | 268 | 185 | 2967 | 3996 | 4639 | 101 | fructose bisphosphate aldolase monomer |
| *yehE* | b2112 | *yehE* | 2 | 45 | 44 | 47 | 33 | 44 | 69 | 57 | 46 | 213 | 698 | 421 | 45 | hypothetical protein |
| *mlrA* | b2127 | *mlrA* | 2 | 164 | 197 | 176 | 162 | 218 | 198 | 178 | 218 | 266 | 387 | 384 | 164 | putative transcriptional regulator |
| *elaB* | b2266 | *elaB* | 2 | 105 | 111 | 69 | 78 | 95 | 355 | 255 | 87 | 1979 | 3445 | 3223 | 105 | hypothetical protein |
| *yfcG* | b2302 | *yfcG* | 2 | 34 | 29 | 30 | 27 | 29 | 33 | 38 | 28 | 144 | 261 | 189 | 34 | putative S-transferase |
| *talA* | b2464 | *talA* | 2 | 68 | 76 | 119 | 87 | 90 | 206 | 140 | 108 | 2406 | 4660 | 4452 | 68 | transaldolase A |
| *tktB* | b2465 | *tktB* | 2 | 109 | 102 | 109 | 120 | 87 | 141 | 145 | 160 | 1538 | 2633 | 2777 | 109 | transketolase 2 isozyme |
| *ygaU* | b2665 | *ygaU* | 2 | 47 | 52 | 129 | 98 | 75 | 200 | 132 | 101 | 1617 | 3075 | 3189 | 47 | hypothetical protein |
| *ygaM* | b2672 | *ygaM* | 2 | 127 | 108 | 79 | 145 | 134 | 217 | 219 | 183 | 1112 | 2173 | 2144 | 127 | hypothetical protein |
| *cysD* | b2752 | *cysDNC* | 2 | 145 | 53 | 1094 | 685 | 163 | 153 | 357 | 1203 | 534 | 1598 | 2580 | 145 | ATP:sulfurylase (ATP:sulfate adenylyltransferase), subunit 2 |
| *glcD* | b2979 | *glcDEFGBA* | 2 | 193 | 201 | 243 | 210 | 384 | 265 | 240 | 220 | 431 | 311 | 352 | 193 | glycolate oxidase subunit D |
| *ygiW* | b3024 | *ygiW* | 2 | 183 | 201 | 147 | 149 | 226 | 418 | 245 | 131 | 2406 | 5156 | 3872 | 183 | hypothetical protein |
| *yqjC* | b3097 | *yqjCDEK* | 2 | 481 | 511 | 372 | 488 | 491 | 931 | 714 | 443 | 2112 | 3934 | 3745 | 481 | hypothetical protein |
| *yqjD* | b3098 | *yqjCDEK* | 2 | 282 | 352 | 299 | 289 | 390 | 738 | 520 | 286 | 1745 | 3102 | 2715 | 282 | hypothetical protein |
| *yqjE* | b3099 | *yqjCDEK* | 2 | 254 | 290 | 226 | 229 | 297 | 466 | 333 | 236 | 1244 | 2047 | 1902 | 254 | hypothetical protein |
| *yhbO* | b3153 | *yhbO* | 2 | 32 | 41 | 42 | 36 | 33 | 41 | 32 | 42 | 166 | 428 | 611 | 32 | hypothetical protein |
| *yrbL* | b3207 | *yrbL* | 2 | 298 | 314 | 653 | 752 | 334 | 857 | 627 | 490 | 1886 | 3172 | 3081 | 298 | hypothetical protein |
| *yhcO* | b3239 | *yhcO* | 2 | 16 | 13 | 15 | 19 | 20 | 26 | 30 | 41 | 172 | 412 | 282 | 16 | hypothetical protein |
| *fic* | b3361 | *yhfG-fic-pabA* | 2 | 65 | 64 | 40 | 63 | 69 | 90 | 80 | 80 | 425 | 713 | 693 | 65 | induced in stationary phase, recognized by rpoS, affects cell division |
| *yhhA* | b3448 | *yhhA* | 2 | 144 | 130 | 97 | 137 | 214 | 249 | 176 | 148 | 909 | 1740 | 1615 | 144 | hypothetical protein |
| *yhiM* | b3491 | *yhiM* | 2 | 64 | 68 | 62 | 65 | 83 | 65 | 84 | 90 | 251 | 534 | 386 | 64 | conserved inner membrane protein |
| *slp* | b3506 | *slp-dctR* | 2 | 58 | 52 | 19 | 47 | 129 | 1144 | 184 | 63 | 1655 | 3827 | 3304 | 58 | outer membrane protein induced after carbon starvation, starvation lipoprotein |
| *dctR* | b3507 | *slp-dctR* | 2 | 14 | 7 | 5 | 12 | 17 | 58 | 23 | 19 | 100 | 260 | 195 | 14 | protein involved in metabolism of C4-dicarboxylates |
| *yhiD* | b3508 | *yhiD* | 2 | 19 | 18 | 13 | 22 | 25 | 146 | 24 | 20 | 259 | 640 | 355 | 19 | putative transport ATPase |
| *hdeB* | b3509 | *hdeAB* | 2 | 198 | 165 | 70 | 130 | 416 | 3369 | 834 | 57 | 4829 | 9662 | 8379 | 198 | hypothetical protein |
| *hdeA* | b3510 | *hdeAB* | 2 | 295 | 242 | 125 | 221 | 662 | 4193 | 1164 | 117 | 6329 | 11639 | 9668 | 295 | hypothetical protein |
| *hdeD* | b3511 | *hdeD* | 2 | 70 | 54 | 32 | 72 | 80 | 995 | 119 | 51 | 1273 | 3016 | 2414 | 70 | protein involved in acid resistance |
| *gadE* | b3512 | *gadE-mdtEF* | 2 | 42 | 14 | 5 | 20 | 63 | 677 | 87 | 26 | 1206 | 5155 | 3932 | 42 | GadE transcriptional activator |
| *mdtE* | b3513 | *gadE-mdtEF* | 2 | 56 | 59 | 35 | 50 | 62 | 234 | 88 | 41 | 422 | 607 | 448 | 56 | putative membrane protein |
| *gadW* | b3515 | *gadW* | 2 | 24 | 11 | 20 | 23 | 18 | 51 | 20 | 20 | 123 | 365 | 179 | 24 | putative ARAC-type regulatory protein |
| *gadX* | b3516 | *gadAX* | 2 | 141 | 78 | 52 | 111 | 92 | 223 | 120 | 115 | 407 | 1710 | 1358 | 141 | GadX transcriptional activator |
| *gadA* | b3517 | *gadAX* | 2 | 7 | 5 | 6 | 16 | 8 | 224 | 17 | 16 | 1281 | 5035 | 4368 | 7 | glutamate decarboxylase isozyme |
| *yhjY* | b3548 | *yhjY* | 2 | 203 | 195 | 160 | 152 | 216 | 230 | 188 | 123 | 153 | 172 | 147 | 203 | putative lipase |
| *yiaG* | b3555 | *yiaG* | 2 | 16 | 14 | 13 | 25 | 19 | 24 | 23 | 30 | 999 | 2298 | 2014 | 16 | hypothetical protein |
| *aceB* | b4014 | *aceBAK* | 2 | 22 | 14 | 322 | 94 | 71 | 38 | 28 | 129 | 3733 | 4851 | 6329 | 22 | malate synthase A |
| *aceA* | b4015 | *aceBAK* | 2 | 53 | 53 | 366 | 149 | 197 | 72 | 55 | 210 | 4352 | 4912 | 5763 | 53 | isocitrate lyase |
| *aceK* | b4016 | *aceBAK* | 2 | 104 | 99 | 97 | 107 | 94 | 79 | 113 | 129 | 228 | 229 | 331 | 104 | isocitrate dehydrogenase kinase/phosphatase |
| *yjbJ* | b4045 | *yjbJ* | 2 | 151 | 163 | 120 | 188 | 199 | 239 | 279 | 253 | 4604 | 6700 | 7738 | 151 | hypothetical protein |
| *yjdN* | b4107 | *yjdN* | 2 | 27 | 25 | 37 | 26 | 43 | 36 | 32 | 32 | 621 | 1335 | 1311 | 27 | hypothetical protein |
| *yjdI* | b4126 | *yjdIJ* | 2 | 61 | 54 | 60 | 61 | 67 | 119 | 99 | 67 | 237 | 400 | 284 | 61 | hypothetical protein |
| *aidB* | b4187 | *aidB* | 2 | 8 | 11 | 7 | 13 | 12 | 22 | 11 | 20 | 382 | 939 | 1126 | 8 | putative acyl coenzyme A dehydrogenase |
| *ytfK* | b4217 | *ytfK* | 2 | 296 | 249 | 149 | 204 | 520 | 454 | 274 | 178 | 1464 | 3798 | 2179 | 296 | hypothetical protein |
| *pyrL* | b4246 | *pyrLBI* | 2 | 51 | 36 | 66 | 39 | 267 | 19 | 182 | 74 | 295 | 907 | 133 | 51 | pyrBI operon leader peptide |
| *yjiD* | b4326 | *yjiD* | 2 | 57 | 64 | 40 | 89 | 45 | 73 | 78 | 250 | 61 | 426 | 462 | 57 | hypothetical protein |
| *osmY* | b4376 | *osmY* | 2 | 31 | 38 | 50 | 75 | 64 | 54 | 39 | 137 | 2443 | 4506 | 4871 | 31 | hyperosmotically inducible periplasmic protein |
| *cpxP* | b4484 | *cpxP* | 2 | 103 | 67 | 386 | 278 | 147 | 175 | 132 | 348 | 2154 | 3598 | 2549 | 103 | regulator of the Cpx response and possible chaperone involved in resistance to extracytoplasmic stress |
| *tsx* | b0411 | *tsx* | 4 | 1434 | 1533 | 813 | 682 | 786 | 669 | 779 | 515 | 402 | 377 | 502 | 1434 | nucleoside channel receptor of phage T6 and colicin K |
| *dcuC* | b0621 | *dcuC* | 4 | 1364 | 1535 | 101 | 164 | 1101 | 1209 | 842 | 197 | 110 | 126 | 160 | 1364 | transport of dicarboxylates |
| *nagA* | b0677 | *nagBACD* | 4 | 1011 | 1145 | 527 | 391 | 342 | 483 | 307 | 213 | 334 | 361 | 307 | 1011 | N-acetylglucosamine-6-phosphate deacetylase |
| *nagB* | b0678 | *nagBACD* | 4 | 1142 | 1246 | 487 | 469 | 171 | 251 | 151 | 129 | 214 | 232 | 156 | 1142 | glucosamine-6-phosphate deaminase |
| *nagE* | b0679 | *nagE* | 4 | 983 | 1047 | 640 | 473 | 363 | 342 | 260 | 221 | 176 | 153 | 176 | 983 | PTS system, N-acetylglucosamine-specific enzyme IIABC |
| *galM* | b0756 | *galETKM* | 4 | 917 | 1269 | 967 | 865 | 375 | 665 | 387 | 487 | 310 | 346 | 275 | 917 | galactose-1-epimerase (mutarotase) |
| *galK* | b0757 | *galETKM* | 4 | 1354 | 1836 | 1178 | 1284 | 696 | 974 | 598 | 841 | 510 | 582 | 511 | 1354 | galactokinase |
| *galT* | b0758 | *galETKM* | 4 | 1200 | 1828 | 756 | 772 | 424 | 477 | 327 | 533 | 142 | 119 | 118 | 1200 | galactose-1-phosphate uridylyltransferase |
| *galE* | b0759 | *galETKM* | 4 | 1809 | 2206 | 918 | 1311 | 636 | 698 | 549 | 1020 | 208 | 150 | 224 | 1809 | UDP-galactose-4-epimerase |
| *dmsA* | b0894 | *dmsABC* | 4 | 778 | 1237 | 44 | 90 | 502 | 993 | 858 | 72 | 36 | 41 | 53 | 778 | anaerobic dimethyl sulfoxide reductase subunit A |
| *dmsB* | b0895 | *dmsABC* | 4 | 718 | 1151 | 36 | 85 | 332 | 919 | 750 | 74 | 46 | 102 | 79 | 718 | anaerobic dimethyl sulfoxide reductase subunit B |
| *dhaM* | b1198 | *dhaKLM* | 4 | 636 | 974 | 653 | 123 | 228 | 212 | 127 | 81 | 121 | 104 | 72 | 636 | dihydroxyacetone kinase subunit M |
| *dhaL* | b1199 | *dhaKLM* | 4 | 914 | 1349 | 1024 | 335 | 550 | 466 | 344 | 312 | 366 | 279 | 415 | 914 | putative dihydroxyacetone kinase (EC 2.7.1.2) |
| *dhaK* | b1200 | *dhaKLM* | 4 | 838 | 1335 | 1106 | 251 | 531 | 503 | 274 | 207 | 320 | 252 | 160 | 838 | putative dihydroxyacetone kinase (EC 2.7.1.2) |
| *narK* | b1223 | *narK* | 4 | 1229 | 1312 | 68 | 197 | 86 | 252 | 157 | 276 | 80 | 84 | 118 | 1229 | nitrite extrusion protein |
| *narG* | b1224 | *narGHJI* | 4 | 1510 | 1915 | 117 | 288 | 134 | 264 | 265 | 241 | 128 | 152 | 245 | 1510 | nitrate reductase 1, alpha subunit |
| *narH* | b1225 | *narGHJI* | 4 | 1007 | 1464 | 70 | 124 | 41 | 147 | 131 | 99 | 45 | 51 | 64 | 1007 | nitrate reductase 1, beta subunit |
| *narJ* | b1226 | *narGHJI* | 4 | 556 | 807 | 75 | 101 | 76 | 138 | 137 | 112 | 77 | 82 | 103 | 556 | nitrate reductase 1, delta subunit, assembly function |
| *narI* | b1227 | *narGHJI* | 4 | 180 | 231 | 54 | 71 | 61 | 74 | 85 | 74 | 35 | 34 | 80 | 180 | nitrate reductase 1, cytochrome b(NR), gamma subunit |
| *adhE* | b1241 | *adhE* | 4 | 1917 | 1829 | 319 | 297 | 1650 | 1861 | 1516 | 245 | 1623 | 2031 | 1581 | 1917 | CoA-linked acetaldehyde dehydrogenase and iron-dependent alcohol dehydrogenase pyruvate-formate-lyase deactivase |
| *ydeN* | b1498 | *ydeN* | 4 | 801 | 887 | 797 | 278 | 303 | 847 | 421 | 48 | 41 | 30 | 26 | 801 | putative sulfatase |
| *napB* | b2203 | *napFDAGHBC-ccmABCDEFGH* | 4 | 261 | 423 | 153 | 132 | 130 | 175 | 141 | 262 | 102 | 102 | 108 | 261 | cytochrome c-type protein |
| *napD* | b2207 | *napFDAGHBC-ccmABCDEFGH* | 4 | 847 | 649 | 88 | 206 | 87 | 155 | 149 | 473 | 75 | 88 | 82 | 847 | hypothetical protein |
| *napF* | b2208 | *napFDAGHBC-ccmABCDEFGH* | 4 | 680 | 449 | 32 | 140 | 59 | 102 | 75 | 385 | 29 | 30 | 22 | 680 | ferredoxin-type protein: electron transfer |
| *dsdX* | b2365 | *dsdXA* | 4 | 474 | 787 | 45 | 305 | 34 | 32 | 26 | 22 | 26 | 24 | 29 | 474 | transport system permease (serine?) |
| *dsdA* | b2366 | *dsdXA* | 4 | 611 | 785 | 235 | 643 | 223 | 260 | 219 | 257 | 195 | 166 | 279 | 611 | D-serine dehydratase (deaminase) |
| *yfeX* | b2431 | *yfeYX* | 4 | 608 | 756 | 206 | 260 | 313 | 525 | 486 | 226 | 188 | 188 | 141 | 608 | hypothetical protein |
| *proX* | b2679 | *proVWX* | 4 | 321 | 274 | 440 | 46 | 32 | 533 | 203 | 36 | 61 | 40 | 39 | 321 | high-affinity transport system for glycine betaine and proline |
| *srlA* | b2702 | *srlAEBD-gutM-srlR-gutQ* | 4 | 2070 | 1819 | 1396 | 364 | 1082 | 990 | 436 | 140 | 96 | 102 | 95 | 2070 | PTS system, glucitol/sorbitol-specific IIC component, one of two |
| *srlE* | b2703 | *srlAEBD-gutM-srlR-gutQ* | 4 | 907 | 812 | 791 | 84 | 206 | 314 | 131 | 26 | 14 | 19 | 10 | 907 | PTS system, glucitol/sorbitol-specific IIB component and second of two IIC components |
| *srlB* | b2704 | *srlAEBD-gutM-srlR-gutQ* | 4 | 1011 | 986 | 873 | 140 | 191 | 367 | 206 | 72 | 69 | 66 | 75 | 1011 | PTS system, glucitol/sorbitol-specific enzyme IIA component |
| *srlD* | b2705 | *srlAEBD-gutM-srlR-gutQ* | 4 | 1235 | 1245 | 965 | 146 | 170 | 363 | 232 | 71 | 61 | 67 | 60 | 1235 | glucitol (sorbitol)-6-phosphate dehydrogenase |
| *gutM* | b2706 | *srlAEBD-gutM-srlR-gutQ* | 4 | 381 | 554 | 371 | 44 | 68 | 121 | 54 | 25 | 16 | 19 | 29 | 381 | glucitol operon activator |
| *gutQ* | b2708 | *srlAEBD-gutM-srlR-gutQ* | 4 | 528 | 501 | 409 | 431 | 419 | 465 | 413 | 307 | 250 | 225 | 253 | 528 | hypothetical protein |
| *hypA* | b2726 | *hypABCDE-fhlA* | 4 | 1608 | 1532 | 178 | 523 | 1611 | 1803 | 1881 | 1043 | 142 | 151 | 156 | 1608 | pleiotrophic effects on 3 hydrogenase isozymes |
| *hypB* | b2727 | *hypABCDE-fhlA* | 4 | 1233 | 1577 | 126 | 263 | 1117 | 1315 | 1274 | 527 | 46 | 49 | 42 | 1233 | guanine-nucleotide binding protein, functions as nickel donor for large subunit of hydrogenase 3 |
| *hypC* | b2728 | *hypABCDE-fhlA* | 4 | 1107 | 1259 | 189 | 264 | 871 | 1029 | 1078 | 298 | 108 | 99 | 160 | 1107 | pleiotrophic effects on 3 hydrogenase isozymes |
| *hypD* | b2729 | *hypABCDE-fhlA* | 4 | 738 | 1196 | 142 | 186 | 401 | 607 | 603 | 163 | 107 | 183 | 160 | 738 | pleiotrophic effects on 3 hydrogenase isozymes |
| *galP* | b2943 | *galP* | 4 | 919 | 1511 | 258 | 164 | 17 | 48 | 29 | 44 | 14 | 23 | 45 | 919 | galactose-proton symport of transport system |
| *garP* | b3127 | *garPLRK-rnpB* | 4 | 457 | 392 | 159 | 284 | 205 | 87 | 48 | 68 | 37 | 29 | 30 | 457 | putative transport protein |
| *garD* | b3128 | *garD* | 4 | 676 | 620 | 213 | 326 | 346 | 223 | 176 | 133 | 95 | 78 | 117 | 676 | galactarate dehydratase |
| *deaD* | b3162 | *deaD* | 4 | 680 | 741 | 291 | 598 | 321 | 478 | 465 | 472 | 310 | 267 | 526 | 680 | inducible ATP-independent RNA helicase |
| *nanA* | b3225 | *nanATEK-yhcH* | 4 | 1649 | 1773 | 1608 | 1193 | 102 | 100 | 96 | 97 | 59 | 55 | 54 | 1649 | N-acetylneuraminate lyase (aldolase) catabolism of sialic acid not K-12? |
| *nirB* | b3365 | *nirBDC-cysG* | 4 | 1725 | 1871 | 43 | 124 | 152 | 90 | 145 | 82 | 46 | 60 | 58 | 1725 | nitrite reductase (NAD(P)H) subunit |
| *nirD* | b3366 | *nirBDC-cysG* | 4 | 1385 | 1769 | 70 | 93 | 134 | 109 | 134 | 67 | 37 | 47 | 65 | 1385 | nitrite reductase (NAD(P)H) subunit |
| *nirC* | b3367 | *nirBDC-cysG* | 4 | 283 | 511 | 19 | 33 | 15 | 43 | 26 | 29 | 16 | 12 | 8 | 283 | nitrite reductase activity |
| *feoB* | b3409 | *feoAB* | 4 | 572 | 507 | 330 | 404 | 429 | 398 | 317 | 237 | 229 | 167 | 109 | 572 | ferrous iron transport protein B |
| *yhjX* | b3547 | *yhjX* | 4 | 81 | 9 | 7 | 15 | 15 | 6 | 7 | 11 | 10 | 11 | 20 | 81 | putative resistance protein |
| *cspA* | b3556 | *cspA* | 4 | 3452 | 2639 | 991 | 2961 | 944 | 956 | 896 | 560 | 391 | 129 | 133 | 3452 | cold shock protein 7.4, transcriptional activator of hns |
| *ysaA* | b3573 | *ysaA* | 4 | 382 | 332 | 26 | 46 | 262 | 235 | 119 | 121 | 26 | 28 | 28 | 382 | hypothetical protein |
| *gpmM* | b3612 | *gpmM-envC-yibQ* | 4 | 1350 | 1309 | 234 | 391 | 1266 | 1031 | 1435 | 369 | 160 | 152 | 138 | 1350 | putative 2,3-bisphosphoglycerate-independent phosphoglycerate mutase |
| *udp* | b3831 | *udp* | 4 | 1002 | 1179 | 491 | 586 | 594 | 767 | 621 | 564 | 518 | 542 | 801 | 1002 | uridine phosphorylase |
| *spf* | b3864 | *spf* | 4 | 1587 | 1197 | 289 | 1176 | 477 | 750 | 1102 | 484 | 290 | 467 | 381 | 1587 |  |
| *pfkA* | b3916 | *pfkA* | 4 | 1483 | 1659 | 344 | 443 | 886 | 1004 | 1088 | 399 | 453 | 529 | 536 | 1483 | 6-phosphofructokinase I |
| *nrfA* | b4070 | *nrfABCDEFG* | 4 | 729 | 1065 | 85 | 66 | 69 | 85 | 244 | 89 | 56 | 70 | 96 | 729 | periplasmic cytochrome c(552): plays a role in nitrite reduction |
| *nrfB* | b4071 | *nrfABCDEFG* | 4 | 430 | 850 | 48 | 31 | 32 | 47 | 158 | 44 | 24 | 38 | 49 | 430 | formate-dependent nitrite reductase a penta-haeme cytochrome c |
| *nrfC* | b4072 | *nrfABCDEFG* | 4 | 176 | 270 | 82 | 108 | 97 | 103 | 130 | 101 | 113 | 108 | 131 | 176 | formate-dependent nitrite reductase Fe-S centers |
| *yjdK* | b4128 | *yjdKO* | 4 | 242 | 353 | 6 | 73 | 83 | 192 | 98 | 72 | 17 | 32 | 22 | 242 | hypothetical protein |
| *purA* | b4177 | *purA* | 4 | 768 | 879 | 474 | 371 | 407 | 528 | 298 | 162 | 332 | 139 | 98 | 768 | adenylosuccinate synthetase |
| *yjiX* | b4353 | *yjiXA* | 4 | 852 | 1198 | 535 | 478 | 414 | 663 | 457 | 302 | 213 | 303 | 193 | 852 | hypothetical protein |
| *yjiY* | b4354 | *yjiY* | 4 | 1203 | 1983 | 91 | 424 | 49 | 70 | 57 | 49 | 64 | 91 | 65 | 1203 | putative carbon starvation protein |
| *yjjW* | b4379 | *yjjW* | 4 | 323 | 433 | 176 | 201 | 288 | 355 | 398 | 225 | 223 | 224 | 348 | 323 | putative activating enzyme |
| *yjjI* | b4380 | *yjjI* | 4 | 882 | 1165 | 52 | 216 | 440 | 785 | 747 | 129 | 52 | 73 | 75 | 882 | hypothetical protein |
| *deoB* | b4383 | *deoCABD* | 4 | 971 | 1438 | 525 | 483 | 317 | 507 | 427 | 240 | 742 | 1206 | 1051 | 971 | phosphopentomutase |
| *dnaK* | b0014 | *dnaK-tpke11-dnaJ* | 5 | 465 | 335 | 2281 | 1706 | 1912 | 1518 | 1200 | 1155 | 1861 | 3221 | 4074 | 465 | chaperone Hsp70 DNA biosynthesis autoregulated heat shock proteins |
| *dnaJ* | b0015 | *dnaK-tpke11-dnaJ* | 5 | 324 | 251 | 1110 | 840 | 873 | 885 | 528 | 379 | 1092 | 1753 | 1736 | 324 | chaperone with DnaK heat shock protein |
| *can* | b0126 | *can* | 5 | 533 | 490 | 1234 | 991 | 527 | 640 | 485 | 610 | 569 | 699 | 824 | 533 | putative carbonic anhdrase (EC 4.2.1.1) |
| *cyoD* | b0429 | *cyoABCDE* | 5 | 1163 | 915 | 1953 | 1841 | 2558 | 140 | 106 | 1296 | 1443 | 930 | 574 | 1163 | cytochrome o ubiquinol oxidase subunit IV |
| *cyoC* | b0430 | *cyoABCDE* | 5 | 970 | 811 | 1808 | 1648 | 2114 | 145 | 143 | 1254 | 1060 | 651 | 509 | 970 | cytochrome o ubiquinol oxidase subunit III |
| *cyoB* | b0431 | *cyoABCDE* | 5 | 1220 | 1023 | 2199 | 2082 | 2404 | 200 | 205 | 1566 | 1435 | 747 | 647 | 1220 | cytochrome o ubiquinol oxidase subunit I |
| *cyoA* | b0432 | *cyoABCDE* | 5 | 1131 | 948 | 2645 | 2238 | 1848 | 209 | 306 | 1549 | 1939 | 1370 | 1220 | 1131 | cytochrome o ubiquinol oxidase subunit II |
| *glnW* | b0668 | *metT-leuW-glnUW-metU-glnVX* | 5 | 1155 | 1060 | 1313 | 1309 | 1140 | 781 | 1164 | 1239 | 1259 | 1377 | 1597 | 1155 |  |
| *gltA* | b0720 | *gltA* | 5 | 730 | 516 | 3314 | 2073 | 4077 | 1499 | 859 | 1305 | 3211 | 2112 | 1539 | 730 | citrate synthase |
| *sdhC* | b0721 | *sdhCDAB-b0725-sucABCD* | 5 | 230 | 175 | 1782 | 1416 | 727 | 125 | 168 | 923 | 872 | 813 | 253 | 230 | succinate dehydrogenase, cytochrome b556 |
| *sdhD* | b0722 | *sdhCDAB-b0725-sucABCD* | 5 | 407 | 301 | 2415 | 1908 | 1160 | 237 | 323 | 1391 | 1284 | 999 | 446 | 407 | succinate dehydrogenase, hydrophobic subunit |
| *sdhA* | b0723 | *sdhCDAB-b0725-sucABCD* | 5 | 667 | 549 | 3383 | 1988 | 2536 | 553 | 497 | 1664 | 1700 | 1206 | 737 | 667 | succinate dehydrogenase, flavoprotein subunit |
| *sdhB* | b0724 | *sdhCDAB-b0725-sucABCD* | 5 | 246 | 190 | 1426 | 1148 | 1101 | 185 | 145 | 907 | 690 | 337 | 139 | 246 | succinate dehydrogenase, iron sulfur protein |
| *b0725* | b0725 | *sdhCDAB-b0725-sucABCD* | 5 | 614 | 391 | 1990 | 1609 | 1714 | 426 | 242 | 1039 | 1217 | 866 | 343 | 614 | hypothetical protein |
| *sucA* | b0726 | *sdhCDAB-b0725-sucABCD* | 5 | 958 | 689 | 2068 | 2249 | 2245 | 640 | 370 | 1401 | 1558 | 852 | 350 | 958 | 2-oxoglutarate dehydrogenase (decarboxylase component) |
| *sucB* | b0727 | *sdhCDAB-b0725-sucABCD* | 5 | 1637 | 1269 | 3382 | 3084 | 4601 | 1126 | 771 | 1885 | 2110 | 1444 | 648 | 1637 | 2-oxoglutarate dehydrogenase (dihydrolipoyltranssuccinase E2 component) |
| *sucC* | b0728 | *sdhCDAB-b0725-sucABCD* | 5 | 1765 | 1277 | 4326 | 3575 | 6211 | 1325 | 941 | 2336 | 3163 | 2619 | 1227 | 1765 | succinyl-CoA synthetase, beta subunit |
| *sucD* | b0729 | *sdhCDAB-b0725-sucABCD* | 5 | 724 | 448 | 1387 | 1479 | 2649 | 405 | 324 | 984 | 1113 | 854 | 355 | 724 | succinyl-CoA synthetase, alpha subunit |
| *putP* | b1015 | *putP* | 5 | 381 | 326 | 614 | 726 | 453 | 446 | 428 | 464 | 688 | 602 | 744 | 381 | major sodium/proline symporter |
| *rnb* | b1286 | *rnb* | 5 | 121 | 115 | 374 | 415 | 103 | 125 | 151 | 438 | 162 | 224 | 237 | 121 | RNase II, mRNA degradation |
| *paaE* | b1392 | *paaABCDEFGHIJK* | 5 | 13 | 12 | 128 | 21 | 19 | 16 | 18 | 28 | 28 | 27 | 38 | 13 | putative oxidoreductase |
| *paaF* | b1393 | *paaABCDEFGHIJK* | 5 | 6 | 9 | 106 | 13 | 17 | 10 | 10 | 19 | 17 | 22 | 18 | 6 | putative enzyme |
| *ydcI* | b1422 | *ydcI* | 5 | 19 | 21 | 384 | 80 | 31 | 8 | 15 | 33 | 150 | 98 | 90 | 19 | putative transcriptional regulator LYSR-type |
| *fumA* | b1612 | *fumA* | 5 | 317 | 224 | 1528 | 1165 | 858 | 207 | 228 | 800 | 848 | 462 | 392 | 317 | fumarase A = fumarate hydratase Class I aerobic isozyme |
| *pps* | b1702 | *pps* | 5 | 534 | 479 | 740 | 1093 | 721 | 622 | 948 | 994 | 980 | 716 | 1351 | 534 | phosphoenolpyruvate synthase |
| *fliY* | b1920 | *fliAZY* | 5 | 540 | 473 | 1014 | 1042 | 864 | 736 | 768 | 1500 | 1092 | 1125 | 1451 | 540 | putative periplasmic binding transport protein |
| *yedE* | b1929 | *yedEF* | 5 | 98 | 82 | 163 | 148 | 373 | 166 | 214 | 477 | 138 | 108 | 85 | 98 | putative transport system permease protein |
| *yeeD* | b2012 | *yeeED* | 5 | 272 | 135 | 636 | 466 | 341 | 375 | 567 | 1008 | 385 | 468 | 809 | 272 | hypothetical protein |
| *yeeE* | b2013 | *yeeED* | 5 | 177 | 158 | 453 | 346 | 202 | 235 | 256 | 512 | 364 | 372 | 448 | 177 | putative transport system permease protein |
| *mglC* | b2148 | *mglBAC* | 5 | 94 | 103 | 1002 | 495 | 514 | 102 | 78 | 270 | 129 | 78 | 50 | 94 | methyl-galactoside transport and galactose taxis |
| *mglA* | b2149 | *mglBAC* | 5 | 111 | 147 | 1886 | 901 | 954 | 114 | 123 | 576 | 334 | 173 | 165 | 111 | ATP-binding component of methyl-galactoside transport and galactose taxis |
| *mglB* | b2150 | *mglBAC* | 5 | 271 | 304 | 4653 | 1943 | 2750 | 271 | 269 | 1257 | 1388 | 595 | 334 | 271 | galactose-binding transport protein receptor for galactose taxis |
| *cysK* | b2414 | *cysK* | 5 | 1056 | 464 | 1804 | 1784 | 1305 | 1969 | 1861 | 2260 | 1416 | 3174 | 4159 | 1056 | cysteine synthase A, O-acetylserine sulfhydrolase A |
| *cysA* | b2422 | *cysPUWAM* | 5 | 252 | 232 | 382 | 339 | 324 | 252 | 349 | 610 | 192 | 506 | 682 | 252 | ATP-binding component of sulfate permease A protein chromate resistance |
| *cysU* | b2424 | *cysPUWAM* | 5 | 247 | 287 | 513 | 485 | 260 | 261 | 236 | 632 | 408 | 754 | 1195 | 247 | sulfate, thiosulfate transport system permease T protein |
| *cysP* | b2425 | *cysPUWAM* | 5 | 77 | 66 | 863 | 678 | 94 | 84 | 173 | 1026 | 891 | 1053 | 1519 | 77 | thiosulfate binding protein |
| *maeB* | b2463 | *maeB* | 5 | 339 | 298 | 1266 | 1059 | 962 | 400 | 297 | 895 | 707 | 287 | 257 | 339 | malate dehydrogenase (oxaloacetate-decarboxylating) (NADP+) |
| *ndk* | b2518 | *ndk* | 5 | 146 | 148 | 1433 | 1557 | 279 | 122 | 186 | 1196 | 330 | 383 | 305 | 146 | nucleoside diphosphate kinase |
| *iscS* | b2530 | *iscRSUA* | 5 | 755 | 534 | 1321 | 909 | 1242 | 1119 | 414 | 430 | 1380 | 2374 | 1440 | 755 | putative aminotransferase |
| *clpB* | b2592 | *clpB* | 5 | 213 | 128 | 883 | 385 | 826 | 774 | 486 | 294 | 647 | 1149 | 837 | 213 | heat shock protein |
| *proV* | b2677 | *proVWX* | 5 | 113 | 89 | 709 | 92 | 34 | 232 | 90 | 53 | 86 | 73 | 56 | 113 | ATP-binding component of transport system for glycine, betaine and proline |
| *proW* | b2678 | *proVWX* | 5 | 142 | 145 | 458 | 126 | 78 | 195 | 86 | 83 | 93 | 57 | 22 | 142 | high-affinity transport system for glycine betaine and proline |
| *cysC* | b2750 | *cysDNC* | 5 | 111 | 44 | 428 | 224 | 130 | 95 | 226 | 636 | 90 | 401 | 732 | 111 | adenosine 5'-phosphosulfate kinase |
| *cysN* | b2751 | *cysDNC* | 5 | 150 | 43 | 616 | 294 | 213 | 164 | 303 | 926 | 151 | 663 | 1273 | 150 | ATP-sulfurylase (ATP:sulfate adenylyltransferase), subunit 1, probably a GTPase |
| *cysH* | b2762 | *cysJIH* | 5 | 293 | 60 | 560 | 543 | 399 | 337 | 581 | 1180 | 156 | 778 | 1003 | 293 | 3'-phosphoadenosine 5'-phosphosulfate reductase |
| *cysI* | b2763 | *cysJIH* | 5 | 299 | 131 | 659 | 678 | 360 | 388 | 578 | 1163 | 255 | 886 | 1237 | 299 | sulfite reductase, alpha subunit |
| *cysJ* | b2764 | *cysJIH* | 5 | 220 | 208 | 878 | 790 | 246 | 229 | 348 | 1152 | 380 | 916 | 1505 | 220 | sulfite reductase (NADPH), flavoprotein beta subunit |
| *sdaC* | b2796 | *sdaCB* | 5 | 819 | 751 | 1114 | 1668 | 572 | 359 | 309 | 1249 | 380 | 376 | 237 | 819 | probable serine transporter |
| *yqgB* | b2939 | *yqgB* | 5 | 210 | 129 | 349 | 391 | 141 | 152 | 129 | 239 | 88 | 63 | 48 | 210 | hypothetical protein |
| *yhcH* | b3221 | *nanATEK-yhcH* | 5 | 489 | 684 | 1071 | 688 | 104 | 91 | 77 | 64 | 84 | 166 | 81 | 489 | hypothetical protein |
| *nanK* | b3222 | *nanATEK-yhcH* | 5 | 340 | 501 | 976 | 512 | 124 | 107 | 122 | 127 | 77 | 186 | 179 | 340 | putative NAGC-like transcriptional regulator |
| *nanT* | b3224 | *nanATEK-yhcH* | 5 | 569 | 694 | 1261 | 632 | 166 | 177 | 145 | 140 | 100 | 90 | 110 | 569 | sialic acid transporter |
| *dctA* | b3528 | *dctA* | 5 | 168 | 162 | 1423 | 1126 | 282 | 93 | 108 | 294 | 386 | 238 | 188 | 168 | uptake of C4-dicarboxylic acids |
| *lldP* | b3603 | *lldPRD* | 5 | 117 | 120 | 3273 | 1470 | 63 | 50 | 45 | 76 | 362 | 253 | 200 | 117 | L-lactate permease |
| *lldR* | b3604 | *lldPRD* | 5 | 66 | 55 | 1496 | 1020 | 22 | 8 | 6 | 25 | 200 | 122 | 112 | 66 | transcriptional regulator |
| *lldD* | b3605 | *lldPRD* | 5 | 305 | 235 | 2023 | 1676 | 100 | 68 | 72 | 100 | 384 | 317 | 276 | 305 | L-lactate dehydrogenase |
| *uhpT* | b3666 | *uhpT* | 5 | 62 | 74 | 54 | 130 | 87 | 66 | 63 | 346 | 56 | 69 | 89 | 62 | hexose phosphate transport protein |
| *ibpB* | b3686 | *ibpAB* | 5 | 39 | 42 | 1972 | 236 | 46 | 45 | 35 | 228 | 50 | 108 | 74 | 39 | heat shock protein |
| *ibpA* | b3687 | *ibpAB* | 5 | 50 | 37 | 3044 | 447 | 90 | 99 | 55 | 562 | 170 | 299 | 221 | 50 | heat shock protein |
| *atpB* | b3738 | *atpIBEFHAGDC* | 5 | 916 | 829 | 1686 | 1795 | 1269 | 1344 | 1171 | 1118 | 1048 | 924 | 754 | 916 | membrane-bound ATP synthase, F0 sector, subunit a |
| *sbp* | b3917 | *sbp* | 5 | 432 | 402 | 368 | 565 | 441 | 464 | 636 | 1024 | 411 | 495 | 1498 | 432 | periplasmic sulfate-binding protein |
| *hslU* | b3931 | *hslVU* | 5 | 265 | 217 | 1426 | 909 | 439 | 498 | 316 | 336 | 564 | 692 | 442 | 265 | heat shock protein hslVU, ATPase subunit, homologous to chaperones |
| *hslV* | b3932 | *hslVU* | 5 | 266 | 186 | 1861 | 921 | 420 | 493 | 313 | 431 | 605 | 1068 | 773 | 266 | heat shock protein hslVU, proteasome-related peptidase subunit |
| *sthA* | b3962 | *sthA* | 5 | 273 | 262 | 1147 | 582 | 497 | 209 | 252 | 463 | 579 | 370 | 298 | 273 | putative oxidoreductase |
| *tyrU* | b3977 | *thrU-tyrU-glyT-thrT-tufB* | 5 | 1272 | 1200 | 2381 | 1741 | 1197 | 824 | 1503 | 1515 | 1024 | 1304 | 1692 | 1272 |  |
| *groS* | b4142 | *groSL* | 5 | 1588 | 1204 | 7911 | 3725 | 4928 | 2985 | 3179 | 3034 | 3131 | 7494 | 6658 | 1588 | GroES, 10 Kd chaperone binds to Hsp60 in pres. Mg-ATP, suppressing its ATPase activity |
| *groL* | b4143 | *groSL* | 5 | 795 | 646 | 3260 | 2089 | 2776 | 2041 | 1865 | 1230 | 1812 | 3475 | 3401 | 795 | GroEL, chaperone Hsp60, peptide-dependent ATPase, heat shock protein |
| *yjhS* | b4309 | *nanC-yjhT-yjhS* | 5 | 41 | 62 | 214 | 34 | 28 | 31 | 23 | 21 | 34 | 40 | 41 | 41 | hypothetical protein |
| *yjhT* | b4310 | *nanC-yjhT-yjhS* | 5 | 202 | 265 | 909 | 548 | 32 | 27 | 40 | 47 | 65 | 74 | 70 | 202 | hypothetical protein |
| *nanC* | b4311 | *nanC-yjhT-yjhS* | 5 | 307 | 375 | 1083 | 754 | 21 | 20 | 17 | 29 | 15 | 23 | 17 | 307 | hypothetical protein |
| *fimA* | b4314 | *fimAICDFGH* | 5 | 718 | 584 | 315 | 1618 | 1230 | 1005 | 418 | 2075 | 253 | 153 | 184 | 718 | major type 1 subunit fimbrin (pilin) |
| *fimI* | b4315 | *fimAICDFGH* | 5 | 117 | 89 | 45 | 467 | 108 | 100 | 65 | 468 | 54 | 52 | 121 | 117 | fimbrial protein |
| *fimC* | b4316 | *fimAICDFGH* | 5 | 137 | 130 | 200 | 356 | 250 | 173 | 163 | 380 | 80 | 60 | 59 | 137 | periplasmic chaperone, required for type 1 fimbriae |
| *ykgM* | b0296 | *ykgMO* | 6 | 29 | 34 | 38 | 71 | 28 | 30 | 27 | 104 | 32 | 29 | 31 | 29 | putative ribosomal protein |
| *sulA* | b0958 | *sulA* | 6 | 161 | 154 | 331 | 2024 | 246 | 314 | 386 | 1145 | 193 | 419 | 315 | 161 | suppressor of lon inhibits cell division and ftsZ ring formation |
| *cspG* | b0990 | *cspG* | 6 | 256 | 231 | 50 | 502 | 66 | 59 | 68 | 90 | 49 | 45 | 22 | 256 | homolog of Salmonella cold shock protein |
| *dinI* | b1061 | *dinI* | 6 | 185 | 132 | 239 | 1239 | 195 | 205 | 279 | 800 | 96 | 127 | 137 | 185 | damage-inducible protein I |
| *xisE* | b1141 | *ymfH-xisE-intE* | 6 | 13 | 6 | 34 | 363 | 23 | 45 | 62 | 188 | 22 | 38 | 39 | 13 | hypothetical protein |
| *ymfJ* | b1144 | *ymfJ* | 6 | 15 | 22 | 45 | 547 | 30 | 83 | 89 | 237 | 33 | 84 | 94 | 15 | hypothetical protein |
| *ymfL* | b1147 | *ymfTLMNROPQ-ycfK-ymfS* | 6 | 20 | 23 | 32 | 194 | 23 | 49 | 43 | 81 | 24 | 34 | 49 | 20 | hypothetical protein |
| *umuD* | b1183 | *umuDC* | 6 | 105 | 105 | 73 | 308 | 91 | 93 | 110 | 161 | 115 | 138 | 136 | 105 | SOS mutagenesis error-prone repair processed to UmuD' forms complex with UmuC |
| *yebG* | b1848 | *yebG* | 6 | 351 | 319 | 257 | 994 | 323 | 444 | 401 | 1042 | 263 | 280 | 185 | 351 | hypothetical protein |
| *nrdE* | b2675 | *nrdHIEF* | 6 | 41 | 30 | 156 | 202 | 33 | 37 | 54 | 71 | 116 | 165 | 211 | 41 | ribonucleoside-diphosphate reductase 2, alpha subunit |
| *recX* | b2698 | *recAX* | 6 | 43 | 44 | 55 | 174 | 35 | 51 | 42 | 58 | 36 | 47 | 52 | 43 | regulator, OraA protein |
| *recA* | b2699 | *recAX* | 6 | 859 | 821 | 873 | 3222 | 849 | 967 | 1262 | 1392 | 513 | 520 | 603 | 859 | DNA strand exchange and renaturation, DNA-dependent ATPase, DNA- and ATP-dependent coprotease |
| *mqsR* | b3022 | *mqsR-ygiT* | 6 | 65 | 39 | 72 | 453 | 70 | 89 | 70 | 271 | 169 | 256 | 143 | 65 | motility quorum-sensing regulator |
| *bglG* | b3723 | *bglGFB* | 6 | 64 | 78 | 53 | 77 | 66 | 67 | 64 | 62 | 51 | 55 | 55 | 64 | positive regulation of bgl operon |
| *sodA* | b3908 | *sodA* | 6 | 1006 | 1034 | 1451 | 1971 | 481 | 119 | 110 | 490 | 1846 | 2211 | 2145 | 1006 | superoxide dismutase, manganese |
| *fsaB* | b3946 | *ptsA-fsaB-gldA* | 6 | 96 | 138 | 78 | 158 | 47 | 82 | 103 | 65 | 53 | 65 | 62 | 96 | putative transaldolase |
| *fecD* | b4288 | *fecABCDE* | 6 | 176 | 193 | 155 | 180 | 111 | 62 | 84 | 124 | 97 | 87 | 138 | 176 | citrate-dependent iron transport, membrane-bound protein |
| *fecB* | b4290 | *fecABCDE* | 6 | 244 | 152 | 277 | 349 | 71 | 41 | 43 | 73 | 213 | 107 | 87 | 244 | citrate-dependent iron transport, periplasmic protein |
| *fecA* | b4291 | *fecABCDE* | 6 | 312 | 186 | 611 | 814 | 105 | 62 | 100 | 174 | 483 | 286 | 296 | 312 | outer membrane receptor citrate-dependent iron transport, outer membrane receptor |
| *rihC* | b0030 | *rihC* | 7 | 414 | 690 | 681 | 338 | 656 | 777 | 532 | 256 | 175 | 128 | 154 | 414 | ribonucleoside hydrolase 3 |
| *carB* | b0033 | *carAB* | 7 | 166 | 131 | 257 | 120 | 3012 | 336 | 1498 | 1380 | 170 | 100 | 145 | 166 | carbamoyl-phosphate synthase large subunit |
| *aceE* | b0114 | *pdhR-aceEF-lpd* | 7 | 1815 | 1895 | 1011 | 1603 | 4178 | 4566 | 4222 | 1229 | 757 | 650 | 614 | 1815 | pyruvate dehydrogenase (decarboxylase component) |
| *aceF* | b0115 | *pdhR-aceEF-lpd* | 7 | 1343 | 1611 | 784 | 1075 | 2899 | 3548 | 3358 | 979 | 546 | 402 | 293 | 1343 | pyruvate dehydrogenase (dihydrolipoyltransacetylase component) |
| *ykgE* | b0306 | *ykgEFG* | 7 | 1286 | 1989 | 278 | 321 | 1551 | 2847 | 1615 | 137 | 94 | 133 | 129 | 1286 | putative dehydrogenase subunit |
| *ykgF* | b0307 | *ykgEFG* | 7 | 281 | 737 | 106 | 75 | 406 | 775 | 401 | 49 | 40 | 45 | 62 | 281 | hypothetical protein |
| *ybcW* | b0559 | *ybcW* | 7 | 43 | 38 | 22 | 56 | 31 | 99 | 43 | 20 | 14 | 18 | 23 | 43 | hypothetical protein |
| *rihA* | b0651 | *rihA* | 7 | 657 | 978 | 695 | 494 | 848 | 957 | 859 | 470 | 166 | 84 | 90 | 657 | putative tRNA synthetase |
| *asnB* | b0674 | *asnB* | 7 | 223 | 223 | 216 | 261 | 320 | 1897 | 228 | 274 | 282 | 269 | 368 | 223 | asparagine synthetase B |
| *cydA* | b0733 | *cydAB* | 7 | 1825 | 1863 | 454 | 550 | 2758 | 3616 | 3272 | 409 | 696 | 410 | 325 | 1825 | cytochrome d terminal oxidase, polypeptide subunit I |
| *cydB* | b0734 | *cydAB* | 7 | 2011 | 2014 | 546 | 643 | 2340 | 3751 | 3415 | 403 | 747 | 516 | 388 | 2011 | cytochrome d terminal oxidase polypeptide subunit II |
| *ybgE* | b0735 | *ybgE* | 7 | 498 | 439 | 108 | 131 | 394 | 1006 | 705 | 110 | 163 | 156 | 108 | 498 | hypothetical protein |
| *pflB* | b0903 | *focA-pflB* | 7 | 2410 | 1943 | 674 | 866 | 2134 | 6815 | 4673 | 555 | 1983 | 2380 | 1855 | 2410 | formate acetyltransferase 1 |
| *ycbJ* | b0919 | *ycbJ* | 7 | 649 | 772 | 124 | 170 | 779 | 895 | 613 | 359 | 179 | 172 | 167 | 649 | hypothetical protein |
| *flgB* | b1073 | *flgBCDEFGHIJ* | 7 | 455 | 52 | 59 | 46 | 686 | 391 | 290 | 198 | 15 | 12 | 25 | 455 | flagellar biosynthesis, cell-proximal portion of basal-body rod |
| *flgC* | b1074 | *flgBCDEFGHIJ* | 7 | 745 | 169 | 118 | 68 | 1221 | 681 | 520 | 315 | 21 | 21 | 37 | 745 | flagellar biosynthesis, cell-proximal portion of basal-body rod |
| *flgD* | b1075 | *flgBCDEFGHIJ* | 7 | 553 | 215 | 160 | 124 | 903 | 489 | 442 | 268 | 81 | 73 | 84 | 553 | flagellar biosynthesis, initiation of hook assembly |
| *flgE* | b1076 | *flgBCDEFGHIJ* | 7 | 706 | 519 | 394 | 558 | 892 | 683 | 664 | 541 | 565 | 504 | 773 | 706 | flagellar biosynthesis, hook protein |
| *flgF* | b1077 | *flgBCDEFGHIJ* | 7 | 238 | 95 | 89 | 54 | 421 | 294 | 206 | 141 | 56 | 66 | 65 | 238 | flagellar biosynthesis, cell-proximal portion of basal-body rod |
| *ndh* | b1109 | *ndh* | 7 | 268 | 287 | 210 | 239 | 351 | 487 | 344 | 224 | 288 | 296 | 364 | 268 | respiratory NADH dehydrogenase |
| *ompW* | b1256 | *ompW* | 7 | 812 | 981 | 221 | 274 | 3225 | 3681 | 3851 | 964 | 572 | 239 | 172 | 812 | putative outer membrane protein |
| *fdnG* | b1474 | *fdnGHI* | 7 | 329 | 494 | 56 | 110 | 99 | 384 | 600 | 208 | 46 | 67 | 58 | 329 | formate dehydrogenase-N, nitrate-inducible, alpha subunit |
| *fdnI* | b1476 | *fdnGHI* | 7 | 297 | 371 | 135 | 170 | 143 | 334 | 534 | 256 | 130 | 130 | 201 | 297 | formate dehydrogenase-N, nitrate-inducible, cytochrome B556(Fdn) gamma subunit |
| *ydfZ* | b1541 | *ydfZ* | 7 | 160 | 121 | 33 | 56 | 126 | 668 | 603 | 123 | 61 | 90 | 67 | 160 | hypothetical protein |
| *ynfE* | b1587 | *ynfEFGH-dmsD* | 7 | 56 | 113 | 19 | 15 | 226 | 587 | 478 | 19 | 23 | 25 | 22 | 56 | putative oxidoreductase, major subunit |
| *ynfF* | b1588 | *ynfEFGH-dmsD* | 7 | 39 | 79 | 12 | 12 | 102 | 530 | 579 | 19 | 25 | 29 | 25 | 39 | putative oxidoreductase, major subunit |
| *ynfG* | b1589 | *ynfEFGH-dmsD* | 7 | 52 | 88 | 26 | 23 | 122 | 555 | 746 | 19 | 30 | 29 | 29 | 52 | putative oxidoreductase, Fe-S subunit |
| *ynfH* | b1590 | *ynfEFGH-dmsD* | 7 | 96 | 113 | 76 | 65 | 122 | 441 | 522 | 90 | 93 | 123 | 117 | 96 | putative DMSO reductase anchor subunit |
| *sodB* | b1656 | *sodB* | 7 | 1627 | 1547 | 1340 | 1314 | 3612 | 2556 | 2959 | 1491 | 699 | 475 | 212 | 1627 | superoxide dismutase, iron |
| *ydhY* | b1674 | *ydhYVW* | 7 | 52 | 73 | 14 | 26 | 333 | 753 | 268 | 62 | 22 | 24 | 23 | 52 | putative oxidoreductase, Fe-S subunit |
| *yeaU* | b1800 | *yeaU* | 7 | 28 | 27 | 31 | 55 | 396 | 215 | 53 | 18 | 79 | 95 | 54 | 28 | putative tartrate dehydrogenase |
| *manX* | b1817 | *manXYZ* | 7 | 2884 | 2391 | 1801 | 1144 | 4887 | 3245 | 3447 | 1123 | 568 | 401 | 372 | 2884 | PTS enzyme IIAB, mannose-specific |
| *manY* | b1818 | *manXYZ* | 7 | 2287 | 2008 | 1531 | 765 | 3114 | 2788 | 2572 | 582 | 400 | 250 | 145 | 2287 | PTS enzyme IIC, mannose-specific |
| *manZ* | b1819 | *manXYZ* | 7 | 1085 | 863 | 598 | 285 | 845 | 1144 | 777 | 138 | 108 | 86 | 47 | 1085 | PTS enzyme IID, mannose-specific |
| *ftnA* | b1905 | *ftnA* | 7 | 725 | 701 | 547 | 479 | 1810 | 2879 | 2802 | 898 | 63 | 85 | 55 | 725 | cytoplasmic ferritin (an iron storage protein) |
| *hisG* | b2019 | *hisLGDCBHAFI* | 7 | 447 | 576 | 462 | 332 | 1058 | 1298 | 1170 | 458 | 319 | 223 | 229 | 447 | ATP phosphoribosyltransferase |
| *hisH* | b2023 | *hisLGDCBHAFI* | 7 | 184 | 217 | 270 | 178 | 314 | 476 | 443 | 247 | 216 | 158 | 162 | 184 | glutamine amidotransferase subunit of heterodimer with HisF = imidazole glycerol phosphate synthase holoenzyme |
| *yeiT* | b2146 | *yeiTA* | 7 | 347 | 681 | 241 | 213 | 1235 | 1658 | 902 | 182 | 72 | 77 | 103 | 347 | putative oxidoreductase |
| *yeiA* | b2147 | *yeiTA* | 7 | 397 | 619 | 384 | 387 | 764 | 1005 | 723 | 411 | 459 | 482 | 678 | 397 | putative oxidoreductase |
| *fruB* | b2169 | *fruBKA* | 7 | 284 | 343 | 176 | 167 | 772 | 456 | 691 | 368 | 135 | 96 | 111 | 284 | PTS system, fructose-specific IIA/fpr component |
| *glpA* | b2241 | *glpABC* | 7 | 2175 | 2052 | 1109 | 633 | 3483 | 2704 | 2940 | 976 | 1470 | 601 | 559 | 2175 | sn-glycerol-3-phosphate dehydrogenase (anaerobic), large subunit |
| *glpB* | b2242 | *glpABC* | 7 | 2368 | 2197 | 1369 | 1274 | 3587 | 2684 | 3386 | 974 | 2328 | 1346 | 1604 | 2368 | sn-glycerol-3-phosphate dehydrogenase (anaerobic), membrane anchor subunit |
| *glpC* | b2243 | *glpABC* | 7 | 992 | 1266 | 768 | 292 | 1399 | 1704 | 1798 | 291 | 810 | 304 | 186 | 992 | sn-glycerol-3-phosphate dehydrogenase (anaerobic), K-small subunit |
| *uraA* | b2497 | *upp-uraA* | 7 | 71 | 51 | 36 | 36 | 309 | 44 | 178 | 307 | 24 | 26 | 29 | 71 | uracil transport |
| *upp* | b2498 | *upp-uraA* | 7 | 633 | 498 | 314 | 327 | 1209 | 636 | 987 | 836 | 154 | 151 | 116 | 633 | uracil phosphoribosyltransferase |
| *guaB* | b2508 | *guaBA* | 7 | 509 | 187 | 193 | 498 | 481 | 562 | 1164 | 309 | 213 | 537 | 248 | 509 | IMP dehydrogenase |
| *yfiD* | b2579 | *yfiD* | 7 | 1513 | 1460 | 101 | 584 | 2306 | 5731 | 3812 | 1032 | 313 | 143 | 113 | 1513 | putative formate acetyltransferase |
| *ygdH* | b2795 | *ygdH* | 7 | 1037 | 1197 | 802 | 578 | 1378 | 1482 | 1190 | 467 | 323 | 146 | 153 | 1037 | hypothetical protein |
| *yqeC* | b2876 | *yqeC* | 7 | 595 | 722 | 194 | 248 | 907 | 758 | 581 | 273 | 185 | 126 | 178 | 595 | hypothetical protein |
| *gcvH* | b2904 | *gcvTHP* | 7 | 2413 | 2054 | 2397 | 1648 | 2771 | 2925 | 2947 | 1354 | 1451 | 529 | 225 | 2413 | in glycine cleavage complex, carrier of aminomethyl moiety via covalently bound lipoyl cofactor |
| *ansB* | b2957 | *ansB* | 7 | 1909 | 2001 | 55 | 427 | 2229 | 2433 | 4741 | 558 | 72 | 64 | 66 | 1909 | periplasmic L-asparaginase II |
| *glcB* | b2976 | *glcDEFGBA* | 7 | 58 | 57 | 64 | 67 | 1709 | 207 | 248 | 84 | 190 | 172 | 118 | 58 | malate synthase G |
| *hybC* | b2994 | *hybOABCDEFG* | 7 | 210 | 439 | 144 | 107 | 203 | 604 | 647 | 78 | 26 | 31 | 23 | 210 | probable large subunit, hydrogenase-2 |
| *hybB* | b2995 | *hybOABCDEFG* | 7 | 332 | 633 | 157 | 132 | 269 | 778 | 775 | 102 | 35 | 53 | 50 | 332 | probable cytochrome Ni/Fe component of hydrogenase-2 |
| *hybA* | b2996 | *hybOABCDEFG* | 7 | 1079 | 1559 | 316 | 416 | 1059 | 1729 | 1788 | 325 | 146 | 157 | 194 | 1079 | hydrogenase-2 small subunit |
| *hybO* | b2997 | *hybOABCDEFG* | 7 | 1039 | 1502 | 177 | 296 | 1040 | 1786 | 1567 | 269 | 54 | 54 | 67 | 1039 | putative hydrogenase subunit |
| *yghZ* | b3001 | *yghZ* | 7 | 433 | 567 | 179 | 159 | 886 | 995 | 680 | 166 | 269 | 196 | 152 | 433 | putative reductase |
| *ygjR* | b3087 | *ygjR* | 7 | 655 | 615 | 256 | 273 | 1036 | 614 | 575 | 513 | 261 | 225 | 196 | 655 | hypothetical protein |
| *uxaA* | b3091 | *uxaCA* | 7 | 78 | 62 | 120 | 62 | 144 | 122 | 200 | 60 | 76 | 74 | 69 | 78 | altronate hydrolase |
| *uxaC* | b3092 | *uxaCA* | 7 | 417 | 435 | 351 | 441 | 659 | 591 | 972 | 496 | 469 | 459 | 893 | 417 | uronate isomerase |
| *tdcF* | b3113 | *tdcABCDEFG* | 7 | 608 | 701 | 600 | 231 | 126 | 2255 | 2296 | 58 | 55 | 65 | 69 | 608 | hypothetical protein |
| *tdcE* | b3114 | *tdcABCDEFG* | 7 | 858 | 1024 | 782 | 354 | 227 | 2489 | 2830 | 85 | 67 | 75 | 95 | 858 | probable formate acetyltransferase 3 |
| *tdcD* | b3115 | *tdcABCDEFG* | 7 | 900 | 1605 | 648 | 322 | 755 | 2571 | 2699 | 62 | 28 | 33 | 46 | 900 | putative kinase |
| *tdcC* | b3116 | *tdcABCDEFG* | 7 | 1206 | 1905 | 597 | 373 | 1525 | 2990 | 3085 | 114 | 91 | 86 | 123 | 1206 | anaerobically inducible L-threonine, L-serine permease |
| *tdcB* | b3117 | *tdcABCDEFG* | 7 | 2149 | 2031 | 401 | 380 | 4099 | 3670 | 3829 | 190 | 28 | 19 | 29 | 2149 | threonine dehydratase, catabolic |
| *tdcA* | b3118 | *tdcABCDEFG* | 7 | 2237 | 2019 | 174 | 700 | 3737 | 2643 | 2886 | 1031 | 137 | 152 | 192 | 2237 | transcriptional activator of tdc operon |
| *pck* | b3403 | *pck* | 7 | 1810 | 1997 | 1935 | 1605 | 3480 | 2283 | 2146 | 1150 | 964 | 646 | 526 | 1810 | phosphoenolpyruvate carboxykinase |
| *malP* | b3417 | *malPQ* | 7 | 954 | 1282 | 616 | 900 | 1185 | 1913 | 1862 | 318 | 179 | 181 | 166 | 954 | maltodextrin phosphorylase |
| *tnaC* | b3707 | *tnaCAB* | 7 | 5082 | 4399 | 9357 | 4872 | 12552 | 9259 | 10705 | 8546 | 2349 | 1149 | 635 | 5082 | tryptophanase leader peptide |
| *tnaA* | b3708 | *tnaCAB* | 7 | 2820 | 2490 | 8679 | 3313 | 11994 | 13322 | 10860 | 4019 | 1790 | 380 | 228 | 2820 | tryptophanase |
| *tnaB* | b3709 | *tnaCAB* | 7 | 429 | 730 | 1432 | 286 | 3708 | 2029 | 2181 | 746 | 305 | 61 | 63 | 429 | low affinity tryptophan permease |
| *asnA* | b3744 | *asnA* | 7 | 154 | 133 | 123 | 129 | 675 | 2597 | 171 | 238 | 126 | 123 | 154 | 154 | asparagine synthetase A |
| *rbsK* | b3752 | *rbsDACBKR* | 7 | 539 | 481 | 846 | 261 | 700 | 530 | 450 | 255 | 117 | 95 | 79 | 539 | ribokinase |
| *glnA* | b3870 | *glnALG* | 7 | 624 | 548 | 828 | 441 | 846 | 777 | 980 | 365 | 362 | 211 | 230 | 624 | glutamine synthetase |
| *katG* | b3942 | *katG* | 7 | 710 | 986 | 758 | 878 | 954 | 1371 | 1735 | 619 | 957 | 665 | 944 | 710 | catalase hydroperoxidase HPI(I) |
| *pepE* | b4021 | *pepE* | 7 | 350 | 444 | 325 | 265 | 321 | 563 | 819 | 197 | 77 | 58 | 58 | 350 | peptidase E, a dipeptidase where amino-terminal residue is aspartate |
| *malF* | b4033 | *malEFG* | 7 | 241 | 168 | 531 | 430 | 900 | 966 | 812 | 273 | 203 | 139 | 158 | 241 | part of maltose permease, periplasmic |
| *malE* | b4034 | *malEFG* | 7 | 1010 | 428 | 1465 | 2285 | 5496 | 3182 | 4780 | 1377 | 602 | 183 | 255 | 1010 | periplasmic maltose-binding protein substrate recognition for transport and chemotaxis |
| *malK* | b4035 | *malK-lamB-malM* | 7 | 626 | 163 | 940 | 1546 | 2805 | 2419 | 2836 | 706 | 75 | 48 | 54 | 626 | ATP-binding component of transport system for maltose |
| *lamB* | b4036 | *malK-lamB-malM* | 7 | 938 | 315 | 1523 | 2904 | 4663 | 3152 | 4407 | 1370 | 250 | 130 | 136 | 938 | phage lambda receptor protein maltose high-affinity receptor |
| *aphA* | b4055 | *aphA* | 7 | 738 | 886 | 672 | 284 | 736 | 1007 | 748 | 219 | 210 | 128 | 148 | 738 | diadenosine tetraphosphatase |
| *adiY* | b4116 | *adiY* | 7 | 392 | 117 | 23 | 85 | 409 | 965 | 280 | 35 | 19 | 22 | 30 | 392 | putative ARAC-type regulatory protein |
| *fumB* | b4122 | *dcuB-fumB* | 7 | 85 | 133 | 32 | 26 | 55 | 1101 | 531 | 16 | 13 | 22 | 19 | 85 | fumarase B= fumarate hydratase Class I anaerobic isozyme |
| *dcuB* | b4123 | *dcuB-fumB* | 7 | 192 | 230 | 45 | 63 | 120 | 1420 | 636 | 69 | 44 | 49 | 41 | 192 | anaerobic dicarboxylate transport |
| *lysU* | b4129 | *lysU* | 7 | 938 | 1148 | 627 | 332 | 1076 | 2235 | 1295 | 218 | 760 | 611 | 364 | 938 | lysine tRNA synthetase, inducible heat shock protein |
| *cadA* | b4131 | *cadBA* | 7 | 63 | 99 | 36 | 42 | 10 | 946 | 323 | 7 | 8 | 8 | 11 | 63 | lysine decarboxylase 1 |
| *cadB* | b4132 | *cadBA* | 7 | 96 | 106 | 55 | 87 | 42 | 720 | 498 | 56 | 38 | 39 | 65 | 96 | transport of lysine/cadaverine |
| *dcuA* | b4138 | *aspA-dcuA* | 7 | 1218 | 1437 | 500 | 549 | 1650 | 2006 | 2002 | 519 | 298 | 229 | 245 | 1218 | anaerobic dicarboxylate transport |
| *frdD* | b4151 | *frdABCD* | 7 | 1008 | 1277 | 660 | 376 | 793 | 1687 | 1750 | 164 | 183 | 178 | 151 | 1008 | fumarate reductase, anaerobic, membrane anchor polypeptide |
| *frdC* | b4152 | *frdABCD* | 7 | 972 | 1262 | 571 | 354 | 810 | 1609 | 1579 | 142 | 137 | 129 | 126 | 972 | fumarate reductase, anaerobic, membrane anchor polypeptide |
| *frdB* | b4153 | *frdABCD* | 7 | 1732 | 1928 | 1039 | 659 | 1725 | 2521 | 2823 | 264 | 346 | 271 | 247 | 1732 | fumarate reductase, anaerobic, iron-sulfur protein subunit |
| *frdA* | b4154 | *frdABCD* | 7 | 2400 | 2223 | 1204 | 1081 | 2791 | 3739 | 3645 | 607 | 556 | 508 | 471 | 2400 | fumarate reductase, anaerobic, flavoprotein subunit |
| *treC* | b4239 | *treBC* | 7 | 1291 | 1285 | 2076 | 1273 | 2655 | 1838 | 2438 | 826 | 27 | 9 | 84 | 1291 | trehalase 6-P hydrolase |
| *treB* | b4240 | *treBC* | 7 | 3093 | 3303 | 4871 | 2608 | 5503 | 3290 | 5164 | 2454 | 158 | 134 | 114 | 3093 | PTS system enzyme II, trehalose specific |
| *pyrI* | b4244 | *pyrLBI* | 7 | 209 | 98 | 157 | 104 | 4492 | 374 | 3436 | 445 | 121 | 109 | 101 | 209 | aspartate carbamoyltransferase, regulatory subunit |
| *pyrB* | b4245 | *pyrLBI* | 7 | 221 | 74 | 135 | 58 | 5878 | 568 | 4324 | 540 | 79 | 79 | 67 | 221 | aspartate carbamoyltransferase, catalytic subunit |
| *tdcG* | b4471 | *tdcABCDEFG* | 7 | 186 | 187 | 218 | 73 | 48 | 763 | 843 | 41 | 37 | 42 | 60 | 186 | L-serine deaminase 3 |
