# Supplementary material for: The HU Regulon Is Composed of Genes Responding to Anaerobiosis, Acid Stress, High Osmolarity and SOS Induction
Source: PLoS One. 2009 Feb 4;4(2):e4367. doi: 10.1371/journal.pone.0004367 (PMC2634741; doi:10.1371/journal.pone.0004367)
Supplement: Table S4 — Operons composing the HU regulon (0.32 MB DOC) [file pone.0004367.s006.doc]

**Supplemental Table S4**. Operons composing the HU regulon

| **Operon** | **Cluster** | **Coordinate** | **Orientation** |
| --- | --- | --- | --- |
| *nhaAR* | 2 | 17489 | forward |
| *ybaY* | 2 | 474603 | forward |
| *ybaST* | 2 | 510865 | forward |
| *entCEBA-ybdB* | 2 | 624108 | forward |
| *ybgS* | 2 | 784540 | reverse |
| *ybhB* | 2 | 807132 | reverse |
| *ybiI* | 2 | 837679 | reverse |
| *ybiM* | 2 | 841279 | reverse |
| *dps* | 2 | 848134 | reverse |
| *ybjP* | 2 | 903690 | reverse |
| *poxB-ltaE-ybjT* | 2 | 910272 | reverse |
| *ycaC* | 2 | 944780 | reverse |
| *hspQ* | 2 | 1027944 | reverse |
| *hyaABCDEF* | 2 | 1031362 | forward |
| *cbpAM* | 2 | 1062998 | reverse |
| *wrbA-yccJ* | 2 | 1066931 | reverse |
| *msyB* | 2 | 1113404 | reverse |
| *grxB* | 2 | 1123277 | reverse |
| *ycgZ* | 2 | 1215012 | forward |
| *ymgAB* | 2 | 1215291 | forward |
| *ymgC* | 2 | 1215971 | forward |
| *yciGFE* | 2 | 1314059 | reverse |
| *trpLEDCBA* | 2 | 1321106 | reverse |
| *osmB* | 2 | 1341352 | reverse |
| *yciW* | 2 | 1348131 | reverse |
| *yncG* | 2 | 1524271 | forward |
| *adhP* | 2 | 1551862 | reverse |
| *bdm-sra* | 2 | 1554304 | reverse |
| *yddV-dos* | 2 | 1564825 | reverse |
| *gadBC* | 2 | 1570069 | reverse |
| *ydeI* | 2 | 1622521 | reverse |
| *sodC* | 2 | 1722679 | reverse |
| *ydhL* | 2 | 1723944 | reverse |
| *cfa* | 2 | 1739437 | forward |
| *sufABCDSE* | 2 | 1762410 | reverse |
| *ydiJIH* | 2 | 1766709 | reverse |
| *katE* | 2 | 1811891 | forward |
| *osmE* | 2 | 1820280 | reverse |
| *spy* | 2 | 1823649 | reverse |
| *yebV* | 2 | 1919804 | forward |
| *otsBA* | 2 | 1980411 | reverse |
| *yodC* | 2 | 2026394 | reverse |
| *hchA* | 2 | 2033859 | forward |
| *fbaB* | 2 | 2176586 | reverse |
| *yehE* | 2 | 2190818 | reverse |
| *mlrA* | 2 | 2212888 | forward |
| *elaB* | 2 | 2379049 | reverse |
| *yfcG* | 2 | 2418643 | forward |
| *talA* | 2 | 2576688 | forward |
| *tktB* | 2 | 2577658 | forward |
| *ygaU* | 2 | 2794808 | reverse |
| *ygaM* | 2 | 2798156 | forward |
| *ygiW* | 2 | 3167698 | reverse |
| *yqjCDEK* | 2 | 3246991 | forward |
| *yhbO* | 2 | 3296996 | forward |
| *yrbL* | 2 | 3346474 | forward |
| *yhcO* | 2 | 3384151 | reverse |
| *yhfG-fic-pabA* | 2 | 3489642 | reverse |
| *yhhA* | 2 | 3584966 | forward |
| *yhiM* | 2 | 3632864 | forward |
| *slp-dctR* | 2 | 3651984 | forward |
| *yhiD* | 2 | 3653925 | reverse |
| *hdeAB* | 2 | 3654763 | reverse |
| *hdeD* | 2 | 3655018 | forward |
| *gadE-mdtEF* | 2 | 3656389 | forward |
| *gadW* | 2 | 3662641 | reverse |
| *gadAX* | 2 | 3665603 | reverse |
| *yhjY* | 2 | 3710957 | reverse |
| *yiaG* | 2 | 3717501 | forward |
| *cpxP* | 2 | 4103843 | forward |
| *aceBAK* | 2 | 4213501 | forward |
| *yjbJ* | 2 | 4257260 | forward |
| *yjdN* | 2 | 4323764 | reverse |
| *yjdIJ* | 2 | 4349866 | forward |
| *aidB* | 2 | 4412298 | forward |
| *ytfK* | 2 | 4437610 | forward |
| *yjiD* | 2 | 4555016 | forward |
| *osmY* | 2 | 4609419 | forward |
| *tsx* | 4 | 431237 | reverse |
| *dcuC* | 4 | 655191 | reverse |
| *nagBACD* | 4 | 702834 | reverse |
| *nagE* | 4 | 703167 | forward |
| *galETKM* | 4 | 791278 | reverse |
| *dmsABC* | 4 | 940182 | forward |
| *dhaKLM* | 4 | 1250061 | reverse |
| *narK* | 4 | 1277180 | forward |
| *narGHJI* | 4 | 1279087 | forward |
| *adhE* | 4 | 1297344 | reverse |
| *ydeN* | 4 | 1580548 | reverse |
| *napFDAGHBC-ccmABCDEFGH* | 4 | 2301519 | reverse |
| *dsdXA* | 4 | 2475869 | forward |
| *yfeYX* | 4 | 2549238 | reverse |
| *srlAEBD-gutM-srlR-gutQ* | 4 | 2823854 | forward |
| *hypABCDE-fhlA* | 4 | 2848669 | forward |
| *galP* | 4 | 3086306 | forward |
| *garPLRK-rnpB* | 4 | 3272929 | reverse |
| *garD* | 4 | 3273304 | forward |
| *deaD* | 4 | 3305882 | reverse |
| *nirBDC-cysG* | 4 | 3492033 | forward |
| *feoAB* | 4 | 3538185 | forward |
| *yhjX* | 4 | 3710030 | reverse |
| *cspA* | 4 | 3718072 | forward |
| *ysaA* | 4 | 3739605 | reverse |
| *gpmM-envC-yibQ* | 4 | 3783283 | forward |
| *udp* | 4 | 4014454 | forward |
| *spf* | 4 | 4047922 | forward |
| *pfkA* | 4 | 4105575 | forward |
| *nrfABCDEFG* | 4 | 4285787 | forward |
| *yjdKO* | 4 | 4350607 | forward |
| *purA* | 4 | 4402710 | forward |
| *yjiXA* | 4 | 4587102 | reverse |
| *yjiY* | 4 | 4589302 | reverse |
| *yjjW* | 4 | 4613566 | reverse |
| *yjjI* | 4 | 4615088 | reverse |
| *deoCABD* | 4 | 4615346 | forward |
| *dnaK-tpke11-dnaJ* | 5 | 12163 | forward |
| *can* | 5 | 142670 | reverse |
| *cyoABCDE* | 5 | 450834 | reverse |
| *metT-leuW-glnUW-metU-glnVX* | 5 | 696356 | reverse |
| *gltA* | 5 | 753691 | reverse |
| *sdhCDAB-b0725-sucABCD* | 5 | 754400 | forward |
| *putP* | 5 | 1078528 | forward |
| *rnb* | 5 | 1346936 | reverse |
| *paaABCDEFGHIJK* | 5 | 1451951 | forward |
| *ydcI* | 5 | 1493095 | reverse |
| *fumA* | 5 | 1686401 | reverse |
| *pps* | 5 | 1785136 | reverse |
| *fliAZY* | 5 | 1999813 | reverse |
| *yedEF* | 5 | 2006301 | forward |
| *yeeED* | 5 | 2083549 | reverse |
| *mglBAC* | 5 | 2238370 | reverse |
| *cysK* | 5 | 2530431 | forward |
| *cysPUWAM* | 5 | 2541550 | reverse |
| *maeB* | 5 | 2576399 | reverse |
| *ndk* | 5 | 2642886 | reverse |
| *iscRSUA* | 5 | 2660153 | reverse |
| *clpB* | 5 | 2732195 | reverse |
| *proVWX* | 5 | 2802837 | forward |
| *cysDNC* | 5 | 2874351 | reverse |
| *cysJIH* | 5 | 2889920 | reverse |
| *sdaCB* | 5 | 2926251 | forward |
| *yqgB* | 5 | 3084088 | reverse |
| *nanATEK-yhcH* | 5 | 3371598 | reverse |
| *dctA* | 5 | 3681470 | reverse |
| *lldPRD* | 5 | 3775422 | forward |
| *uhpT* | 5 | 3845190 | reverse |
| *ibpAB* | 5 | 3865445 | reverse |
| *atpIBEFHAGDC* | 5 | 3920463 | reverse |
| *sbp* | 5 | 4106857 | forward |
| *hslVU* | 5 | 4120310 | reverse |
| *sthA* | 5 | 4158813 | reverse |
| *thrU-tyrU-glyT-thrT-tufB* | 5 | 4173411 | forward |
| *groSL* | 5 | 4368711 | forward |
| *nanC-yjhT-yjhS* | 5 | 4537524 | reverse |
| *fimAICDFGH* | 5 | 4541138 | forward |
| *ykgMO* | 6 | 312001 | reverse |
| *sulA* | 6 | 1020142 | reverse |
| *cspG* | 6 | 1050684 | forward |
| *dinI* | 6 | 1120710 | reverse |
| *ymfH-xisE-intE* | 6 | 1200603 | reverse |
| *ymfJ* | 6 | 1201307 | reverse |
| *ymfTLMNROPQ-ycfK-ymfS* | 6 | 1202247 | forward |
| *umuDC* | 6 | 1229990 | forward |
| *yebG* | 6 | 1928771 | reverse |
| *nrdHIEF* | 6 | 2798745 | forward |
| *recAX* | 6 | 2821791 | reverse |
| *mqsR-ygiT* | 6 | 3166566 | reverse |
| *bglGFB* | 6 | 3904590 | reverse |
| *sodA* | 6 | 4098833 | forward |
| *ptsA-fsaB-gldA* | 6 | 4140244 | reverse |
| *fecABCDE* | 6 | 4514700 | reverse |
| *rihC* | 7 | 27293 | forward |
| *carAB* | 7 | 29651 | forward |
| *pdhR-aceEF-lpd* | 7 | 122092 | forward |
| *ykgEFG* | 7 | 320832 | forward |
| *ybcW* | 7 | 579103 | forward |
| *rihA* | 7 | 683635 | reverse |
| *asnB* | 7 | 698400 | reverse |
| *cydAB* | 7 | 770681 | forward |
| *ybgE* | 7 | 773532 | forward |
| *focA-pflB* | 7 | 953689 | reverse |
| *ycbJ* | 7 | 970975 | forward |
| *flgBCDEFGHIJ* | 7 | 1130241 | forward |
| *ndh* | 7 | 1165308 | forward |
| *ompW* | 7 | 1312044 | forward |
| *fdnGHI* | 7 | 1545425 | forward |
| *ydfZ* | 7 | 1627239 | forward |
| *ynfEFGH-dmsD* | 7 | 1656093 | forward |
| *sodB* | 7 | 1733402 | forward |
| *ydhYVW* | 7 | 1752501 | reverse |
| *yeaU* | 7 | 1879936 | forward |
| *manXYZ* | 7 | 1900072 | forward |
| *ftnA* | 7 | 1986740 | forward |
| *hisLGDCBHAFI* | 7 | 2088020 | forward |
| *yeiTA* | 7 | 2232055 | forward |
| *fruBKA* | 7 | 2261517 | reverse |
| *glpABC* | 7 | 2350669 | forward |
| *upp-uraA* | 7 | 2618894 | reverse |
| *guaBA* | 7 | 2632092 | reverse |
| *yfiD* | 7 | 2714471 | reverse |
| *ygdH* | 7 | 2924330 | forward |
| *yqeC* | 7 | 3013079 | reverse |
| *gcvTHP* | 7 | 3048689 | reverse |
| *ansB* | 7 | 3098750 | reverse |
| *glcDEFGBA* | 7 | 3126043 | reverse |
| *hybOABCDEFG* | 7 | 3144283 | reverse |
| *yghZ* | 7 | 3145919 | forward |
| *ygjR* | 7 | 3235315 | forward |
| *uxaCA* | 7 | 3242763 | reverse |
| *tdcABCDEFG* | 7 | 3265087 | reverse |
| *pck* | 7 | 3530840 | forward |
| *malPQ* | 7 | 3550495 | reverse |
| *tnaCAB* | 7 | 3886458 | forward |
| *asnA* | 7 | 3925178 | forward |
| *rbsDACBKR* | 7 | 3931374 | forward |
| *glnALG* | 7 | 4056057 | reverse |
| *katG* | 7 | 4131858 | forward |
| *pepE* | 7 | 4228165 | reverse |
| *malEFG* | 7 | 4244442 | reverse |
| *malK-lamB-malM* | 7 | 4244807 | forward |
| *aphA* | 7 | 4267437 | forward |
| *adiY* | 7 | 4335952 | reverse |
| *dcuB-fumB* | 7 | 4346767 | reverse |
| *lysU* | 7 | 4352740 | reverse |
| *cadBA* | 7 | 4358054 | reverse |
| *aspA-dcuA* | 7 | 4366350 | reverse |
| *frdABCD* | 7 | 4380341 | reverse |
| *treBC* | 7 | 4464203 | reverse |
| *pyrLBI* | 7 | 4470556 | reverse |
