# Supplementary material for: The HU Regulon Is Composed of Genes Responding to Anaerobiosis, Acid Stress, High Osmolarity and SOS Induction
Source: PLoS One. 2009 Feb 4;4(2):e4367. doi: 10.1371/journal.pone.0004367 (PMC2634741; doi:10.1371/journal.pone.0004367)
Supplement: Table S5 — Chaperone and stress functions in the HU regulon. (0.27 MB DOC) [file pone.0004367.s007.doc]

**Supplemental Table S5.** Chaperone and stress functions in the HU regulon.

| **Gene** | **Blattner** | **Stress** | **Function** | **Cluster** |
| --- | --- | --- | --- | --- |
| *dnaK* | b0014 | chaperone | chaperone Hsp70, DNA biosynthesis, autoregulated heat shock proteins | 5 |
| *dnaJ* | b0015 | chaperone | chaperone with DnaK, heat shock protein | 5 |
| *cbpA* | b1000 | chaperone | curved DNA-binding protein, DnaJ homologue that functions as a co-chaperone of DnaK | 2 |
| *narJ* | b1226 | chaperone | molybdenum-cofactor-assembly chaperone subunit (delta subunit) of nitrate reductase 1 | 4 |
| *narJ* | b1226 | chaperone | Chaperone for NarGH, molybdenum insertion | 4 |
| *hchA* | b1967 | chaperone | Hsp31 molecular chaperone | 2 |
| *clpB* | b2592 | chaperone | protein disaggregation chaperone | 5 |
| *hypC* | b2728 | chaperone | Hydrogenase 3 chaperone-type protein | 4 |
| *yhbO* | b3153 | chaperone | Confers resistance to various stresses, chaperone homolog | 2 |
| *hdeA* | b3510 | chaperone | Periplasmic chaperone of acid-denatured proteins | 2 |
| *cspA* | b3556 | chaperone | Cold-inducible RNA chaperone and anti-terminator | 4 |
| *ibpB* | b3686 | chaperone | heat shock chaperone | 5 |
| *ibpA* | b3687 | chaperone | heat shock chaperone | 5 |
| *hslU* | b3931 | chaperone | heat shock protein hslVU, ATPase subunit, homologous to chaperones | 5 |
| *groS* | b4142 | chaperone | (mopB)Cpn10 chaperonin GroES, small subunit of GroESL | 5 |
| *groL* | b4143 | chaperone | (mobpA)Cpn60 chaperonin GroEL, large subunit of GroESL | 5 |
| *fimC* | b4316 | chaperone | periplasmic chaperone, required for type 1 fimbriae | 5 |
| *cspG* | b0990 | cold shock | homolog of Salmonella cold shock protein | 6 |
| *ycgZ* | b1164 | cold shock | Cold shock gene function unknown | 2 |
| *cspA* | b3556 | cold shock | cold shock protein 7.4 transcriptional activator of hns | 4 |
| *dps* | b0812 | H_2_O_2_ induced | Stress response DNA binding protein | 2 |
| *ycgZ* | b1164 | H_2_O_2_ induced | Function unknown | 2 |
| *ymgB* | b1166 | H_2_O_2_ induced | Function unknown | 2 |
| *yeeD* | b2012 | H_2_O_2_ induced | Function unknown | 5 |
| *dsdX* | b2365 | H_2_O_2_ induced | Homology with gluconate permease | 4 |
| *dsdA* | b2366 | H_2_O_2_ induced | D-Serine deaminase | 4 |
| *cysK* | b2414 | H_2_O_2_ induced | Cysteine synthase | 5 |
| *ibpB* | b3686 | H_2_O_2_ induced | Chaperone heat-inducible protein of HSP20 family | 5 |
| *ibpA* | b3687 | H_2_O_2_ induced | Chaperone heat-inducible protein of HSP20 family | 5 |
| *tnaA* | b3708 | H_2_O_2_ induced | Tryptophanase | 7 |
| *sbp* | b3917 | H_2_O_2_ induced | Periplasmic sulfate binding protein | 5 |
| *katG* | b3942 | H_2_O_2_ induced | Catalase hydrogen peroxidase I | 7 |
| *yjiD* | b4326 | H_2_O_2_ induced | Function unknown | 2 |
| *dnaK* | b0014 | heat shock | chaperone Hsp70, DNA biosynthesis, autoregulated heat shock proteins | 5 |
| *dnaJ* | b0015 | heat shock | chaperone with DnaK, heat shock protein | 5 |
| *hspQ* | b0966 | heat shock | heat shock protein, DNA-binding, hemimethylated | 2 |
| *hchA* | b1967 | heat shock | (yedU,yzzC) Aminopeptidase and molecular chaperone Hsp31, heat-inducible | 2 |
| *clpB* | b2592 | heat shock | heat shock protein | 5 |
| *ibpB* | b3686 | heat shock | heat shock protein | 5 |
| *ibpA* | b3687 | heat shock | heat shock protein | 5 |
| *hslU* | b3931 | heat shock | heat shock protein hslVU, ATPase subunit, homologous to chaperones | 5 |
| *hslV* | b3932 | heat shock | heat shock protein hslVU, proteasome-related peptidase subunit | 5 |
| *lysU* | b4129 | heat shock | lysine tRNA synthetase, inducible, heat shock protein | 7 |
| *groL* | b4143 | heat shock | GroEL, chaperone Hsp60, peptide-dependent ATPase, heat shock protein | 5 |
| *ymgA* | b1165 | Induced in *ΔoxyR* | Function unknown | 2 |
| *ymgB* | b1166 | Induced in *ΔoxyR* | Function unknown | 2 |
| *ydeN* | b1498 | Induced in *ΔoxyR* | Function unknown | 4 |
| *manX* | b1817 | Induced in *ΔoxyR* | Mannose phosphotransferase system | 7 |
| *yeeD* | b2012 | Induced in *ΔoxyR* | Function unknown | 5 |
| *dsdX* | b2365 | Induced in *ΔoxyR* | Homology with gluconate permease | 4 |
| *dsdA* | b2366 | Induced in *ΔoxyR* | D-Serine deaminase | 4 |
| *cysK* | b2414 | Induced in *ΔoxyR* | Cysteine synthase | 5 |
| *cysP* | b2425 | Induced in *ΔoxyR* | Periplasmic sulfate binding protein | 5 |
| *ibpB* | b3686 | Induced in *ΔoxyR* | Chaperone heat-inducible protein of HSP20 family | 5 |
| *ibpA* | b3687 | Induced in *ΔoxyR* | Chaperone heat-inducible protein of HSP20 family | 5 |
| *tnaC* | b3707 | Induced in *ΔoxyR* | Regulatory leader peptide for tna operon | 7 |
| *tnaA* | b3708 | Induced in *ΔoxyR* | Tryptophanase | 7 |
| *sbp* | b3917 | Induced in *ΔoxyR* | Periplasmic sulfate binding protein | 5 |
| *ytfK* | b4217 | Induced in *ΔoxyR* | Function unknown | 2 |
| *yjiD* | b4326 | Induced in *ΔoxyR* | Function unknown | 2 |
| *treB* | b4240 | NaSal down | IITre, translocation system, Tre-specific PTS enzyme II | 7 |
| *pyrB* | b4245 | NaSal down | Aspartate transcarbamylase, catalytic subunit | 7 |
| *dnaK* | b0014 | NaSal up | HSP-70-type molecular chaperone | 5 |
| *ybaS* | b0485 | NaSal up | Putative glutaminase | 2 |
| *entC* | b0593 | NaSal up | Isochorismate synthetase | 2 |
| *gltA* | b0720 | NaSal up | Citrate synthase | 5 |
| *dps* | b0812 | NaSal up | Stress response DNA-binding protein | 2 |
| *pflB* | b0903 | NaSal up | Pyruvate formate lyase I induced anaerobically | 7 |
| *wrbA* | b1004 | NaSal up | Affects association between Trp repressor and operators in stationary phase | 2 |
| *ycgZ* | b1164 | NaSal up | Function unknown | 2 |
| *ymgA* | b1165 | NaSal up | Function unknown | 2 |
| *dhaM* | b1198 | NaSal up | Function unknown | 4 |
| *dhaK* | b1200 | NaSal up | Putative dihydroxyacetone kinase | 4 |
| *adhE* | b1241 | NaSal up | Acetaldehyde-coenzyme A dehydrogenase | 4 |
| *gadB* | b1493 | NaSal up | Glutamate decarboxylase | 2 |
| *cfa* | b1661 | NaSal up | Cyclopropane fatty acid synthase | 2 |
| *katE* | b1732 | NaSal up | Catalase hydroperoxidase III | 2 |
| *manX* | b1817 | NaSal up | PTS family, mannose-specific enzyme IIA component | 7 |
| *manY* | b1818 | NaSal up | Mannose PTS, EIIC component | 7 |
| *otsB* | b1897 | NaSal up | Trehalose phosphate phosphatase | 2 |
| *elaB* | b2266 | NaSal up | Function unknown | 2 |
| *cysK* | b2414 | NaSal up | Cysteine synthase | 5 |
| *ygaM* | b2672 | NaSal up | Function unknown | 2 |
| *srlE* | b2703 | NaSal up | PTS family, glucitol/sorbitol-specific enzyme IIB component | 4 |
| *ygiW* | b3024 | NaSal up | Function unknown | 2 |
| *yqjD* | b3098 | NaSal up | Function unknown | 2 |
| *slp* | b3506 | NaSal up | C starvation and stationary phase-inducible outer membrane lipoprotein | 2 |
| *hdeB* | b3509 | NaSal up | Periplasmic, unknown function, has sigma S-dependent promoter | 2 |
| *hdeA* | b3510 | NaSal up | Periplasmic, unknown function, has sigma S-dependent promoter | 2 |
| *gadX* | b3516 | NaSal up | Putative transcriptional regulator (AraC/XylS family) | 2 |
| *gadA* | b3517 | NaSal up | Glutamate decarboxylase | 2 |
| *yiaG* | b3555 | NaSal up | Putative transcriptional regulator | 2 |
| *lldP* | b3603 | NaSal up | L-Lactate permease | 5 |
| *lldR* | b3604 | NaSal up | Regulatory gene for lld operon | 5 |
| *sodA* | b3908 | NaSal up | Member of soxRS regulon superoxide dismutase, Mn | 6 |
| *aceB* | b4014 | NaSal up | Malate synthase A | 2 |
| *lysU* | b4129 | NaSal up | Lysyl tRNA synthetase, inducible | 7 |
| *osmY* | b4376 | NaSal up | Periplasmic, sigma S-dependent protein (stationary phase) | 2 |
| *deoB* | b4383 | NaSal up | Deoxyribouratase, phosphopentomutase | 4 |
| *dps* | b0812 | OxyR Regulon | Stress response DNA binding protein | 2 |
| *sufE* | b1679 | OxyR Regulon | Function unknown | 2 |
| *katG* | b3942 | OxyR Regulon | Catalase hydrogen peroxidase I | 7 |
| *narG* | b1224 | Paraquat down | Nitrate reductase alpha subunit | 4 |
| *sodC* | b1646 | Paraquat down | Superoxide dismutase, Cu, Zn | 2 |
| *sodB* | b1656 | Paraquat down | Superoxide dismutase, Fe | 7 |
| *uraA* | b2497 | Paraquat down | Uracil concentration dependence of pyr mutants Ura ABC transporter | 7 |
| *ygjR* | b3087 | Paraquat down | Putative NADP-binding dehydrogenase | 7 |
| *gadX* | b3516 | Paraquat down | Putative transcriptional regulator (AraC/XylS family) | 2 |
| *pyrB* | b4245 | Paraquat down | Aspartate transcarbamylase, catalytic subunit | 7 |
| *yjjI* | b4380 | Paraquat down | Function unknown | 4 |
| *cyoD* | b0429 | Paraquat up | Cytochrome o oxidase subunit IV | 5 |
| *gltA* | b0720 | Paraquat up | Citrate synthase | 5 |
| *sdhB* | b0724 | Paraquat up | Succinate dehydrogenase iron-sulfur protein | 5 |
| *sucD* | b0729 | Paraquat up | Succinyl-coenzyme A synthetase alpha subunit | 5 |
| *dps* | b0812 | Paraquat up | Stress response DNA-binding protein | 2 |
| *cysK* | b2414 | Paraquat up | Cysteine synthase A | 5 |
| *cysD* | b2752 | Paraquat up | Sulfate adenylyltransferase | 2 |
| *tnaA* | b3708 | Paraquat up | Tryptophanase | 7 |
| *sodA* | b3908 | Paraquat up | Superoxide dismutase, Mn | 6 |
| *malE* | b4034 | Paraquat up | Maltose-binding protein, periplasmic transport and chemotaxis | 7 |
| *malK* | b4035 | Paraquat up | Maltose transport complex, ATP-binding subunit | 7 |
| *lamB* | b4036 | Paraquat up | Maltose high-affinity uptake | 7 |
| *cadA* | b4131 | Paraquat up | Lysine decarboxylase | 7 |
| *ytfK* | b4217 | Paraquat up | Function unknown | 2 |
| *deoB* | b4383 | Paraquat up | Deoxyribouratase, phosphopentomutase | 4 |
| *dps* | b0812 | stress | Stress response DNA-binding protein | 2 |
| *osmB* | b1283 | stress | Osmotically and stress inducible lipoprotein | 2 |
| *spy* | b1743 | stress | envelope stress induced periplasmic protein, induced by zinc and envelope stress | 2 |
| *yhbO* | b3153 | stress | Confers resistance to various stresses, chaperone homolog | 2 |
| *hdeA* | b3510 | stress | stress response protein acid-resistance protein | 2 |
| *yjbJ* | b4045 | stress | predicted stress response protein | 2 |
| *cpxP* | b4484 | stress | periplasmic protein combats stress | 2 |
