# Supplementary material for: The HU Regulon Is Composed of Genes Responding to Anaerobiosis, Acid Stress, High Osmolarity and SOS Induction
Source: PLoS One. 2009 Feb 4;4(2):e4367. doi: 10.1371/journal.pone.0004367 (PMC2634741; doi:10.1371/journal.pone.0004367)
Supplement: Table S6 — Comparison of the genes regulated by DNA supercoiling by Blot et al (2006) (1) and Peter et al (2004) (2) (0.08 MB DOC) [file pone.0004367.s008.doc]

**Supplemental Table S6. Comparison of the genes regulated by DNA supercoiling by Blot *et al* (2006) ^(1)^ and Peter *et al* (2004) ^(2)^**

| **Gene** | **Blattner** | **Reg.^1^** | **Reg.^2^** | **Function** |
| --- | --- | --- | --- | --- |
| *nhaA* | b0019 | Rel | Rel | Na+/H antiporter; pH dependent |
| *ribF* | b0025 | Hyp | Hyp | putative regulator |
| *lpcA* | b0222 | Hyp | Hyp | phosphoheptose isomerase |
| *yafK* | b0224 | Hyp | Hyp | orf; hypothetical protein |
| *crl* | b0240 | Hyp | Hyp | transcriptional regulator of cryptic csgA gene for curli surface fibers |
| *ispA* | b0421 | Hyp | Hyp | geranyltranstransferase (farnesyldiphosphate synthase) |
| *smtA* | b0921 | Hyp | Hyp | S-adenosylmethionine-dependent methyltransferase |
| *mukF* | b0922 | Hyp | Hyp | mukF protein (killing factor KICB) |
| *mukE* | b0923 | Hyp | Hyp | orf; hypothetical protein |
| *rimJ* | b1066 | Hyp | Hyp | acetylation of N-terminal alanine of 30S ribosomal subunit protein S5 |
| *minD* | b1175 | Hyp | Hyp | cell division inhibitor; a membrane ATPase; activates minC |
| *minC* | b1176 | Hyp | Hyp | cell division inhibitor; inhibits ftsZ ring formation |
| *ycgL* | b1179 | Hyp | Hyp | orf; hypothetical protein |
| *ydiA* | b1703 | Hyp | Hyp | orf; hypothetical protein |
| *yebR* | b1832 | Hyp | Hyp | orf; hypothetical protein |
| *yobA* | b1841 | Hyp | Hyp | orf; hypothetical protein |
| *holE* | b1842 | Hyp | Hyp | DNA polymerase III; theta subunit |
| *yebK* | b1853 | Hyp | Hyp | orf; hypothetical protein |
| *msbB* | b1855 | Hyp | Hyp | suppressor of htrB; heat shock protein |
| *yeeN* | b1983 | Rel | Rel | orf; hypothetical protein |
| *purC* | b2476 | Hyp | Rel | phosphoribosylaminoimidazole-succinocarboxamide synthetase = SAICAR synthetase |
| *gcvR* | b2479 | Hyp | Hyp | transcriptional regulation of gcv operon |
| *bcp* | b2480 | Hyp | Hyp | bacterioferritin comigratory protein |
| *ppk* | b2501 | Hyp | Hyp | polyphosphate kinase |
| *yfgA* | b2516 | Hyp | Hyp | putative membrane protein |
| *ygaH* | b2683 | Hyp | Hyp | orf; hypothetical protein |
| *xerD* | b2894 | Hyp | Hyp | site-specific recombinase |
| *yggX* | b2962 | Hyp | Hyp | orf; hypothetical protein |
| *yraM* | b3147 | Hyp | Hyp | putative glycosylase |
| *yrbL* | b3207 | Hyp | Hyp | orf; hypothetical protein |
| *yrdD* | b3283 | Hyp | Hyp | putative DNA topoisomerase |
| *smg* | b3284 | Hyp | Hyp | orf; hypothetical protein |
| *htrL* | b3618 | Rel | Rel | involved in lipopolysaccharide biosynthesis |
| *rfaZ* | b3624 | Rel | Rel | lipopolysaccharide core biosynthesis |
| *gyrB* | b3699 | Rel | Rel | DNA gyrase subunit B; type II topoisomerase; ATPase activity |
| *ubiC* | b4039 | Hyp | Hyp | chorismate lyase |
| *holC* | b4259 | Hyp | Hyp | DNA polymerase III; chi subunit |
