# Supplementary material for: The HU Regulon Is Composed of Genes Responding to Anaerobiosis, Acid Stress, High Osmolarity and SOS Induction
Source: PLoS One. 2009 Feb 4;4(2):e4367. doi: 10.1371/journal.pone.0004367 (PMC2634741; doi:10.1371/journal.pone.0004367)
Supplement: Table S7 — Comparison of the genes regulated by HU (1) and by DNA supercoiling by Blot et al (2006) (2). (0.03 MB DOC) [file pone.0004367.s009.doc]

**Supplemental Table S7. Comparison of the genes regulated by HU ^(1)^ and by DNA supercoiling by Blot *et al* (2006) ^(2)^**

| **Gene** | **Blattner** | **Reg.^1^** | **Reg.^2^** | **Function** |
| --- | --- | --- | --- | --- |
| *nhaA* | b0019 | [Cluster2] | Rel | Na+/H antiporter; pH dependent |
| *spy* | b1743 | [Cluster2] | Rel | periplasmic protein related to spheroblast formation |
| *yrbL* | b3207 | [Cluster2] | Hyp | orf; hypothetical protein |
| *clpB* | b2592 | [Cluster5] | Rel | heat shock protein |
| *ibpA* | b3687 | [Cluster5] | Rel | heat shock protein |
| *sulA* | b0958 | [Cluster6] | Hyp | suppressor of lon-- inhibits cell division and ftsZ ring formation |
| *fdnI* | b1476 | [Cluster7] | Rel | formate dehydrogenase-N; nitrate-inducible; cytochrome B556(Fdn) gamma subunit |
| *gcvH* | b2904 | [Cluster7] | Hyp | in glycine cleavage complex; carrier of aminomethyl moiety via covalently bound lipoyl cofactor |
