# Supplementary material for: The HU Regulon Is Composed of Genes Responding to Anaerobiosis, Acid Stress, High Osmolarity and SOS Induction
Source: PLoS One. 2009 Feb 4;4(2):e4367. doi: 10.1371/journal.pone.0004367 (PMC2634741; doi:10.1371/journal.pone.0004367)
Supplement: Table S8 — Comparison of the genes regulated by HU (1) and by DNA supercoiling by Peter et al (2004) (2) (0.04 MB DOC) [file pone.0004367.s010.doc]

**Supplemental Table S8. Comparison of the genes regulated by HU ^(1)^ and by DNA supercoiling by Peter *et al* (2004) ^(2)^**

| **Gene** | **Blattner** | **Reg.^1^** | **Reg.^2^** | **Function** |
| --- | --- | --- | --- | --- |
| *nhaA* | b0019 | [Cluster2] | Rel | Na+/H antiporter; pH dependent |
| *adhP* | b1478 | [Cluster2] | Rel | alcohol dehydrogenase |
| *gadB* | b1493 | [Cluster2] | Rel | glutamate decarboxylase isozyme |
| *otsB* | b1897 | [Cluster2] | Rel | trehalose-6-phosphate phophatase; biosynthetic |
| *yfcG* | b2302 | [Cluster2] | Rel | putative S-transferase |
| *yrbL* | b3207 | [Cluster2] | Hyp | orf; hypothetical protein |
| *dcuC* | b0621 | [Cluster4] | Rel | transport of dicarboxylates |
| *can* | b0126 | [Cluster5] | Hyp | putative carbonic anhdrase (EC 4.2.1.1) |
| *fimI* | b4315 | [Cluster5] | Hyp | fimbrial protein |
| *fimC* | b4316 | [Cluster5] | Hyp | periplasmic chaperone; required for type 1 fimbriae |
| *ynfF* | b1588 | [Cluster7] | Rel | putative oxidoreductase; major subunit |
| *tdcB* | b3117 | [Cluster7] | Rel | threonine dehydratase; catabolic |
