# Supplementary material for: The HU Regulon Is Composed of Genes Responding to Anaerobiosis, Acid Stress, High Osmolarity and SOS Induction
Source: PLoS One. 2009 Feb 4;4(2):e4367. doi: 10.1371/journal.pone.0004367 (PMC2634741; doi:10.1371/journal.pone.0004367)
Supplement: Table S10 — Comparison of the genes regulated by H-NS (1) and by DNA supercoiling by Blot et al (2006) (2) (0.05 MB DOC) [file pone.0004367.s012.doc]

**Supplemental Table S10. Comparison of the genes regulated by H-NS ^(1)^ and by DNA supercoiling by Blot *et al* (2006) ^(2)^**

| **Gene** | **Blattner** | **Reg.^1^** | **Reg.^2^** | **Function** |
| --- | --- | --- | --- | --- |
| *yabP* | b0056 | Low-Exp | Hyp | orf; hypothetical protein |
| *adhC* | b0356 | High-Trans | Rel | alcohol dehydrogenase class III-- formaldehyde dehydrogenase; glutathione-dependent |
| *rhsD* | b0497 | Low-Exp | Hyp | rhsD protein in rhs element |
| *ycaL* | b0909 | High-Exp | Hyp | putative heat shock protein |
| *trpE* | b1264 | High-Exp | Hyp | anthranilate synthase component I |
| *fdnI* | b1476 | Low-Stat | Rel | formate dehydrogenase-N; nitrate-inducible; cytochrome B556(Fdn) gamma subunit |
| *b1625* | b1625 | Low-Exp | Hyp | orf; hypothetical protein |
| *ydiA* | b1703 | Low-Exp | Hyp | orf; hypothetical protein |
| *cheY* | b1882 | High-Trans | Rel | chemotaxis regulator transmits chemoreceptor signals to flagelllar motor components |
| *menF* | b2265 | Low-Exp | Hyp | isochorismate hydroxymutase 2; menaquinone biosynthesis |
| *srmB* | b2576 | Low-Stat | Hyp | ATP-dependent RNA helicase |
| *clpB* | b2592 | High-Exp | Rel | heat shock protein |
| *recN* | b2616 | Low-Exp | Rel | protein used in recombination and DNA repair |
| *b2689* | b2689 | High-Trans | Hyp | orf; hypothetical protein |
| *dsbC* | b2893 | Low-Exp | Hyp | protein disulfide isomerase II |
| *degS* | b3235 | High-Trans/High-Stat | Hyp | protease |
| *htrL* | b3618 | Low-Exp | Rel | involved in lipopolysaccharide biosynthesis |
| *kdtA* | b3633 | High-Trans | Hyp | 3-deoxy-D-manno-octulosonic-acid transferase (KDO transferase) |
| *ibpA* | b3687 | High-Stat | Rel | heat shock protein |
| *yjdA* | b4109 | Low-Exp | Hyp | putative vimentin |
