# Supplementary material for: The HU Regulon Is Composed of Genes Responding to Anaerobiosis, Acid Stress, High Osmolarity and SOS Induction
Source: PLoS One. 2009 Feb 4;4(2):e4367. doi: 10.1371/journal.pone.0004367 (PMC2634741; doi:10.1371/journal.pone.0004367)
Supplement: Table S11 — Comparison of the genes regulated by H-NS (1) and by DNA supercoiling by Peter et al (2004) (2) (0.09 MB DOC) [file pone.0004367.s013.doc]

**Supplemental Table S11. Comparison of the genes regulated by H-NS ^(1)^ and by DNA supercoiling by Peter *et al* (2004) ^(2)^**

| **Gene** | **Blattner** | **Reg.^1^** | **Reg.^2^** | **Function** |
| --- | --- | --- | --- | --- |
| *fixA* | b0041 | Low-Exp/Low-Trans | Rel | probable flavoprotein subunit; carnitine metabolism |
| *fixC* | b0043 | Low-Trans | Rel | flavoprotein-- electron transport |
| *yabJ* | b0066 | High-Stat | Rel | putative ATP-binding component of a transport system |
| *accA* | b0185 | Low-Stat | Hyp | acetylCoA carboxylase; carboxytransferase component; alpha subunit |
| *pepD* | b0237 | Low-Stat | Hyp | aminoacyl-histidine dipeptidase (peptidase D) |
| *insB;2* | b0264 | High-Trans | Hyp | IS1 protein InsB |
| *yajD* | b0410 | High-Exp | Hyp | orf; hypothetical protein |
| *nfnB* | b0578 | Low-Trans | Hyp | oxygen-insensitive NAD(P)H nitroreductase |
| *rna* | b0611 | High-Exp | Hyp | RNase I; cleaves phosphodiester bond between any two nucleotides |
| *citC* | b0618 | Low-Exp | Rel | citrate lyase synthetase (citrate (pro-3S)-lyase ligase |
| *citA* | b0619 | Low-Exp | Rel | putative sensor-type protein |
| *crcA* | b0622 | Low-Exp | Rel | orf; hypothetical protein |
| *msbA* | b0914 | Low-Exp | Hyp | ATP-binding transport protein-- multicopy suppressor of htrB |
| *kdsB* | b0918 | High-Exp/Low-Trans | Hyp | CTP:CMP-3-deoxy-D-manno-octulosonate transferase |
| *ycbY* | b0948 | High-Trans | Hyp | putative oxidoreductase |
| *ycfL* | b1104 | High-Stat | Hyp | orf; hypothetical protein |
| *mfd* | b1114 | High-Exp | Hyp | transcription-repair coupling factor-- mutation frequency decline |
| *b1169* | b1169 | Low-Exp/Low-Trans | Rel | putative ATP-binding component of a transport system |
| *b1330* | b1330 | Low-Exp/Low-Trans | Rel | orf; hypothetical protein |
| *b1501* | b1501 | Low-Exp | Rel | putative oxidoreductase; major subunit |
| *ydeH* | b1535 | Low-Exp | Rel | orf; hypothetical protein |
| *b1628* | b1628 | Low-Exp | Rel | orf; hypothetical protein |
| *ydgR* | b1634 | High-Trans | Hyp | putative transport protein |
| *gloA* | b1651 | High-Trans | Hyp | lactoylglutathione lyase |
| *ydiA* | b1703 | Low-Exp | Hyp | orf; hypothetical protein |
| *b1721* | b1721 | Low-Trans | Rel | orf; hypothetical protein |
| *b2253* | b2253 | Low-Exp | Rel | putative enzyme |
| *b2254* | b2254 | Low-Exp | Rel | putative sugar transferase |
| *lrhA* | b2289 | Low-Exp | Rel | NADH dehydrogenase transcriptional regulator; LysR family |
| *xapB* | b2406 | Low-Trans | Rel | xanthosine permease |
| *yfiC* | b2575 | Low-Exp | Hyp | putative enzyme |
| *b2682* | b2682 | High-Stat | Hyp | orf; hypothetical protein |
| *aas* | b2836 | Low-Stat | Hyp | 2-acyl-glycerophospho-ethanolamine acyltransferase-- acyl-acyl-carrier protein synthetase |
| *tdcR* | b3119 | High-Exp | Rel | threonine dehydratase operon activator protein |
| *uspA* | b3495 | High-Exp | Hyp | universal stress protein-- broad regulatory function? |
| *yiaU* | b3585 | Low-Exp | Rel | putative transcriptional regulator LYSR-type |
| *htrL* | b3618 | Low-Exp | Rel | involved in lipopolysaccharide biosynthesis |
| *rfe* | b3784 | High-Trans | Hyp | UDP-GlcNAc:undecaprenylphosphate GlcNAc-1-phosphate transferase-- synthesis of enterobacterial common antigen (ECA) |
| *fpr* | b3924 | High-Exp | Hyp | ferredoxin-NADP reductase |
| *yjaD* | b3996 | Low-Stat | Hyp | orf; hypothetical protein |
| *yjbC* | b4022 | High-Trans | Hyp | orf; hypothetical protein |
| *yjeS* | b4166 | High-Exp | Rel | orf; hypothetical protein |
| *ytfM* | b4220 | High-Stat | Hyp | orf; hypothetical protein |
| *fimI* | b4315 | Low-Stat | Hyp | fimbrial protein |
| *fimC* | b4316 | Low-Exp | Hyp | periplasmic chaperone; required for type 1 fimbriae |
| *mcrC* | b4345 | Low-Exp | Rel | component of McrBC 5-methylcytosine restriction system; expands range of sequences restricted |
| *serB* | b4388 | Low-Trans/Low-Stat | Hyp | 3-phosphoserine phosphatase |
