# Supplementary material for: The HU Regulon Is Composed of Genes Responding to Anaerobiosis, Acid Stress, High Osmolarity and SOS Induction
Source: PLoS One. 2009 Feb 4;4(2):e4367. doi: 10.1371/journal.pone.0004367 (PMC2634741; doi:10.1371/journal.pone.0004367)
Supplement: Table S12 — Comparison of the genes regulated by FIS (1) and by DNA supercoiling by Blot et al (2006) (2) (0.07 MB DOC) [file pone.0004367.s014.doc]

**Supplemental Table S12. Comparison of the genes regulated by FIS ^(1)^ and by DNA supercoiling by Blot *et al* (2006) ^(2)^**

| **Gene** | **Blattner** | **Reg.^1^** | **Reg.^2^** | **Function** |
| --- | --- | --- | --- | --- |
| *polB* | b0060 | High-Trans/Low-Stat | Rel | DNA polymerase II |
| *yafK* | b0224 | Low-Exp | Hyp | orf; hypothetical protein |
| *ybaN* | b0468 | High-Exp | Hyp | putative gene 58 |
| *rhsD* | b0497 | High-Stat | Hyp | rhsD protein in rhs element |
| *ybeD* | b0631 | Low-Exp | Rel | orf; hypothetical protein |
| *uvrB* | b0779 | High-Trans | Rel | DNA repair-- excision nuclease subunit B |
| *smtA* | b0921 | High-Exp | Hyp | S-adenosylmethionine-dependent methyltransferase |
| *mukE* | b0923 | High-Exp | Hyp | orf; hypothetical protein |
| *sulA* | b0958 | Low-Exp | Hyp | suppressor of lon-- inhibits cell division and ftsZ ring formation |
| *rimJ* | b1066 | High-Stat | Hyp | acetylation of N-terminal alanine of 30S ribosomal subunit protein S5 |
| *flgJ* | b1081 | High-Exp | Rel | flagellar biosynthesis |
| *ymjA* | b1295 | Low-Exp | Hyp | orf; hypothetical protein |
| *cspF* | b1558 | Low-Exp | Hyp | cold shock protein |
| *ydiA* | b1703 | Low-Exp | Hyp | orf; hypothetical protein |
| *b1832* | b1832 | High-Exp | Hyp | orf; hypothetical protein |
| *holE* | b1842 | Low-Exp | Hyp | DNA polymerase III; theta subunit |
| *yebL* | b1857 | Low-Stat | Hyp | putative adhesin |
| *yecN* | b1869 | Low-Exp | Hyp | orf; hypothetical protein |
| *yeeT* | b2003 | Low-Stat | Rel | orf; hypothetical protein |
| *spr* | b2175 | High-Exp | Hyp | putative lipoprotein |
| *purC* | b2476 | High-Trans | Hyp | phosphoribosylaminoimidazole-succinocarboxamide synthetase = SAICAR synthetase |
| *yfhG* | b2555 | Low-Stat | Hyp | putative alpha helix protein |
| *srmB* | b2576 | Low-Exp | Hyp | ATP-dependent RNA helicase |
| *clpB* | b2592 | Low-Stat | Rel | heat shock protein |
| *recN* | b2616 | Low-Exp | Rel | protein used in recombination and DNA repair |
| *ygaH* | b2683 | High-Exp | Hyp | orf; hypothetical protein |
| *gcvH* | b2904 | High-Exp | Hyp | in glycine cleavage complex; carrier of aminomethyl moiety via covalently bound lipoyl cofactor |
| *mutY* | b2961 | Low-Exp | Hyp | adenine glycosylase-- G.C --> T.A transversions |
| *yraM* | b3147 | Low-Stat | Hyp | putative glycosylase |
| *yhgI* | b3414 | Low-Exp | Rel | orf; hypothetical protein |
| *htrL* | b3618 | Low-Stat | Rel | involved in lipopolysaccharide biosynthesis |
| *ibpA* | b3687 | Low-Exp | Rel | heat shock protein |
| *hemC* | b3805 | Low-Stat | Hyp | porphobilinogen deaminase = hydroxymethylbilane synthase |
| *creB* | b4398 | Low-Stat | Hyp | catabolic regulation response regulator |
