# Supplementary material for: The HU Regulon Is Composed of Genes Responding to Anaerobiosis, Acid Stress, High Osmolarity and SOS Induction
Source: PLoS One. 2009 Feb 4;4(2):e4367. doi: 10.1371/journal.pone.0004367 (PMC2634741; doi:10.1371/journal.pone.0004367)
Supplement: Table S13 — Comparison of the genes regulated by FIS (1) and by DNA supercoiling by Peter et al (2004) (2) (0.10 MB DOC) [file pone.0004367.s015.doc]

**Supplemental Table S13. Comparison of the genes regulated by FIS ^(1)^ and by DNA supercoiling by Peter *et al* (2004) ^(2)^**

| **Gene** | **Blattner** | **Reg.^1^** | **Reg.^2^** | **Function** |
| --- | --- | --- | --- | --- |
| *lspA* | b0027 | Low-Exp | Hyp | prolipoprotein signal peptidase (SPase II) |
| *dniR* | b0211 | Low-Exp | Hyp | transcriptional regulator for nitrite reductase (cytochrome c552) |
| *gloB* | b0212 | High-Exp/Low-Stat | Hyp | probable hydroxyacylglutathione hydrolase |
| *yafK* | b0224 | Low-Exp | Hyp | orf; hypothetical protein |
| *pepD* | b0237 | Low-Stat | Hyp | aminoacyl-histidine dipeptidase (peptidase D) |
| *yahK* | b0325 | High-Stat | Rel | putative oxidoreductase |
| *yajK* | b0423 | Low-Exp/High-Trans | Hyp | putative oxidoreductase |
| *nmpC* | b0553 | High-Exp | Hyp | outer membrane porin protein-- locus of qsr prophage |
| *ybcU* | b0557 | High-Stat | Hyp | bacteriophage lambda Bor protein homolog |
| *citA* | b0619 | Low-Exp | Rel | putative sensor-type protein |
| *yleA* | b0661 | Low-Exp | Rel | orf; hypothetical protein |
| *ybhH* | b0769 | Low-Exp | Rel | orf; hypothetical protein |
| *smtA* | b0921 | High-Exp | Hyp | S-adenosylmethionine-dependent methyltransferase |
| *mukE* | b0923 | High-Exp | Hyp | orf; hypothetical protein |
| *b0947* | b0947 | High-Exp | Hyp | orf; hypothetical protein |
| *rimJ* | b1066 | High-Stat | Hyp | acetylation of N-terminal alanine of 30S ribosomal subunit protein S5 |
| *ycfD* | b1128 | Low-Exp | Hyp | orf; hypothetical protein |
| *cls* | b1249 | High-Exp/High-Trans | Hyp | cardiolipin synthase; a major membrane phospholipid-- novobiocin sensitivity |
| *b1330* | b1330 | High-Trans | Rel | orf; hypothetical protein |
| *adhP* | b1478 | Low-Exp | Rel | alcohol dehydrogenase |
| *manA* | b1613 | Low-Exp | Hyp | mannose-6-phosphate isomerase |
| *ydiA* | b1703 | Low-Exp | Hyp | orf; hypothetical protein |
| *b1706* | b1706 | Low-Exp | Hyp | orf; hypothetical protein |
| *b1809* | b1809 | Low-Exp | Hyp | orf; hypothetical protein |
| *b1827* | b1827 | High-Exp | Hyp | putative regulator |
| *b1832* | b1832 | High-Exp | Hyp | orf; hypothetical protein |
| *holE* | b1842 | Low-Exp | Hyp | DNA polymerase III; theta subunit |
| *zwf* | b1852 | High-Exp | Hyp | glucose-6-phosphate dehydrogenase |
| *shiA* | b1981 | High-Exp | Hyp | putative transport protein; shikimate |
| *yojN* | b2216 | Low-Exp | Hyp | putative 2-component sensor protein |
| *b2253* | b2253 | Low-Stat | Rel | putative enzyme |
| *lrhA* | b2289 | Low-Stat | Rel | NADH dehydrogenase transcriptional regulator; LysR family |
| *purC* | b2476 | High-Trans | Rel | phosphoribosylaminoimidazole-succinocarboxamide synthetase = SAICAR synthetase |
| *hcaR* | b2537 | Low-Exp | Hyp | transcriptional activator of hca cluster |
| *ygaH* | b2683 | High-Exp | Hyp | orf; hypothetical protein |
| *yqaB* | b2690 | Low-Exp | Hyp | putative phosphatase |
| *recJ* | b2892 | Low-Exp | Hyp | ssDNA exonuclease; 5' --> 3' specific |
| *tdcB* | b3117 | High-Exp | Rel | threonine dehydratase; catabolic |
| *yraM* | b3147 | Low-Stat | Hyp | putative glycosylase |
| *yraN* | b3148 | Low-Exp | Hyp | orf; hypothetical protein |
| *htrL* | b3618 | Low-Stat | Rel | involved in lipopolysaccharide biosynthesis |
| *rfaI* | b3627 | Low-Stat | Rel | UDP-D-galactose:(glucosyl)lipopolysaccharide- alpha-1;3-D-galactosyltransferase |
| *yicH* | b3655 | High-Stat | Hyp | orf; hypothetical protein |
| *dnaN* | b3701 | Low-Exp | Rel | DNA polymerase III; beta-subunit |
| *glmU* | b3730 | High-Exp | Hyp | N-acetyl glucosamine-1-phosphate uridyltransferase |
| *yifE* | b3764 | High-Exp | Hyp | orf; hypothetical protein |
| *rfe* | b3784 | High-Exp | Hyp | UDP-GlcNAc:undecaprenylphosphate GlcNAc-1-phosphate transferase-- synthesis of enterobacterial common antigen (ECA) |
| *yifK* | b3795 | High-Exp | Hyp | putative amino acid/amine transport protein |
| *fpr* | b3924 | High-Exp | Hyp | ferredoxin-NADP reductase |
| *yjaE* | b3995 | Low-Stat | Hyp | putative transcriptional regulator |
| *yjaD* | b3996 | Low-Exp | Hyp | orf; hypothetical protein |
| *ytfM* | b4220 | Low-Trans | Hyp | orf; hypothetical protein |
| *fimI* | b4315 | Low-Exp | Hyp | fimbrial protein |
| *fimC* | b4316 | Low-Exp/Low-Stat | Hyp | periplasmic chaperone; required for type 1 fimbriae |
| *mcrB* | b4346 | High-Exp/High-Trans | Rel | component of McrBC 5-methylcytosine restriction system |
| *deoC* | b4381 | High-Exp/High-Stat | Rel | 2-deoxyribose-5-phosphate aldolase |
