# Supplementary material for: The HU Regulon Is Composed of Genes Responding to Anaerobiosis, Acid Stress, High Osmolarity and SOS Induction
Source: PLoS One. 2009 Feb 4;4(2):e4367. doi: 10.1371/journal.pone.0004367 (PMC2634741; doi:10.1371/journal.pone.0004367)
Supplement: Table S14 — Comparison of the genes regulated by FIS (1) and by DNA supercoiling by Blot et al (2006) (2) and Peter et al (2004) (3) (0.03 MB DOC) [file pone.0004367.s016.doc]

**Supplemental Table S14. Comparison of the genes regulated by FIS ^(1)^ and by DNA supercoiling by Blot *et al* (2006) ^(2)^ and Peter et al (2004) ^(3)^**

| **Gene** | **Blattner** | **Reg.^1^** | **Reg.^2^** | **Reg.^2^** | **Function** |
| --- | --- | --- | --- | --- | --- |
| *yebR* | b1832 | High-Exp | Hyp | Hyp | orf; hypothetical protein |
