# Supplementary material for: The HU Regulon Is Composed of Genes Responding to Anaerobiosis, Acid Stress, High Osmolarity and SOS Induction
Source: PLoS One. 2009 Feb 4;4(2):e4367. doi: 10.1371/journal.pone.0004367 (PMC2634741; doi:10.1371/journal.pone.0004367)
Supplement: Table S15 — Comparison of the genes regulated by HU (1) and by H-NS (Blot et al, 2006 (2)) (0.14 MB DOC) [file pone.0004367.s017.doc]

**Supplemental Table S15. Comparison of the genes regulated by HU ^(1)^ and by H-NS (Blot *et al,* 2006 ^(2)^)**

| **Gene** | **Blattner** | **Reg.^1^** | **Reg.^2^** | **Function** |
| --- | --- | --- | --- | --- |
| *entC* | b0593 | [Cluster2] | Low-Trans | isochorismate hydroxymutase 2; enterochelin biosynthesis |
| *entA* | b0596 | [Cluster2] | High-Exp/High-Trans | 2;3-dihydro-2;3-dihydroxybenzoate dehydrogenase; enterochelin biosynthesis |
| *hspQ* | b0966 | [Cluster2] | High-Stat | orf; hypothetical protein |
| *yciE* | b1257 | [Cluster2] | Low-Exp | orf; hypothetical protein |
| *sra* | b1480 | [Cluster2] | High-Trans | 30S ribosomal subunit protein S22 |
| *bdm* | b1481 | [Cluster2] | Low-Stat | orf; hypothetical protein |
| *yddV* | b1490 | [Cluster2] | Low-Exp | orf; hypothetical protein |
| *gadC* | b1492 | [Cluster2] | Low-Exp/Low-Stat | acid sensitivity protein; putative transporter |
| *ydeI* | b1536 | [Cluster2] | Low-Exp/Low-Stat | orf; hypothetical protein |
| *cfa* | b1661 | [Cluster2] | High-Exp | cyclopropane fatty acyl phospholipid synthase |
| *fic* | b3361 | [Cluster2] | High-Exp | induced in stationary phase; recognized by rpoS; affects cell division |
| *yhhA* | b3448 | [Cluster2] | High-Trans | orf; hypothetical protein |
| *yhiM* | b3491 | [Cluster2] | Low-Exp/Low-Trans/Low-Stat | orf; hypothetical protein |
| *slp* | b3506 | [Cluster2] | Low-Exp/Low-Trans | outer membrane protein induced after carbon starvation |
| *dctR* | b3507 | [Cluster2] | Low-Exp | orf; hypothetical protein |
| *hdeB* | b3509 | [Cluster2] | Low-Stat | orf; hypothetical protein |
| *hdeA* | b3510 | [Cluster2] | Low-Stat | orf; hypothetical protein |
| *hdeD* | b3511 | [Cluster2] | Low-Exp | orf; hypothetical protein |
| *gadE* | b3512 | [Cluster2] | Low-Exp | orf; hypothetical protein |
| *gadW* | b3515 | [Cluster2] | Low-Exp | putative ARAC-type regulatory protein |
| *gadX* | b3516 | [Cluster2] | Low-Exp | putative ARAC-type regulatory protein |
| *gadA* | b3517 | [Cluster2] | Low-Exp/Low-Stat | glutamate decarboxylase isozyme |
| *dmsB* | b0895 | [Cluster4] | High-Exp | anaerobic dimethyl sulfoxide reductase subunit B |
| *yfeX* | b2431 | [Cluster4] | Low-Trans | orf; hypothetical protein |
| *srlB* | b2704 | [Cluster4] | High-Trans | PTS system; glucitol/sorbitol-specific enzyme IIA component |
| *hypB* | b2727 | [Cluster4] | High-Exp | guanine-nucleotide binding protein; functions as nickel donor for large subunit of hydrogenase 3 |
| *garD* | b3128 | [Cluster4] | Low-Trans | putative hydrolase |
| *nanA* | b3225 | [Cluster4] | High-Trans | N-acetylneuraminate lyase (aldolase)-- catabolism of sialic acid-- not K-12? |
| *feoB* | b3409 | [Cluster4] | High-Exp | ferrous iron transport protein B |
| *yhjX* | b3547 | [Cluster4] | High-Exp | putative resistance protein |
| *pfkA* | b3916 | [Cluster4] | High-Exp | 6-phosphofructokinase I |
| *yjjI* | b4380 | [Cluster4] | High-Exp | orf; hypothetical protein |
| *paaE* | b1392 | [Cluster5] | High-Trans | putative oxidoreductase |
| *pps* | b1702 | [Cluster5] | Low-Trans | phosphoenolpyruvate synthase |
| *cysP* | b2425 | [Cluster5] | High-Exp | thiosulfate binding protein |
| *iscS* | b2530 | [Cluster5] | High-Trans | putative aminotransferase |
| *clpB* | b2592 | [Cluster5] | High-Exp | heat shock protein |
| *proV* | b2677 | [Cluster5] | Low-Exp/Low-Trans | ATP-binding component of transport system for glycine; betaine and proline |
| *proW* | b2678 | [Cluster5] | Low-Exp | high-affinity transport system for glycine betaine and proline |
| *cysH* | b2762 | [Cluster5] | High-Exp | 3'-phosphoadenosine 5'-phosphosulfate reductase |
| *ibpB* | b3686 | [Cluster5] | High-Stat | heat shock protein |
| *ibpA* | b3687 | [Cluster5] | High-Stat | heat shock protein |
| *groS* | b4142 | [Cluster5] | High-Exp | GroES; 10 Kd chaperone binds to Hsp60 in pres. Mg-ATP; suppressing its ATPase activity |
| *groL* | b4143 | [Cluster5] | High-Exp | GroEL; chaperone Hsp60; peptide-dependent ATPase; heat shock protein |
| *yjhS* | b4309 | [Cluster5] | High-Trans | orf; hypothetical protein |
| *fimI* | b4315 | [Cluster5] | Low-Stat | fimbrial protein |
| *fimC* | b4316 | [Cluster5] | Low-Exp | periplasmic chaperone; required for type 1 fimbriae |
| *ymfL* | b1147 | [Cluster6] | High-Trans | orf; hypothetical protein |
| *recX* | b2698 | [Cluster6] | Low-Exp | regulator; OraA protein |
| *recA* | b2699 | [Cluster6] | Low-Exp/Low-Trans | DNA strand exchange and renaturation; DNA-dependent ATPase; DNA- and ATP-dependent coprotease |
| *bglG* | b3723 | [Cluster6] | Low-Exp | positive regulation of bgl operon |
| *rihC* | b0030 | [Cluster7] | High-Exp | orf; hypothetical protein |
| *aceE* | b0114 | [Cluster7] | Low-Trans | pyruvate dehydrogenase (decarboxylase component) |
| *cydA* | b0733 | [Cluster7] | Low-Trans | cytochrome d terminal oxidase; polypeptide subunit I |
| *pflB* | b0903 | [Cluster7] | High-Exp | formate acetyltransferase 1 |
| *flgB* | b1073 | [Cluster7] | Low-Trans | flagellar biosynthesis; cell-proximal portion of basal-body rod |
| *flgC* | b1074 | [Cluster7] | High-Exp | flagellar biosynthesis; cell-proximal portion of basal-body rod |
| *flgD* | b1075 | [Cluster7] | High-Exp | flagellar biosynthesis; initiation of hook assembly |
| *ompW* | b1256 | [Cluster7] | High-Exp | putative outer membrane protein |
| *fdnI* | b1476 | [Cluster7] | Low-Stat | formate dehydrogenase-N; nitrate-inducible; cytochrome B556(Fdn) gamma subunit |
| *ydhY* | b1674 | [Cluster7] | Low-Exp | putative oxidoreductase; Fe-S subunit |
| *yeaU* | b1800 | [Cluster7] | Low-Exp/Low-Stat | putative tartrate dehydrogenase |
| *ftnA* | b1905 | [Cluster7] | Low-Exp | cytoplasmic ferritin (an iron storage protein) |
| *fruB* | b2169 | [Cluster7] | Low-Exp | PTS system; fructose-specific IIA/fpr component |
| *yqeC* | b2876 | [Cluster7] | Low-Exp | orf; hypothetical protein |
| *ansB* | b2957 | [Cluster7] | Low-Trans | periplasmic L-asparaginase II |
| *ygjR* | b3087 | [Cluster7] | High-Exp | orf; hypothetical protein |
| *tdcD* | b3115 | [Cluster7] | Low-Trans | putative kinase |
| *tdcC* | b3116 | [Cluster7] | Low-Stat | anaerobically inducible L-threonine; L-serine permease |
| *asnA* | b3744 | [Cluster7] | High-Exp | asparagine synthetase A |
| *katG* | b3942 | [Cluster7] | High-Exp | catalase-- hydroperoxidase HPI(I) |
| *cadA* | b4131 | [Cluster7] | Low-Exp | lysine decarboxylase 1 |
| *frdD* | b4151 | [Cluster7] | High-Exp | fumarate reductase; anaerobic; membrane anchor polypeptide |
| *treC* | b4239 | [Cluster7] | High-Exp | trehalase 6-P hydrolase |
| *treB* | b4240 | [Cluster7] | High-Exp | PTS system enzyme II; trehalose specific |
| *pyrB* | b4245 | [Cluster7] | High-Exp/High-Trans | aspartate carbamoyltransferase; catalytic subunit |
