# Supplementary material for: The HU Regulon Is Composed of Genes Responding to Anaerobiosis, Acid Stress, High Osmolarity and SOS Induction
Source: PLoS One. 2009 Feb 4;4(2):e4367. doi: 10.1371/journal.pone.0004367 (PMC2634741; doi:10.1371/journal.pone.0004367)
Supplement: Table S16 — Comparison of the genes regulated by HU (1) and by FIS (Blot et al, 2006 (2)) (0.16 MB DOC) [file pone.0004367.s018.doc]

**Supplemental Table S16. Comparison of the genes regulated by HU ^(1)^ and by FIS (Blot *et al,* 2006 ^(2)^)**

| **Gene** | **Blattner** | **Reg.^1^** | **Reg.^2^** | **Function** |
| --- | --- | --- | --- | --- |
| *ybaT* | b0486 | [Cluster2] | High-Trans | putative amino acid/amine transport protein |
| *entA* | b0596 | [Cluster2] | High-Trans | 2;3-dihydro-2;3-dihydroxybenzoate dehydrogenase; enterochelin biosynthesis |
| *ybiM* | b0806 | [Cluster2] | Low-Exp | orf; hypothetical protein |
| *hspQ* | b0966 | [Cluster2] | Low-Exp | orf; hypothetical protein |
| *wrbA* | b1004 | [Cluster2] | High-Exp/High-Stat | trp repressor binding protein-- affects association of trp repressor and operator |
| *grxB* | b1064 | [Cluster2] | High-Stat | glutaredoxin 2 |
| *ymgA* | b1165 | [Cluster2] | Low-Exp/High-Stat | orf; hypothetical protein |
| *ymgC* | b1167 | [Cluster2] | High-Stat | orf; hypothetical protein |
| *osmB* | b1283 | [Cluster2] | Low-Exp | osmotically inducible lipoprotein |
| *adhP* | b1478 | [Cluster2] | Low-Exp | alcohol dehydrogenase |
| *bdm* | b1481 | [Cluster2] | Low-Exp | orf; hypothetical protein |
| *gadC* | b1492 | [Cluster2] | Low-Exp/High-Trans | acid sensitivity protein; putative transporter |
| *ydeI* | b1536 | [Cluster2] | High-Trans/High-Stat | orf; hypothetical protein |
| *ydiH* | b1685 | [Cluster2] | Low-Exp | orf; hypothetical protein |
| *hchA* | b1967 | [Cluster2] | Low-Exp/High-Trans | orf; hypothetical protein |
| *fbaB* | b2097 | [Cluster2] | Low-Exp | orf; hypothetical protein |
| *talA* | b2464 | [Cluster2] | High-Stat | transaldolase A |
| *yqjE* | b3099 | [Cluster2] | High-Stat | orf; hypothetical protein |
| *slp* | b3506 | [Cluster2] | High-Trans/High-Stat | outer membrane protein induced after carbon starvation |
| *dctR* | b3507 | [Cluster2] | High-Trans | orf; hypothetical protein |
| *hdeD* | b3511 | [Cluster2] | High-Trans | orf; hypothetical protein |
| *gadE* | b3512 | [Cluster2] | High-Trans | orf; hypothetical protein |
| *gadW* | b3515 | [Cluster2] | High-Trans | putative ARAC-type regulatory protein |
| *aceA* | b4015 | [Cluster2] | Low-Exp | isocitrate lyase |
| *aceK* | b4016 | [Cluster2] | Low-Stat | isocitrate dehydrogenase kinase/phosphatase |
| *ytfK* | b4217 | [Cluster2] | High-Stat | orf; hypothetical protein |
| *osmY* | b4376 | [Cluster2] | High-Stat | hyperosmotically inducible periplasmic protein |
| *galK* | b0757 | [Cluster4] | Low-Exp | galactokinase |
| *dmsA* | b0894 | [Cluster4] | High-Exp/High-Trans | anaerobic dimethyl sulfoxide reductase subunit A |
| *dhaL* | b1199 | [Cluster4] | High-Exp | putative dihydroxyacetone kinase (EC 2.7.1.2) |
| *narG* | b1224 | [Cluster4] | High-Stat | nitrate reductase 1; alpha subunit |
| *narJ* | b1226 | [Cluster4] | Low-Stat | nitrate reductase 1; delta subunit; assembly function |
| *napD* | b2207 | [Cluster4] | High-Exp | orf; hypothetical protein |
| *napF* | b2208 | [Cluster4] | High-Exp | ferredoxin-type protein: electron transfer |
| *gutQ* | b2708 | [Cluster4] | High-Exp | orf; hypothetical protein |
| *hypB* | b2727 | [Cluster4] | High-Exp/High-Trans | guanine-nucleotide binding protein; functions as nickel donor for large subunit of hydrogenase 3 |
| *hypC* | b2728 | [Cluster4] | High-Exp | pleiotrophic effects on 3 hydrogenase isozymes |
| *nanA* | b3225 | [Cluster4] | High-Trans | N-acetylneuraminate lyase (aldolase)-- catabolism of sialic acid-- not K-12? |
| *udp* | b3831 | [Cluster4] | High-Exp | uridine phosphorylase |
| *nrfA* | b4070 | [Cluster4] | High-Exp | periplasmic cytochrome c(552): plays a role in nitrite reduction |
| *yjdK* | b4128 | [Cluster4] | Low-Stat | orf; hypothetical protein |
| *yjiX* | b4353 | [Cluster4] | High-Exp | orf; hypothetical protein |
| *yjiY* | b4354 | [Cluster4] | High-Exp | putative carbon starvation protein |
| *yjjI* | b4380 | [Cluster4] | High-Exp | orf; hypothetical protein |
| *cyoB* | b0431 | [Cluster5] | High-Exp | cytochrome o ubiquinol oxidase subunit I |
| *sdhB* | b0724 | [Cluster5] | High-Stat | succinate dehydrogenase; iron sulfur protein |
| *sucA* | b0726 | [Cluster5] | High-Stat | 2-oxoglutarate dehydrogenase (decarboxylase component) |
| *pps* | b1702 | [Cluster5] | Low-Exp | phosphoenolpyruvate synthase |
| *fliY* | b1920 | [Cluster5] | High-Stat | putative periplasmic binding transport protein |
| *yedE* | b1929 | [Cluster5] | Low-Exp | putative transport system permease protein |
| *iscS* | b2530 | [Cluster5] | Low-Exp | putative aminotransferase |
| *clpB* | b2592 | [Cluster5] | Low-Stat | heat shock protein |
| *proW* | b2678 | [Cluster5] | Low-Trans | high-affinity transport system for glycine betaine and proline |
| *sdaC* | b2796 | [Cluster5] | Low-Exp | probable serine transporter |
| *yhcH* | b3221 | [Cluster5] | High-Exp | orf; hypothetical protein |
| *ibpB* | b3686 | [Cluster5] | Low-Stat | heat shock protein |
| *ibpA* | b3687 | [Cluster5] | Low-Exp | heat shock protein |
| *hslU* | b3931 | [Cluster5] | Low-Exp | heat shock protein hslVU; ATPase subunit; homologous to chaperones |
| *hslV* | b3932 | [Cluster5] | Low-Exp | heat shock protein hslVU; proteasome-related peptidase subunit |
| *groL* | b4143 | [Cluster5] | Low-Stat | GroEL; chaperone Hsp60; peptide-dependent ATPase; heat shock protein |
| *yjhT* | b4310 | [Cluster5] | Low-Exp/Low-Stat | orf; hypothetical protein |
| *fimI* | b4315 | [Cluster5] | Low-Exp | fimbrial protein |
| *fimC* | b4316 | [Cluster5] | Low-Exp/Low-Stat | periplasmic chaperone; required for type 1 fimbriae |
| *sulA* | b0958 | [Cluster6] | Low-Exp | suppressor of lon-- inhibits cell division and ftsZ ring formation |
| *cspG* | b0990 | [Cluster6] | Low-Stat | homolog of Salmonella cold shock protein |
| *dinI* | b1061 | [Cluster6] | Low-Exp | damage-inducible protein I |
| *mqsR* | b3022 | [Cluster6] | High-Stat | orf; hypothetical protein |
| *sodA* | b3908 | [Cluster6] | High-Exp | superoxide dismutase; manganese |
| *fsaB* | b3946 | [Cluster6] | Low-Stat | putative transaldolase |
| *fecB* | b4290 | [Cluster6] | High-Exp | citrate-dependent iron transport; periplasmic protein |
| *fecA* | b4291 | [Cluster6] | Low-Stat | outer membrane receptor-- citrate-dependent iron transport; outer membrane receptor |
| *carB* | b0033 | [Cluster7] | High-Exp | carbamoyl-phosphate synthase large subunit |
| *rihA* | b0651 | [Cluster7] | High-Exp | putative tRNA synthetase |
| *ycbJ* | b0919 | [Cluster7] | Low-Exp | orf; hypothetical protein |
| *flgB* | b1073 | [Cluster7] | Low-Stat | flagellar biosynthesis; cell-proximal portion of basal-body rod |
| *manZ* | b1819 | [Cluster7] | High-Stat | PTS enzyme IID; mannose-specific |
| *ftnA* | b1905 | [Cluster7] | Low-Exp | cytoplasmic ferritin (an iron storage protein) |
| *fruB* | b2169 | [Cluster7] | High-Trans | PTS system; fructose-specific IIA/fpr component |
| *glpA* | b2241 | [Cluster7] | High-Exp/High-Stat | sn-glycerol-3-phosphate dehydrogenase (anaerobic); large subunit |
| *upp* | b2498 | [Cluster7] | High-Trans/Low-Stat | uracil phosphoribosyltransferase |
| *yfiD* | b2579 | [Cluster7] | High-Exp | putative formate acetyltransferase |
| *gcvH* | b2904 | [Cluster7] | High-Exp | in glycine cleavage complex; carrier of aminomethyl moiety via covalently bound lipoyl cofactor |
| *glcB* | b2976 | [Cluster7] | Low-Exp | malate synthase G |
| *hybO* | b2997 | [Cluster7] | High-Exp | putative hydrogenase subunit |
| *tdcB* | b3117 | [Cluster7] | High-Exp | threonine dehydratase; catabolic |
| *pck* | b3403 | [Cluster7] | High-Stat | phosphoenolpyruvate carboxykinase |
| *malP* | b3417 | [Cluster7] | High-Exp | maltodextrin phosphorylase |
| *asnA* | b3744 | [Cluster7] | Low-Stat | asparagine synthetase A |
| *glnA* | b3870 | [Cluster7] | Low-Stat | glutamine synthetase |
| *aphA* | b4055 | [Cluster7] | Low-Trans | diadenosine tetraphosphatase |
| *fumB* | b4122 | [Cluster7] | High-Stat | fumarase B= fumarate hydratase Class I-- anaerobic isozyme |
| *dcuA* | b4138 | [Cluster7] | High-Exp | anaerobic dicarboxylate transport |
| *frdC* | b4152 | [Cluster7] | High-Exp | fumarate reductase; anaerobic; membrane anchor polypeptide |
| *pyrI* | b4244 | [Cluster7] | High-Exp | aspartate carbamoyltransferase; regulatory subunit |
