# Supplementary material for: The HU Regulon Is Composed of Genes Responding to Anaerobiosis, Acid Stress, High Osmolarity and SOS Induction
Source: PLoS One. 2009 Feb 4;4(2):e4367. doi: 10.1371/journal.pone.0004367 (PMC2634741; doi:10.1371/journal.pone.0004367)
Supplement: Table S17 — Comparison of the genes regulated by H-NS (1) and FIS(2) (Blot et al, 2006) (0.22 MB DOC) [file pone.0004367.s019.doc]

**Supplemental Table S17. Comparison of the genes regulated by H-NS ^(1)^ and FIS^(2)^ (Blot *et al,* 2006)**

| **Gene** | **Blattner** | **Reg.^1^** | **Reg.^2^** | **Function** |
| --- | --- | --- | --- | --- |
| *folK* | b0142 | Low-Stat | Low-Exp | 7;8-dihydro-6-hydroxymethylpterin- pyrophosphokinase |
| *pfs* | b0159 | High-Stat | Low-Exp | orf; hypothetical protein |
| *pepD* | b0237 | Low-Stat | Low-Stat | aminoacyl-histidine dipeptidase (peptidase D) |
| *betA* | b0311 | High-Trans | Low-Stat | choline dehydrogenase; a flavoprotein |
| *ddlA* | b0381 | Low-Stat | High-Trans | D-alanine-D-alanine ligase A |
| *proY* | b0402 | Low-Stat | Low-Stat | proline permease transport protein |
| *ybaD* | b0413 | High-Exp | Low-Exp | orf; hypothetical protein |
| *thiL* | b0417 | High-Trans | High-Trans | thiamin-monophosphate kinase |
| *rhsD* | b0497 | Low-Exp | High-Stat | rhsD protein in rhs element |
| *fdrA* | b0518 | Low-Trans | Low-Stat | involved in protein transport-- multicopy suppressor of dominant negative ftsH mutants |
| *ybcM* | b0546 | Low-Exp | High-Trans | putative ARAC-type regulatory protein |
| *rus* | b0550 | High-Trans | Low-Stat | endodeoxyribonuclease RUS (Holliday junction resolvase) |
| *entA* | b0596 | High-Exp/High-Trans | High-Trans | 2;3-dihydro-2;3-dihydroxybenzoate dehydrogenase; enterochelin biosynthesis |
| *citA* | b0619 | Low-Exp | Low-Exp | putative sensor-type protein |
| *citB* | b0620 | Low-Exp | Low-Trans | sequence similarity to Shigella regulator |
| *ybfD* | b0706 | Low-Stat | Low-Exp | putative DNA ligase |
| *ybgH* | b0709 | High-Trans | Low-Trans | putative transport protein |
| *ybhA* | b0766 | Low-Stat | Low-Stat | putative phosphatase |
| *mipB* | b0825 | High-Exp | High-Trans | putative transaldolase |
| *b0836* | b0836 | High-Exp | Low-Exp | putative receptor |
| *b0899* | b0899 | Low-Exp | Low-Exp | putative transport |
| *ompF* | b0929 | High-Exp/High-Trans | Low-Trans | outer membrane protein 1a (Ia--b--F) |
| *yccV* | b0966 | High-Stat | Low-Exp | orf; hypothetical protein |
| *b1016* | b1016 | High-Exp | High-Stat | orf; hypothetical protein |
| *ycdR* | b1023 | Low-Exp | Low-Stat | orf; hypothetical protein |
| *csgF* | b1038 | Low-Exp | High-Trans | curli production assembly/transport component; 2nd curli operon |
| *csgA* | b1042 | Low-Exp | Low-Exp | curlin major subunit; coiled surface structures-- cryptic |
| *flgB* | b1073 | Low-Trans | Low-Stat | flagellar biosynthesis; cell-proximal portion of basal-body rod |
| *ycfR* | b1112 | Low-Exp/High-Stat | High-Trans | orf; hypothetical protein |
| *b1171* | b1171 | Low-Exp | Low-Stat | orf; hypothetical protein |
| *b1202* | b1202 | Low-Exp/Low-Trans | Low-Exp | putative adhesion and penetration protein |
| *chaC* | b1218 | Low-Exp | Low-Exp | cation transport regulator |
| *ycjO* | b1311 | High-Exp | Low-Stat | putative binding-protein dependent transport protein |
| *b1329* | b1329 | Low-Exp | High-Exp | putative transport periplasmic protein |
| *b1330* | b1330 | Low-Exp/Low-Trans | High-Trans | orf; hypothetical protein |
| *b1400* | b1400 | High-Trans | Low-Exp | putative transferase |
| *ydcF* | b1414 | High-Trans | Low-Exp | orf; hypothetical protein |
| *b1472* | b1472 | Low-Stat | Low-Stat | putative outer membrane porin protein |
| *b1481* | b1481 | Low-Stat | Low-Exp | orf; hypothetical protein |
| *xasA* | b1492 | Low-Exp/Low-Stat | Low-Exp/High-Trans | acid sensitivity protein; putative transporter |
| *b1527* | b1527 | Low-Exp/High-Trans | High-Trans | orf; hypothetical protein |
| *ydeI* | b1536 | Low-Exp/Low-Stat | High-Trans/High-Stat | orf; hypothetical protein |
| *b1543* | b1543 | High-Exp | Low-Stat | putative transport protein |
| *relE* | b1563 | Low-Exp | Low-Exp | orf; hypothetical protein |
| *b1626* | b1626 | Low-Exp | Low-Exp | orf; hypothetical protein |
| *ynhD* | b1682 | High-Exp | High-Trans | putative ATP-binding component of a transport system |
| *ynhE* | b1683 | Low-Stat | High-Exp/High-Trans | orf; hypothetical protein |
| *ydiC* | b1684 | High-Exp | High-Exp | orf; hypothetical protein |
| *ppsA* | b1702 | Low-Trans | Low-Exp | phosphoenolpyruvate synthase |
| *ydiA* | b1703 | Low-Exp | Low-Exp | orf; hypothetical protein |
| *cstC* | b1748 | Low-Stat | High-Stat | acetylornithine delta-aminotransferase |
| *b1777* | b1777 | High-Trans | Low-Exp | orf; hypothetical protein |
| *yeaD* | b1780 | High-Exp | Low-Exp | orf; hypothetical protein |
| *pykA* | b1854 | Low-Trans | Low-Stat | pyruvate kinase II; glucose stimulated |
| *ruvA* | b1861 | Low-Exp | Low-Exp | Holliday junction helicase subunit B-- branch migration-- repair |
| *ftn* | b1905 | Low-Exp | Low-Exp | cytoplasmic ferritin (an iron storage protein) |
| *yedL* | b1932 | Low-Exp | Low-Exp | orf; hypothetical protein |
| *yedW* | b1969 | Low-Exp | High-Trans | putative 2-component transcriptional regulator |
| *udk* | b2066 | High-Trans | Low-Exp | uridine/cytidine kinase |
| *yohJ* | b2141 | High-Exp | Low-Stat | orf; hypothetical protein |
| *yeiK* | b2162 | Low-Exp | High-Trans | orf; hypothetical protein |
| *yeiN* | b2165 | Low-Stat | Low-Exp | orf; hypothetical protein |
| *yeiC* | b2166 | Low-Exp/Low-Trans/Low-Stat | Low-Exp | putative kinase |
| *fruK* | b2168 | Low-Exp | High-Trans | fructose-1-phosphate kinase |
| *fruB* | b2169 | Low-Exp | High-Trans | PTS system; fructose-specific IIA/fpr component |
| *b2253* | b2253 | Low-Exp | Low-Stat | putative enzyme |
| *menD* | b2264 | Low-Trans | Low-Exp | 2-oxoglutarate decarboxylase-- SHCHC synthase |
| *lrhA* | b2289 | Low-Exp | Low-Stat | NADH dehydrogenase transcriptional regulator; LysR family |
| *glk* | b2388 | High-Exp | High-Stat | glucokinase |
| *xapR* | b2405 | Low-Exp/Low-Stat | High-Trans | regulator for xapA |
| *b2475* | b2475 | High-Exp | High-Exp | orf; hypothetical protein |
| *hyfI* | b2489 | High-Exp | Low-Stat | hydrogenase 4 Fe-S subunit |
| *b2505* | b2505 | Low-Exp | High-Trans | putative outer membrane lipoprotein |
| *yfhF* | b2528 | High-Trans | Low-Exp | putative regulator |
| *yfhO* | b2530 | High-Trans | Low-Exp | putative aminotransferase |
| *suhB* | b2533 | Low-Exp | Low-Trans | enhances synthesis of sigma32 in mutant-- extragenic suppressor; may modulate RNAse III lethal action |
| *yphF* | b2548 | Low-Trans | Low-Exp/Low-Trans | putative LACI-type transcriptional regulator |
| *srmB* | b2576 | Low-Stat | Low-Exp | ATP-dependent RNA helicase |
| *yfiP* | b2583 | Low-Trans | High-Stat | orf; hypothetical protein |
| *clpB* | b2592 | High-Exp | Low-Stat | heat shock protein |
| *recN* | b2616 | Low-Exp | Low-Exp | protein used in recombination and DNA repair |
| *yfjP* | b2632 | High-Trans | Low-Trans | putative GTP-binding protein |
| *nrdI* | b2674 | High-Exp | Low-Stat | orf; hypothetical protein |
| *proW* | b2678 | Low-Exp | Low-Trans | high-affinity transport system for glycine betaine and proline |
| *hycF* | b2720 | High-Exp | Low-Exp | probable iron-sulfur protein of hydrogenase 3 (part of FHL complex) |
| *hypB* | b2727 | High-Exp | High-Exp/High-Trans | guanine-nucleotide binding protein; functions as nickel donor for large subunit of hydrogenase 3 |
| *mutS* | b2733 | Low-Exp | Low-Exp | methyl-directed mismatch repair |
| *b2832* | b2832 | Low-Stat | Low-Trans/Low-Stat | putative transport protein |
| *exbD* | b3005 | High-Exp | High-Exp | uptake of enterochelin-- tonB-dependent uptake of B colicins |
| *exbB* | b3006 | High-Exp | High-Exp | uptake of enterochelin-- tonB-dependent uptake of B colicins |
| *ygjG* | b3073 | Low-Exp | High-Trans | probable ornithine aminotransferase |
| *ygjJ* | b3079 | Low-Stat | Low-Stat | orf; hypothetical protein |
| *yhbW* | b3160 | High-Trans | High-Exp | putative enzyme |
| *folP* | b3177 | High-Trans | Low-Exp | 7;8-dihydropteroate synthase |
| *yrbD* | b3193 | High-Trans | High-Stat | orf; hypothetical protein |
| *yhbG* | b3201 | High-Trans | Low-Exp | putative ATP-binding component of a transport system |
| *nanA* | b3225 | High-Trans | High-Trans | N-acetylneuraminate lyase (aldolase)-- catabolism of sialic acid-- not K-12? |
| *yhdX* | b3269 | Low-Trans/Low-Stat | Low-Trans | putative transport system permease protein |
| *hofF* | b3327 | High-Trans | High-Exp | putative general protein secretion protein |
| *yhfO* | b3372 | High-Trans | High-Trans/High-Stat | orf; hypothetical protein |
| *trpS* | b3384 | Low-Stat | High-Exp | tryptophan tRNA synthetase |
| *slp* | b3506 | Low-Exp/Low-Trans | High-Trans/High-Stat | outer membrane protein induced after carbon starvation |
| *yhiF* | b3507 | Low-Exp | High-Trans | orf; hypothetical protein |
| *hdeD* | b3511 | Low-Exp | High-Trans | orf; hypothetical protein |
| *yhiE* | b3512 | Low-Exp | High-Trans | orf; hypothetical protein |
| *yhiW* | b3515 | Low-Exp | High-Trans | putative ARAC-type regulatory protein |
| *yhjD* | b3522 | High-Exp/High-Trans | High-Stat | orf; hypothetical protein |
| *yhjS* | b3536 | Low-Exp | High-Exp | putative protease |
| *xylA* | b3565 | Low-Stat | High-Stat | D-xylose isomerase |
| *xylR* | b3569 | Low-Trans | High-Exp | putative regulator of xyl operon |
| *selA* | b3591 | High-Exp | High-Exp | selenocysteine synthase: L-seryl-tRNA (Ser) selenium transferase |
| *yibA* | b3594 | Low-Exp | High-Trans | orf; hypothetical protein |
| *mtlA* | b3599 | High-Exp | High-Trans | PTS system; mannitol-specific enzyme IIABC components |
| *htrL* | b3618 | Low-Exp | Low-Stat | involved in lipopolysaccharide biosynthesis |
| *ibpB* | b3686 | High-Stat | Low-Stat | heat shock protein |
| *ibpA* | b3687 | High-Stat | Low-Exp | heat shock protein |
| *b3694* | b3694 | High-Exp | Low-Exp | putative FADA-type transcriptional regulator |
| *asnA* | b3744 | High-Exp | Low-Stat | asparagine synthetase A |
| *rbsC* | b3750 | Low-Trans | Low-Exp/Low-Trans | D-ribose high-affinity transport system |
| *rfe* | b3784 | High-Trans | High-Exp | UDP-GlcNAc:undecaprenylphosphate GlcNAc-1-phosphate transferase-- synthesis of enterobacterial common antigen (ECA) |
| *yigF* | b3817 | Low-Exp | Low-Stat | orf; hypothetical protein |
| *fpr* | b3924 | High-Exp | High-Exp | ferredoxin-NADP reductase |
| *yjaD* | b3996 | Low-Stat | Low-Exp | orf; hypothetical protein |
| *soxS* | b4062 | High-Exp | High-Exp | regulation of superoxide response regulon |
| *yjcZ* | b4110 | Low-Exp | High-Exp | orf; hypothetical protein |
| *proP* | b4111 | High-Trans | High-Exp | low-affinity transport system-- proline permease II |
| *yjdH* | b4125 | High-Exp | High-Exp | putative 2-component sensor protein |
| *mopA* | b4143 | High-Exp | Low-Stat | GroEL; chaperone Hsp60; peptide-dependent ATPase; heat shock protein |
| *ytfL* | b4218 | Low-Trans | Low-Exp/Low-Trans | putative transport protein |
| *ytfM* | b4220 | High-Stat | Low-Trans | orf; hypothetical protein |
| *intB* | b4271 | High-Stat | High-Exp | prophage P4 integrase |
| *yjhC* | b4280 | Low-Trans | Low-Exp | putative dehydrogenase |
| *yjhE* | b4282 | Low-Exp/High-Trans | High-Trans | orf; hypothetical protein |
| *fimI* | b4315 | Low-Stat | Low-Exp | fimbrial protein |
| *fimC* | b4316 | Low-Exp | Low-Exp/Low-Stat | periplasmic chaperone; required for type 1 fimbriae |
| *uxuA* | b4322 | High-Trans | Low-Stat | mannonate hydrolase |
| *yjjI* | b4380 | High-Exp | High-Exp | orf; hypothetical protein |
