# Supplementary material for: The HU Regulon Is Composed of Genes Responding to Anaerobiosis, Acid Stress, High Osmolarity and SOS Induction
Source: PLoS One. 2009 Feb 4;4(2):e4367. doi: 10.1371/journal.pone.0004367 (PMC2634741; doi:10.1371/journal.pone.0004367)
Supplement: Table S18 — Comparison of the genes regulated by HU(1), H-NS (2) and FIS(3) (Blot et al, 2006) (0.07 MB DOC) [file pone.0004367.s020.doc]

**Supplemental Table S18. Comparison of the genes regulated by HU^(1)^, H-NS ^(2)^ and FIS^(3)^ (Blot *et al,* 2006)**

| **Gene** | **Blattner** | **Reg.^1^** | **Reg.^2^** | **Reg.^3^** | **Function** |
| --- | --- | --- | --- | --- | --- |
| *entA* | b0596 | [Cluster2] | High-Exp/High-Trans | High-Trans | 2;3-dihydro-2;3-dihydroxybenzoate dehydrogenase; enterochelin biosynthesis |
| *hspQ* | b0966 | [Cluster2] | High-Stat | Low-Exp | orf; hypothetical protein |
| *bdm* | b1481 | [Cluster2] | Low-Stat | Low-Exp | orf; hypothetical protein |
| *gadC* | b1492 | [Cluster2] | Low-Exp/Low-Stat | Low-Exp/High-Trans | acid sensitivity protein; putative transporter |
| *ydeI* | b1536 | [Cluster2] | Low-Exp/Low-Stat | High-Trans/High-Stat | orf; hypothetical protein |
| *slp* | b3506 | [Cluster2] | Low-Exp/Low-Trans | High-Trans/High-Stat | outer membrane protein induced after carbon starvation |
| *dctR* | b3507 | [Cluster2] | Low-Exp | High-Trans | orf; hypothetical protein |
| *hdeD* | b3511 | [Cluster2] | Low-Exp | High-Trans | orf; hypothetical protein |
| *gadE* | b3512 | [Cluster2] | Low-Exp | High-Trans | orf; hypothetical protein |
| *gadW* | b3515 | [Cluster2] | Low-Exp | High-Trans | putative ARAC-type regulatory protein |
| *hypB* | b2727 | [Cluster4] | High-Exp | High-Exp/High-Trans | guanine-nucleotide binding protein; functions as nickel donor for large subunit of hydrogenase 3 |
| *nanA* | b3225 | [Cluster4] | High-Trans | High-Trans | N-acetylneuraminate lyase (aldolase)-- catabolism of sialic acid-- not K-12? |
| *yjjI* | b4380 | [Cluster4] | High-Exp | High-Exp | orf; hypothetical protein |
| *pps* | b1702 | [Cluster5] | Low-Trans | Low-Exp | phosphoenolpyruvate synthase |
| *iscS* | b2530 | [Cluster5] | High-Trans | Low-Exp | putative aminotransferase |
| *clpB* | b2592 | [Cluster5] | High-Exp | Low-Stat | heat shock protein |
| *proW* | b2678 | [Cluster5] | Low-Exp | Low-Trans | high-affinity transport system for glycine betaine and proline |
| *ibpB* | b3686 | [Cluster5] | High-Stat | Low-Stat | heat shock protein |
| *ibpA* | b3687 | [Cluster5] | High-Stat | Low-Exp | heat shock protein |
| *groL* | b4143 | [Cluster5] | High-Exp | Low-Stat | GroEL; chaperone Hsp60; peptide-dependent ATPase; heat shock protein |
| *fimI* | b4315 | [Cluster5] | Low-Stat | Low-Exp | fimbrial protein |
| *fimC* | b4316 | [Cluster5] | Low-Exp | Low-Exp/Low-Stat | periplasmic chaperone; required for type 1 fimbriae |
| *flgB* | b1073 | [Cluster7] | Low-Trans | Low-Stat | flagellar biosynthesis; cell-proximal portion of basal-body rod |
| *ftnA* | b1905 | [Cluster7] | Low-Exp | Low-Exp | cytoplasmic ferritin (an iron storage protein) |
| *fruB* | b2169 | [Cluster7] | Low-Exp | High-Trans | PTS system; fructose-specific IIA/fpr component |
| *asnA* | b3744 | [Cluster7] | High-Exp | Low-Stat | asparagine synthetase A |
