# Supplementary material for: The HU Regulon Is Composed of Genes Responding to Anaerobiosis, Acid Stress, High Osmolarity and SOS Induction
Source: PLoS One. 2009 Feb 4;4(2):e4367. doi: 10.1371/journal.pone.0004367 (PMC2634741; doi:10.1371/journal.pone.0004367)
Supplement: Table S19 — Comparison of the HU regulon with the genes located in the chromosomal areas exhibiting maximum DNA distorsion reported by Pedersen et al, (2000). (0.06 MB DOC) [file pone.0004367.s021.doc]

| **Supplemental Table S19.** Comparison of the HU regulon with the genes located in the chromosomal areas exhibiting maximum DNA distorsion reported by Pedersen et al, (2000). | | | | |
| --- | --- | --- | --- | --- |
| **Pedersen et al (2000)** | | **This work** | |  |
| **Gene** | **Blattner** | **Gene** | **Blattner** |  |
| *lpdA* | b0016 | *dnaJ* | b0015 |  |
| *yafT* | b0217 | - | - |  |
| *yagG* | b0270 | - | - |  |
| *nohB* | b0560 | *ybcW* | b0559 |  |
| b0725 | b0725 | b0725 | b0725 |  |
| b0832 | b0832 | - | - |  |
| *csgD* | b1040 | - | - |  |
| *ymfD* | b1137 | *ymfJ* | b1141 |  |
| *ycgZ* | b1164 | *ycgZ* | b1164 |  |
| *y121-2* | b1403 | - | - |  |
| *rhsE* | b1456 | *yncG* | b1454 |  |
| *ydeI* | b1536 | *ydeI* | b1536 |  |
| *ydfO* | b1549 | - | - |  |
| *celB* | b1735 | *katE* | b1732 |  |
| *flu* | b2000 | - | - |  |
| *wbbK* | b2032 | - | - |  |
| *ais* | b2252 | - | - |  |
| *nuoI* | b2281 | - | - |  |
| b2339 | b2339 | - | - |  |
| b2372 | b2372 | - | - |  |
| b2651 | b2651 | - | - |  |
| *ygeG* | b2851 | - | - |  |
| b2856 | b2856 | - | - |  |
| *tdcR* | b3119 | *tdcA* | b3118 |  |
| *rpsO* | b3165 | *deaD* | b3162 |  |
| *rpsQ* | b3311 | - | - |  |
| *yhhZ* | b3442 | - | - |  |
| *nikE* | b3480 | - | - |  |
| *rfaJ* | b3626 | - | - |  |
| *atpI* | b3739 | *atpB* | b3738 |  |
| *rpoB* | b3987 | - | - |  |
| *tyrB* | b4054 | *aphA* | b4055 |  |
| *yjcF* | b4066 | *nrfA* | b4070 |  |
| *phnI* | b4099 | - | - |  |
| *yjhA* | b4311 | *nanC* | b4311 |  |
| *hsdS* | b4348 | *yjiX* | b4353 |  |
