# Supplementary material for: The HU Regulon Is Composed of Genes Responding to Anaerobiosis, Acid Stress, High Osmolarity and SOS Induction
Source: PLoS One. 2009 Feb 4;4(2):e4367. doi: 10.1371/journal.pone.0004367 (PMC2634741; doi:10.1371/journal.pone.0004367)
Supplement: Table S20 — Oligonucleotides used for PCR amplification. (0.03 MB DOC) [file pone.0004367.s022.doc]

**Supplemental Table S20.** Oligonucleotides used for PCR amplification.

| **Gene** | **Primer** | **Sequence^a^** |
| --- | --- | --- |
| *sulA* | sulA1  sulA2 | CGGAATTCgatttcgccatagactttc  GAAGATCTgataagcccggctgtagtg |
| *dinI* | dinI1  dinI2 | CGGAATTCgtgacacatgcagatatcg  CGGGATCCccagctggcaatggagaag |
| *lldP* | lldP1  lldP2 | CGGAATTCgagcaggctgatatgggc  CGGGATCCgggaagcgatgctatcagac |
| *ndk* | ndk1  ndk2 | CGGAATTCctgccggagcaggaaggc  CGGGATCCcctgcagcttcaaagcgc |
| *nirB* | nirB1  nirB2 | CGGAATTCgggcttcttctccagcgc  CGGGATCCcttcgcgcaccagcgacag |
| *narG* | narG1  narG2 | CGGAATTCccgaaagcgtttggtagc  CGGGATCCggcgatatccatcctccc |
| *dcuC* | dcuC1  dcuC2 | CGGAATTCtcacgttcatttgtgacc  CGGGATCCgatgtagcgagctacaccc |

^a^ For each gene, the underlined sequences refer to EcoRI and BamHI sites respectively, sites except for the *sulA* gene where BamHI is replaced by BglII. The sequences in lower case correspond to the *E. coli* genome.
